# New Hybrid Compounds Incorporating Natural Products as Multifunctional Agents against Alzheimer’s Disease

**DOI:** 10.3390/pharmaceutics15102369

**Published:** 2023-09-22

**Authors:** Lidia Ciccone, Caterina Camodeca, Nicolò Tonali, Lucia Barlettani, Armando Rossello, Carole Fruchart Gaillard, Julia Kaffy, Giovanni Petrarolo, Concettina La Motta, Susanna Nencetti, Elisabetta Orlandini

**Affiliations:** 1 Department of Pharmacy, University of Pisa, Via Bonanno 6, 56126 Pisa, Italy; caterina.camodeca@unipi.it (C.C.); l.barlettani@studenti.unipi.it (L.B.); armando.rossello@unipi.it (A.R.); giovanni.petrarolo@phd.unipi.it (G.P.); concettina.lamotta@unipi.it (C.L.M.); 2 CNRS, BioCIS, Bâtiment Henri Moissan, Université Paris-Saclay, 17 Av. des Sciences, 91400 Orsay, France; nicolo.tonali@universite-paris-saclay.fr (N.T.); julia.kaffy@universite-paris-saclay.fr (J.K.); 3 Research Center “E. Piaggio”, University of Pisa, 56122 Pisa, Italy; elisabetta.orlandini@unipi.it; 4 CEA, INRAE, Département Médicaments et Technologies pour la Santé (DMTS), SIMoS, Université Paris Saclay, 91191 Gif-sur-Yvette, France; carole.fruchart@cea.fr; 5 Department of Earth Sciences, University of Pisa, Via Santa Maria 53, 56126 Pisa, Italy

**Keywords:** neuroprotection, amyloid β (Aβ), chelating agents, antioxidant, Transthyretin, TTR, positive cross-interaction, Aβ_1-40_

## Abstract

A series of new hybrid derivatives **1a**–**c**, **2a**–**c**, **3a**–**c**, **4a**–**c**, **5a**–**c**, inspired by nature, were synthesized and studied as multifunctional agents for the treatment of Alzheimer’s disease (AD). These compounds were designed to merge together the trifluoromethyl benzyloxyaminic bioactive moiety, previously identified, with different acids available in nature. The ability of the synthesized compounds to chelate biometals, such as Cu^2+^, Zn^2+^ and Fe^2+^, was studied by UV–Vis spectrometer, and through a preliminary screening their antioxidant activity was evaluated by DPPH. Then, selected compounds were tested by in vitro ABTS free radical method and ex vivo rat brain TBARS assay. Compounds **2a**–**c**, combining the strongest antioxidant and biometal chelators activities, were studied for their ability to contrast Aβ_1-40_ fibrillization process. Finally, starting from the promising profile obtained for compound **2a**, we evaluated if it could be able to induce a positive cross-interaction between transthyretin (TTR) and Aβ in presence and in absence of Cu^2+^.

## 1. Introduction

Alzheimer’s disease (AD) is a neurodegenerative disorder that leads to progressive loss of memory, decline in language skills and in other cognitive functions that certainly induce a downfall in patients’ ability to live in society [1]. Much experimental evidence has suggested that AD is a multifactorial illness characterized by a detrimental reduction of acetylcholine levels combined with high oxidative stress (OS) and dyshomeostasis of biometals [2,3,4]. Metal ions such as Cu, Fe and Zn are fundamental for several physiological functions, especially in the central nervous system; however, studies report that their dysregulation is involved in AD [5,6,7]. A high concentration of Cu, Fe and Zn was found in the brain of AD patients (300–400 μM) [8].

The main hallmarks of AD are the extracellular senile plaques, formed by β–amyloid (Aβ), and the intracellular neurofibrillary tangles composed by hyperphosphorylated τ–protein [9,10,11].

Usually, amyloid aggregates are characterized not only by a single type of amyloid protein, but by different proteins. It has been reported that, in amyloid plaque, Aβ is just the major amyloid protein found with respect to the more than a hundred proteins present [12]. This aspect is related to the cross-interaction that can occur among the proteins, and it can have negative or positive effects [13,14,15]. The negative protein–protein cross-interaction promotes the aggregation, increases the toxicity of aggregates and inhibits the degradation of aggregates (Aβ and τ–protein) [16,17,18], while the positive protein–protein cross-interaction contrasts the formation of amyloid oligomers or amyloid fibrils, reduces the toxicity of aggregates, and promotes the degradation and the dissociation of aggregates [19]. It is possible that an intrinsically amyloidogenic protein can be able to inhibit the amyloid fibril formation of another amyloidogenic protein, and, at the same time, it is able to reduce the toxicity of the aggregate. This is the effect that transthyretin (TTR) has when it binds Aβ in the brain [20,21,22,23,24].

TTR is a plasma homotetrameric protein involved in the transport of the thyroxine (T_4_) and the retinol, through retinol-binding protein, in blood and cerebrospinal fluid (CSF) [25]. The T_4_ directly binds TTR occupying a channel, characterized by two hydrophobic binding pockets (T_4_-BPs), that crosses along the entire tetramer. Each T_4_-BP is composed by three pairs of sub-sites named halogen binding pockets (HBPs) because of the iodine atoms of the endogen ligand T_4_ accommodate these hydrophobic depressions [26]. In plasma, the amount of T_4_ bound to TTR is between 10–15%, and the majority of T_4_-BPs are empty and available to bind other molecules [25]. In elderly people, wild type TTR (wt-TTR) can lose its tetrameric structure and this triggers the fibril formation [27,28,29]. The stabilization of the TTR tetramer by small molecules able to bind the T_4_-BP is one of the therapeutic strategies used to contrast the TTR fibril formation [30,31,32,33]. TTR, as well as Aβ, is sensitive to reactive oxygen species (ROS) due to the presence on each monomer of TTR of one Cys10 solvent exposed that can be easily oxidized inducing the destabilization of the tetrameric structure [34,35]. In contrast with the intrinsic amyloidogenic nature of wt-TTR, several studies have reported that under physiological conditions, TTR binds Aβ and inhibits the formation of fibril aggregates in the brain [36,37,38,39,40,41].

The approved drugs available for the treatment of AD include two disease-modifying monoclonal antibodies directed against the aggregated forms of Aβ (Aducanumab and Lecanemab), and four symptomatic treatments (donepezil, galantamine, rivastigmine, memantine), alone or in combination [42,43]. However, due to the multifactorial aspect of AD, none of these treatments is curative, especially for the loss of efficacy over time, and the lack of an efficient blood-brain barrier passage. Therefore, alternative therapeutic approaches such as the use of a multifunctional compounds that simultaneously act by inhibiting Aβ aggregation, contrasting ROS, chelating biometals and inducing positive protein–protein cross interaction are currently a fertile ground for innovative research [44,45,46].

Natural bioactive compounds extracted from plants and organisms, commonly named nutraceuticals, have been used in traditional medicine, handing over their use from one generation to another [47,48,49]. The multifunctional character of natural products has drawn the attention of the researchers because these molecules, and their derivatives, are able to simultaneously act on several targets of AD. Recently, many natural products are placed under investigation in pre-clinical and clinical trials in the treatment of AD [50,51,52].

**Figure 1:**
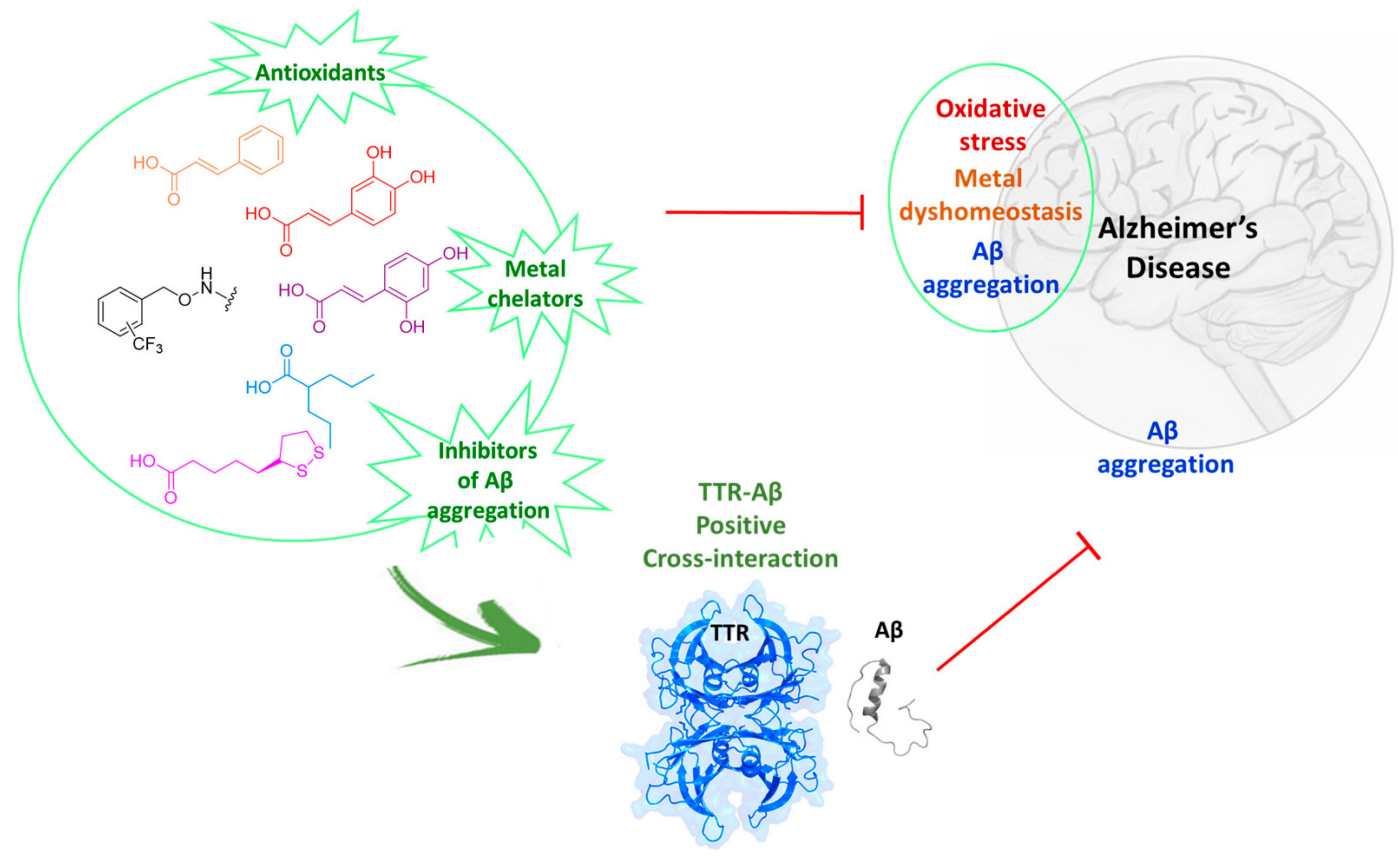
Overview of natural-products-based hybrid compounds as potential agents against AD.

The synthesis of bifunctional conjugates of natural products represents one of the growing areas in modern drug discovery. With this aim, several natural-products-based hybrid compounds have been developed as new agents to contrast AD progression [53,54,55]. In a previous work, we synthesized ferulic acid (FA) hybrids as potential multifunctional compounds suitable in the context of multifactorial diseases like AD. Among the FA hybrids, we have identified the good ability of the trifluoromethyl benzyloxyamidic moiety to reduce the Aβ_1-40_ fibrillization especially in presence of Cu^2+^ [56]. In this context, the conjugation of natural acids with the trifluoromethyl benzyloxyaminic bioactive group has led to a series of new benzyloxyamidic derivatives **1a–c**, **2a–c**, **3a–c**, **4a–c**, **5a–c**, which were characterized and studied as potential multifunctional agents for the treatment of AD, Figure 1.

The new hybrid compounds were designed merging together the previously identified trifluoromethyl benzyloxyaminic moiety, with different acids. i.e., cinnamic acid (**1a**–**c**), caffeic acid (**2a**–**c**), umbellic acid (**3a**–**c**), valproic acid (**4a**–**c**), and lipoic acid (**5a**–**c**). The cinnamic acid, caffeic acid and umbellic acid were selected in order to investigate the influence of the absence or the presence of more than one hydroxyl group on the new hybrids compare to the FA ones, while valproic acid and lipoic acid were chosen for their known properties to contrast the AD progression [57,58].

The ability of the new synthesized compounds **1**–**5** to chelate biometals, such as Cu^2+^, Zn^2+^ and Fe^2+^, was studied by UV–Vis spectrometer. Then, the antioxidant activity was investigated by in vitro DPPH assay and ex vivo TBARS colorimetric test in rat brains. Combining the results, compounds **2a**–**c**, the strongest antioxidant and biometal chelators, were selected for studying their ability to contrast Aβ aggregation. Finally, we have also investigated whether the compounds **2a**–**c** participate to the positive cross-interaction between TTR and Aβ in presence and in absence of Cu^2+^.

## 2. Materials and Methods

### 2.1. Chemistry

Analytical grade reagents and solvents were bought from Sigma-Aldrich (St. Louis, MO, USA) and were used without any purification. Each chemical reactions were followed by Thin Layer Chromatography (TLC) on 0.25 mm aluminum plates, pre-coated with silica gel and containing a fluorescent indicator (Merck Silica Gel 60 F254, Darmstadt, Germany). The UV lamp (254 nm) was used to visualize the spots on TLC. The organic solutions were dehydrated using Na_2_SO_4_, then the evaporation was performed under vacuum conditions in a rotating evaporator. The crude products were purified by flash column chromatography (Kieselgel 40, 0.040−0.063 mm, Merck, Darmstadt, Germany) or using ISOLUTE Flash Si II column cartridges (Biotage, Uppsala, Sweden).

The structural characterization of compounds and their purity were performed by the determination of melting points, by NMR and Mass Spectrometry techniques. Melting points (m.p.) were measured with a Leica Galen III microscope, and ^1^H and ^13^C NMR spectra were recorded using a Bruker Ultrashield™ 400 MHz (Fällander, Switzerland), at 25 °C. Chemical shifts (δ) are reported in ppm and coupling constant values (*J*) are in hertz (Hz). Signals in NMR spectra are indicated by the following abbreviations: s = singlet, d = doublet, m = multiplet, dd = doublet of doublet, q = quartet, sex = sextet, sept = septet, bs (broad signal). For derivatives **1a**–**c**, **4a**–**c** and **5a**–**c**, the high-resolution mass spectra were obtained using a TOF LCT Premier apparatus (Waters) with an electrospray ionization source (Agilet Technologies, Santa Clara, CA, USA). While, for compounds **2a**–**c** and **3a**–**c** the ESI-MS spectra were recorded by direct injection at 5 (positive) and 7 (negative) μL min^−1^ flow rate in an Orbitrap high-resolution mass spectrometer (Thermo, San Jose, CA, USA), equipped with HESI source.

General Procedure for the Synthesis of Benzyloxyamidic Derivatives (**1a–c**, **2a–c**, **3a–c**, **4a–c**, **5a–c**)

A solution of commercially available acids **7–11**, (1 eq) in anhydrous DMF (2 mL) under inert nitrogen atmosphere, was added by hydroxybenzotriazole (HOBt) (1.2 eq), *N*-methylmorpholine (3 eq), the opportune benzylhydroxylamine hydrochloride **6a–c** (3.1 eq) and *N*-(3-dimethylaminopropyl)-*N’*-ethylcarbodiimide hydrochloride (EDCI) (1.4 eq) [56]. The mixture obtained was stirred at room temperature (r.t.) and monitored by TLC. Then, the solution was extracted with AcOEt and washed with H_2_O. The organic portion was dried, filtered, and evaporated to furnish the crude derivatives that was purified by trituration or ISOLUTE Flash Si II column cartridges afforded the desired hybrid compounds **1a**–**c**, **2a**–**c**, **3a**–**c**, **4a**–**c**, **5a**–**c**. The ^1^H-NMR, ^13^C-NMR and ESI-MS spectra of all compounds are available in supplementary materials (Spectras S1–S45).

*N*-((2-(trifluoromethyl)benzyl)oxy)cinnamamide (**1a**) The crude product was triturated with hexane furnishing compound **1a** as a white solid. Yield: 47%; m.p.: 91–93 °C. ^1^H-NMR (400 MHz, CD_3_OD) δ: 7.82–7.80 (m, 1H, Ar); 7.73 (d, *J* = 8.0 Hz, 1H, Ar); 7.68 (t, *J* = 7.6 Hz, 1H, Ar); 7.62 (d, *J* = 15.7 Hz, 1H, Ar-CH=CH); 7.56–7.53 (m, 3H, Ar); 7.41–7.38 (m, 3H, Ar); 6.42 (d, *J* = 15.7 Hz, 1H, Ar-CH=CH); 5.14 (s, 2H, O-CH_2_). ^13^C NMR (100 MHz, CD_3_OD) δ: 166.3; 142.8; 135.9; 135.4; 133.4; 132.5; 131.1; 129.9; 129.5; 128.9; 126.9; 125.7 (q, ^1^*J_C-F_* = 272.0 Hz); 117.8; 75.0. *m/z* ESI-MS: [M + H]^+^ 322.11.

*N*-((3-(trifluoromethyl)benzyl)oxy)cinnamamide (**1b**) The crude product was triturated with hexane furnishing compound **1b** as a white solid. Yield: 47%; m.p.: 91–93 °C. ^1^H-NMR (400 MHz, CD_3_OD) δ: 7.80 (s, 1H, Ar); 7.72 (d, *J* = 7.6 Hz, 1H, Ar); 7.68–7.63 (m, 2H, Ar); 7.61–7.57 (m, 2H, Ar); 7.56–7.53 (m, 2H, Ar); 7.42–7.38 (m, 3H, Ar); 6.41 (d, *J* = 16.0 Hz, 1H, Ar-CH=CH); 5.01 (s, 2H, O-CH_2_). ^13^C NMR (100 MHz, CD_3_OD) δ: 166.3; 142.8; 138.4; 135.9; 133.8; 131.8 (q, ^2^*J_C-F_* = 32.0 Hz); 131.1; 130.3; 129.4; 128.9; 126.7; 126.3; 125.5 (q, ^1^*J_C-F_* = 271.0 Hz); 117.9; 78.2. *m/z* ESI-MS: [M + H]^+^ 322.11.

*N*-((4(trifluoromethyl)benzyl)oxy)cinnamamide (**1c**) The crude product was triturated with hexane furnishing compound **1c** as a white solid. Yield: 46.2%. Mp: 148–150 °C.

^1^H NMR (400 Hz, CD_3_OD): 7.71–7.64 (m, 4H, Ar); 7.57 (d, *J* = 16.0 Hz, 1H, Ar-CH=CH); 7.55–7.53 (m, 2H, Ar); 7.42–7.35 (m, 3H, Ar); 6.41 (d, *J* = 16.0 Hz, 1H, Ar-CH=CH); 5.02 (s, 2H, O-CH_2_). ^13^C NMR (100 MHz, CD_3_OD) δ: 160.1; 136.6; 135.4; 129.8; 125.4 (q, ^2^*J_C-F_* = 32.2 Hz); 124.9; 124.3; 123.8; 122.7; 120.2; 119.4 (q, ^1^*J_C-F_* = 271.3 Hz); 111.7; 72.0. *m/z* ESI-MS: [M + H]^+^ 322.11.

*(E)*-3-(3,4-dihydroxyphenyl)-*N*-((2-(trifluoromethyl)benzyl)oxy)acrylamide (**2a**) The crude product was purified by flash chromatography (CHCl_3_/MeOH 60:1) furnishing compound **2a** as a white solid. Yield: 37%; m.p.: 80–82 °C. ^1^H-NMR (400 MHz, CD_3_OD) δ: 7.80 (d, *J* = 7.6 Hz, 1H, Ar); 7.73 (d, *J* = 7.6 Hz, 1H, Ar); 7.68 (t, *J* = 7.6 Hz, 1H, Ar); 7.54 (t, *J* = 7.6 Hz, 1H, Ar); 7.48 (d, *J* = 15.5 Hz, 1H, Ar-CH=CH); 6.99 (d, *J* = 1.9 Hz, 1H, Ar); 6.90 (dd, *J*_1_ = 8.1 Hz, *J*_2_ = 1.9 Hz, 1H, Ar); 6.76 (d, *J* = 8.1 Hz, 1H, Ar); 6.16 (d, *J* = 15.5 Hz, 1H, Ar-CH=CH); 5.12 (s, 2H, O-CH_2_). ^13^C NMR (100 MHz, CD_3_CD) δ: 167.2; 149.1; 146.7; 143.4; 135.5; 133.4; 132.5; 129.9; 128.0; 127.1; 126.9; 124.4; 122.3; 116.4; 115.1; 114.2; 75.0. *m/z* ESI-MS: [M + H]^+^ 354.09.

*(E)*-3-(3,4-dihydroxyphenyl)-*N*-((3-(trifluoromethyl)benzyl)oxy)acrylamide (**2b**) The crude product was purified by ISOLUTE (CHCl_3_/MeOH 60:1) furnishing compound **2b** as a white solid. Yield: 27%; m.p.: 110–112 °C. ^1^H-NMR (400 MHz, CD_3_OD) δ: 7.78 (s, 1H, Ar); 7.72–7.70 (m, 1H, Ar); 7.67–7.65 (m, 1H, Ar); 7.60–7.56 (m, 1H, Ar); 7.47 (d, *J* = 15.6 Hz, 1H, Ar-CH=CH); 6.99 (d, *J* = 1.6 Hz, 1H, Ar); 6.90 (dd, *J*_1_ = 8.0 Hz, *J*_2_ = 1.6 Hz, 1H, Ar); 6.76 (d, *J* = 8.0 Hz, 1H, Ar); 6.17 (d, *J* = 15.6 Hz, 1H, Ar-CH=CH); 4.98 (s, 2H, O-CH_2_). ^13^C NMR (100 MHz, CD_3_OD) δ: 167.2; 149.1; 146.8; 143.4; 138.5; 133.8; 131.8 (q, ^2^*J_C-F_* = 32.0 Hz); 130.3; 128.0; 126.8; 126.3; 125.6 (q, ^1^*J_C-F_* = 271.4 Hz); 122.3; 116.5; 115.1; 114.2; 78.3. *m/z* ESI-MS: [M + H]^+^ 354.09.

*(E)*-4-(3,4-dihydroxyphenyl)-*N*-((2-(trifluoromethyl)benzyl)oxy)acrylamide (**2c**) The crude product was purified by ISOLUTE (CHCl_3_/MeOH 80:1) furnishing compound **2c** as a white solid. Yield: 17%; m.p.: 188–190 °C. ^1^H-NMR (400 MHz, CD_3_OD) δ: 7.70 (d, *J* = 8.4 Hz, 2H, Ar); 7.65 (d, *J* = 8.4 Hz, 2H, Ar); 7.48 (d, 1H, *J* = 15.6 Hz, Ar-CH=CH); 6.99 (d, *J* = 2.0 Hz, 1H, Ar); 6.90 (dd, *J*_1_ = 8.0 Hz, *J*_2_ = 2.0 Hz, 1H, Ar); 6.76 (d, *J* = 8.0 Hz, 1H, Ar); 6.16 (d, *J* = 15.6 Hz, 1H, Ar-CH=CH); 5.00 (s, 2H, O-CH_2_). ^13^C NMR (100 MHz, CD_3_OD) δ: 167.0; 148.9; 146.6; 143.2; 141.4; 131.4 (q, ^2^*J_C-F_* = 32.4 Hz); 130.3; 127.7; 126.2; 125.44 (q, ^1^*J_C-F_* = 271.3 Hz); 122.1; 116.2; 114.8; 113.9; 79.3. *m/z* ESI-MS: [M + H]^+^ 354.09.

*(E)*-3-(2,4-dihydroxyphenyl)-*N*-((2-(trifluoromethyl)benzyl)oxy)*acrylamide* (**3a**) The crude product was purified by ISOLUTE (CHCl_3_/MeOH 80:1) furnishing compound **3a** as a white solid. Yield: 20%; m.p.: 165–167 °C. ^1^H-NMR (400 MHz, CD_3_OD) δ: 7.83–7.79 (m, 2H, Ar); 7.73 (d, *J* = 7.6 Hz, 1H, Ar); 7.68 (t, *J* = 7.6 Hz, 1H, Ar); 7.55 (t, *J* = 7.6 Hz, 1H, Ar); 7.27–7.25 (m, 1H, Ar); 6.40 (d, *J* = 16.4 Hz, 1H, Ar-CH=CH); 6.31–6.29 (m, 2H, Ar); 5.12 (s, 2H, O-CH_2_). ^13^C NMR (100 MHz, CD_3_OD) δ: 168.3; 161.8; 159.9; 139.4; 135.5; 133.4; 132.4; 131.6; 129.7; 129.4; 126.9; 125.7 (q, ^1^*J_C-F_* = 273.1 Hz); 115.1; 113.6; 108.6; 103.5; 75.0. *m/z* ESI-MS: [M + H]^+^ 354.09.

*(E)*-3-(2,4-dihydroxyphenyl)-*N*-((3-(trifluoromethyl)benzyl)oxy)acrylamide (**3b**) The crude product was purified by ISOLUTE (CHCl_3_/MeOH 80:1) furnishing compound **3b** as a white solid. Yield: 24%; m.p.: 157–158 °C. ^1^H-NMR (400 MHz, CD_3_OD) δ: 7.81–7.77 (m, 2H, Ar); 7.72 (d, *J* = 7.6 Hz, 1H, Ar); 7.66 (d, *J* = 7.6 Hz, 1H, Ar); 7.58 (t, *J* = 7.6 Hz, 1H, Ar); 7.26–7.24 (m, 2H, Ar); 6.40 (d, *J* = 16.0 Hz, 1H, Ar-CH=CH); 6.30–6.29 (m, 2H, Ar); 4.98 (s, 2H, O-CH_2_). ^13^C NMR (100 MHz, CD_3_OD) δ: 166.9; 160.4; 158.5; 138.1; 132.4; 130.9; 130.6; 130.3; 128.9; 125.3; 124.8; 122.9; 113.7; 112.3; 107.2; 102.2; 76.9. *m/z* ESI-MS: [M + H]^+^ 354.09.

*(E)*-3-(2,4-dihydroxyphenyl)-*N*-((4-(trifluoromethyl)benzyl)oxy)acrylamide (**3c**) The crude product was purified by ISOLUTE (CHCl_3_/MeOH 80:1) furnishing compound **3c** as a white solid. Yield: 28%; m.p.: 176–178 °C. ^1^H-NMR (400 MHz, CD_3_OD) δ: 7.81 (d, *J* = 16.0 Hz, 1H, Ar); 7.73–7.66 (m, 4H, Ar); 7.28–7.26 (m, 1H, Ar); 6.41 (d, *J* = 16.0 Hz, 1H, Ar-CH=CH); 6.33–6.31 (m, 2H, Ar); 5.01 (s, 2H, O-CH_2_). ^13^C NMR (100 MHz, CD_3_OD) δ: 168.3; 161.8; 159.9; 141.7; 139.4; 131.7; 131,4; 130.5; 126.3; 125.7 (q, ^1^*J_C-F_* = 271.2 Hz); 115.1; 113.6; 108.6; 103.5; 78.2. *m/z* ESI-MS: [M + H]^+^ 354.09.

*2*-propyl-*N*-((2-(trifluoromethyl)benzyl)oxy)pentanamide (**4a**) The crude product was purified by ISOLUTE (hexane/AcOEt 15:1) furnishing compound **4a** as a white solid. Yield: 34%; m.p.: 73–75 °C. ^1^H-NMR (400 MHz, CD_3_OD) δ: 7.80 (d, *J* = 8.0 Hz, 1H, Ar); 7.71 (d, *J* = 8.0 Hz, 1H, Ar); 7.66 (t, *J* = 8.0 Hz, 1H, Ar); 7.52 (t, *J* = 8.0 Hz, 1H, Ar); 5.07 (s, 2H, O-CH_2_); 2.02 (sept, *J* = 4.8 Hz, 1H, CHCO); 1.59–1.50 (m, 2H, CH-CH_2_); 1.38–1.25 (m, 2H, CH-CH_2_); 1.24–1.18 (m, 4H, 2 CH_2_-CH_2_); 0.88 (t, 6H, *J*= 7.2 Hz, 2 CH_3_). ^13^C NMR (100 MHz, CD_3_OD) δ: 175.7; 135.6; 133.4; 132.1; 129.7; 129.3; 126.8; 125.7 (q, ^1^*J_C-F_* = 272.9 Hz); 74.8; 44.3; 35.0; 21.6; 14.3. *m/z* ESI-MS: [M + H]^+^ 318.17.

*2*-propyl-*N*-((3-(trifluoromethyl)benzyl)oxy)pentanamide (**4b**) The crude product was purified by ISOLUTE (hexane/AcOEt 15:1) furnishing compound **4b** as a white solid. Yield: 30%; m.p.: 74–76 °C. ^1^H-NMR (400 MHz, CD_3_OD) δ: 7.74 (s, 1H, Ar); 7.70 (d, *J* = 7.6 Hz, 1H, Ar); 7.65 (d, *J* = 7.6 Hz, 1H, Ar); 7.57 (t, *J* = 7.6 Hz, 1H, Ar); 4.93 (s, 2H, O-CH_2_); 1.97 (sept, *J* = 4.8 Hz, 1H, CHCO); 1.56–1.47 (m, 2H, CH-CH_2_); 1.35–1.29 (m, 2H, CH-CH_2_); 1.19 (sex, *J* = 7.2 Hz, 4H, 2 CH_2_-CH_2_); 0.86 (t, *J* = 7.2 Hz, 6H, 2 CH_3_). ^13^C NMR (100 MHz, CD_3_OD) δ: 175.5; 138.5; 133.9; 131.7 (q, ^2^*J*_C-F_ = 32.0 Hz); 130.3; 126.9; 126.3; 125.6 (q, ^1^*J*_C-F_ = 271.4 Hz); 77.9; 44.4; 36.0; 21.6; 14.2. *m/z* ESI-MS: [M + H]^+^ 318.17.

*2*-propyl-*N*-((4-(trifluoromethyl)benzyl)oxy)pentanamide (**4c**) The crude product was purified by ISOLUTE (hexane/AcOEt 15:1) furnishing compound **4c** as a white solid. Yield: 23%; m.p.: 78–80 °C. ^1^H-NMR (400 MHz, CD_3_OD) δ: 7.69–7.67 (m, 2H, Ar); 7.64–7.61 (m, 2H, Ar); 4.93 (s, 2H, O-CH_2_); 1.96 (sept, *J* = 4.9 Hz, 1H, CHCO); 1.56–1.47 (m, 2H, CH-CH_2_); 1.36–1.29 (m, 2H, CH-CH_2_); 1.19 (sex, *J* = 7.2 Hz, 4H, 2 CH_2_-CH_2_); 0.85 (t, *J* = 7.2 Hz, 6H, 2 CH_3_). ^13^C NMR (100 MHz, CD_3_OD) δ: 175.2; 141.4; 131.3 (q, ^2^*J*_C-F_ = 32.3 Hz); 130.3; 126.0; 125.41 (q, ^1^*J_C-F_* = 271.1 Hz); 77.6; 44.2; 35.8; 21.4; 14.0. *m/z* ESI-MS: [M + H]^+^ 318.17.

*(R)*-5-(1,2-dithiolan-3-yl)-*N*-((2-(trifluoromethyl)benzyl)oxy)pentanamide (**5a**) The crude product was purified by ISOLUTE (hexane/AcOEt in gradient 10:1, 5:1, 4:1 and 2:1) furnishing compound **5a**, vitreous solid. Yield: 39%. ^1^H-NMR (400 MHz, CDCl_3_) δ: 8.32 (bs, 1H, NHCO); 7.69–7.63 (m, 2H, Ar); 7.56–7.53 (m, 1H, Ar); 7.43–7.41 (m, 1H, Ar); 5.07 (s, 2H, O-CH_2_); 3.54–3.49 (m, 1H, CHS); 3.17–3.04 (m, 2H, CH_2_-S); 2.45–2.37 (m, 1H, H-CH-CH_2_S); 2.07 (bs, 2H, CH_2_CO); 1.90–1.82 (m, 1H, H-CH-CH_2_S); 1.70–1.55 (m, 4H, 2 CH_2_), 1.43–134 (m, 2H, CH_2_). ^13^C NMR (100 MHz, CDCl_3_) δ: 170.6; 132.1; 131.1; 128.5; 126.0; 125.4; 122.7; 74.2; 56.3; 40.2; 38.4; 34.5; 32.7; 28.7; 24.9. *m/z* ESI-MS: [M + H]^+^ 380.10.

*(R)*-5-(1,2-dithiolan-3-yl)-*N*-((3-(trifluoromethyl)benzyl)oxy)pentanamide (**5b**) The crude product was purified by ISOLUTE (hexane/AcOEt in gradient 20:1, 10:1, 5:1, 2:1) furnishing compound **5b**, vitreous solid. Yield: 29%. %. ^1^H-NMR (400 MHz, CD_3_OD) δ: 7.76 (s, 1H, Ar); 7.73–7.65 (m, 2H, Ar); 7.59–7.54 (m, 1H, Ar); 4.93 (s, 2H, O-CH_2_); 3.58–3.51 (m, 1H, CH-S); 3.20–3.06 (m, 2H, CH_2_-S); 2.44 (sex, *J* = 6.4 Hz, 1H, H-CH-CH_2_S); 2.07 (t, *J* = 7.3 Hz, 2H, CH_2_CO); 1.86 (sex, *J*= 6.4 Hz, 1H, H-CH-CH_2_S); 1.71–1.55 (m, 4H, 2 CH_2_); 1.46–128 (m, 2H, CH_2_). ^13^C NMR (100 MHz, CD_3_OD) δ: 172.8; 138.4; 133.8, 131.7 (q, ^2^*J*_C-F_ = 32.0 Hz); 130.3; 126.8; 126.3; 125.6 (q, ^1^*J*_C-F_ = 271.4 Hz); 78.0; 57.4; 41.3; 39.3; 35.6; 33.5; 29.7; 26.3. *m/z* ESI-MS: [M + H]^+^ 380.10.

*(R)*-5-(1,2-dithiolan-3-yl)-*N*-((4-(trifluoromethyl)benzyl)oxy)pentanamide (**5c**) The crude product was purified by ISOLUTE (hexane/AcOEt in gradient, 10:1, 5:1, 3:1) furnishing compound **5c**, vitreous solid. Yield: 19%. ^1^H-NMR (400 MHz, CD_3_OD) δ: 7.70–7.68 (m, 2H, Ar); 7.63–7.61 (m, 2H, Ar); 4.93 (s, 2H, O-CH_2_); 3.57–3.53 (m, 1H, CHS); 3.19–3.06 (m, 2H, CH_2_-S); 2.47 (sex, *J* = 6.4 Hz, 1H, H-CH-CH_2_S); 2.08 (t, *J* = 7.3 Hz, 2H, CH_2_CO); 1.86 (sex, *J* = 6.4 Hz, 1H, H-CH-CH_2_S); 1.71–1.55 (m, 4H, 2 CH_2_); 1.46–128 (m, 2H, CH_2_). ^13^C NMR (100 MHz, CD_3_OD) δ: 172.9; 141.6; 131.6 (q, ^2^*J*_C-F_ = 32.2 Hz); 130.6; 126.4; 125.6 (q, ^1^*J*_C-F_ = 271.0 Hz); 77.9; 57.5; 41.3; 39.4; 35.6; 33.5; 29.7, 26.3. *m/z* ESI-MS: [M + H]^+^ 380.10.

### 2.2. UV–Vis Metal Binding Studies

The synthesized compounds were tested for their metal chelating effect through spectroscopic measurements [59,60]. The UV–Vis absorbance of compounds was recorded in the absence and presence of metal ions using a UV–Vis spectrometer (a SPECTROstarNano (220–1000 nm), Ortenberg, Germany. The experiment was performed in 96-well plates and each experiment was made in triplicates. A fixed amount of investigated ligand (20 μM) solubilized in MeOH was mixed with increasing concentration of Cu^2+^ (CuCl_2_), Fe^2+^ (FeSO_4_) and Zn^2+^ (ZnCl_2_) from 0 to 80 μM and incubated for 30 min at room temperature (r.t.). The absorbance change was monitored at wavelengths ranging from 220 to 700 nm. The stoichiometry of studied compounds for Cu^2+^ and Fe^2+^ complexes was determined by employing the mole ratio method.

Due to a non-measurable UV absorbance for compounds **4a** and **5c**, a different protocol using pyrocatechol violet (PV) was applied [61]. The compounds (200 µM) incubated for 10 min with CuSO_4_ (200 µM), after 200 µM PV was added and the plate was incubated at 25 °C for 20 min. The absorption spectra were recorded. The same protocol was used for Fe^2+^ (FeSO_4_) in presence of ferrozine.

### 2.3. Investigation of Antioxidant Capacity

#### 2.3.1. DPPH Radical Scavenging Assay

The DPPH assay was carried out in a 96-microwell plate according to a previously described protocol [62]. In brief, DPPH solution (60 µM in MeOH) was added to studied compounds or MeOH as the control. The 96-microwell plate was incubated (in the dark, at r.t. for 1 h) and the absorbance values were read at 517 nm by a SPECTROstarNano (220–1000 nm) UV–Vis spectrophotometer, Ortenberg, Germany. During the first screening, all compounds were tested ad 100 µM, then for compounds **2a**–**c** the EC_50_ (effective concentration), the concentration of substrate that causes a 50% reduction in the DPPH colour, was calculated.

All experiments were made in triplicate. The percentage of the antioxidant activity (%AA) of the compounds was determined according to Equation (1):

%AA = ((Abs_DPPH_ − Abs_sample_)/Abs_DPPH_) × 100 (1)

Abs_DPPH_ is the absorbance of DPPH solution, and Abs_sample_ is the absorbance of DPPH solution containing the tested compound.

The DPPH radical scavenging ability of the most active compounds **2a**–**c** was expressed as Trolox equivalent antioxidant capacity (TEAC) value by using Trolox standard regression curve [63]. Each concentration was tested in triplicate and the EC_50_ value was reported as the mean ± the standard error of the mean (SEM) of three independent experiments.

#### 2.3.2. ABTS Assay

The ABTS assay was performed according to the methods reported in literature [64,65]. Briefly, 7 mM of ABTS was mixed with 2.45 mM of K_2_S_2_O_8_ solution, ratio 1:1, in 10 mM phosphate buffer (pH 7.4). The mixture was incubated in a dark box at r.t. for 12 h, then the stock solution was diluted until the absorbance was 0.70 ± 0.02 at 734 nm.

In a 96-well plate, 190 µL ABTS working solution and 10 µL sample solution (compounds **2a**–**c**, **3a**–**c** at concentration of 100 μM) were mixed and the absorbance was measured (734 nm) using a SPECTROstarNano (220–1000 nm) UV–Vis spectrophotometer, Ortenberg, Germany. Then, for compounds **2a**–**c** the EC_50_ was calculated and the values were expressed in TEAC equivalents [66,67]. Each concentration was tested in triplicate and the EC_50_ was reported as the mean ± SEM of three independent experiments.

#### 2.3.3. Thiobarbituric Acid Reagent Substance (TBARS) Assay

The TBARS assay was carried on in accordance with a previously published protocol [62]. Briefly, the rat brain, freshly isolated, was homogenized in 10% (*w*/*v*) phosphate buffer, pH 7.4. The stock solutions of compounds **2a**–**c** and **3a**–**c** (10 mM in DMSO) were prepared and then diluted at three different concentrations (1 mM, 100 µM, and 10 µM) with phosphate buffer pH 7.4. The experiment was performed in a falcon tube where the rat brain homogenate (100 µL) was added to FeCl_3_ (20 µM) and ascorbic acid (100 µM) with or without test compounds. The final volume of 1 mL volume was achieved with phosphate buffer at pH 7.4. All samples were heated at 37 °C and were stirred for 30 min. After this period, thiobarbituric acid (1% *w*/*v* in 0.05 N NaOH) and 25% *v*/*v* HCl were added and the falcon tubes were boiled for 10 min. Then, they were cooled in an ice-cold water bath. Finally, the extraction was executed using n-butanol (3 mL) and the tubes were centrifuged at 2000 g for 10 min. The organic portion was transferred into 96-well plates and the absorbance, at 532 nm, was recorded using a SPECTROstarNano (220–1000 nm) UV–Vis spectrophotometer, Ortenberg, Germany. The TBARS was reported as nmoles of malondialdehyde (MDA)/10 mg of rat brain tissue, by interpolation with the standard curve of 1,1,3,3-tetramethoxypropane. Data represent the mean ± SEM of the triplicate.

### 2.4. ANS Competitive Binding Assay

The binding of ANS (8-anilino-1-naphthalenesulfonic acid), Sigma-Aldrich (St. Louis, MO, USA) and its displacement by the three selected compounds **2a**–**c** were studied using Human Plasma TTR (Merck Millipore, Molsheim, France) according to the protocol previously described [68,69]. Briefly, TTR (8 µM) was incubated with ANS (16 µM) at r.t. for 15 min in 96-well plates. Then, compounds **2a**–**c** at concentrations of 100 µM were added. After 10 min, the fluorescent emission spectra (400–540 nm) were recorded exciting at 280 nm using Molecular Devices SpectraMax Gemini XPS plate reader, (San Jose, CA, USA). For compounds **2a,** the IC_50_ was calculated and value was expressed as the mean ± SEM of the triplicate. All experiments were made in triplicate and, the fluorescence increases of ANS bound to TTR solution compared to its control without TTR, and TTR alone, in phosphate buffer, were used as a control.

### 2.5. Thioflavin T (ThT) Fluorescence Assay

Aβ_1-40_ was obtained from Bachem (Bubendorf, Switzerland). To remove preformed aggregates, it was dissolved during 15–20 min at r.t. in 1% NH_4_OH to give a concentration of 2 mg/mL, followed by immediate lyophilisation. The Aβ_1-40_ peptide was then dissolved in an aqueous 1% ammonia solution to a concentration of 1 mM and then, just prior to use, was diluted to 0.2 mM with 10 mM Tris-HCl, 100 mM NaCl buffer (pH 7.4). Compounds (**2a**–**c**) were dissolved in DMSO (4.9 mM), while TTR in phosphate buffer 20 mM buffer (pH 7.4) at 1 mM concentration. ThT fluorescence was measured to evaluate the development of Aβ_1-40_ fibrils over time using a fluorescence plate reader (Vantastar, BMG lab-tech, Ortenberg, Germany) with standard 96-well black microliter plates. The ThT fluorescence intensity was recorded by using excitation/emission settings at 440−15 nm/480−15nm for 21 h and applying a double orbital shaking of 10 s before the first cycle. Experiments were triggered by adding Aβ_1-40_ achieving a final concentration of 10 μM into a mixture containing 40 μM ThT in 10 mM Tris-HCl, 100 mM NaCl buffer (pH 7.4) with and without the studied compounds (10 μM) at 25 °C, and with and without CuCl_2_ (10 µM). When TTR was assessed in the presence of compound **2a**, the ratio between TTR and **2a** was adjusted to 1:2 (TTR:**2a**) in order to let the compound interacting with both hydrophobic pockets. A time of 3 h of incubation was employed before adding the solution into the well. The *F* reduction is depicted as the intensity of experimental fluorescence plateau observed with the studied compound relative to the value obtained without the compound and is quantified as the following percentage: ((*F* Aβ_1-40_+compound − *F* Aβ_1-40_)/(*F* Aβ_1-40_)) × 100. The data of fluorescence intensity come from at least two independent experiments, where each condition has been performed in duplicate, in the case of the presence of TTR (10 µM), or triplicate, in the case of the absence of TTR (10 µM), respectively. The values are given as averaged percentage ± SEM.

### 2.6. Statistical Analysis

The statistical analysis was performed using the GraphPad Prism software, version 8.0 (GraphPad Software Inc., San Diego, CA, USA). Data were presented as the mean ± standard error (SEM) of at least two independent experiments.

## 3. Results

### 3.1. Chemistry

**Scheme 1:**
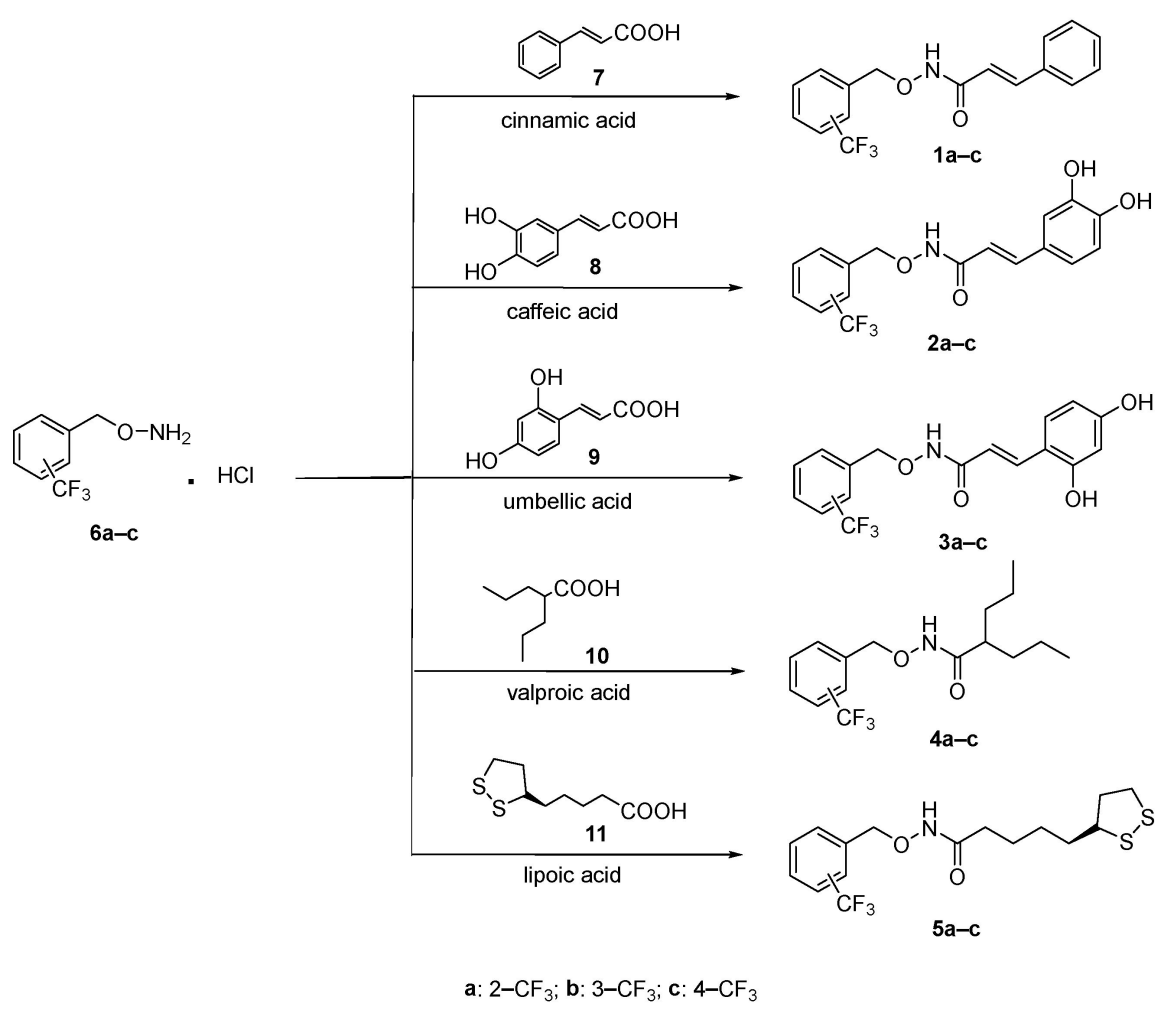
General procedure for the synthesis of compounds **1a**–**c**, **2a**–**c**, **3a**–**c**, **4a**–**c**, **5a**–**c. Reaction conditions**: EDCI, HOBt, *N*-methylmorpholine, anhydrous DMF, r.t., 24 h.

The studied trifluoromethyl benzyloxyamidic compounds **1a**–**c**, **2a**–**c**, **3a**–**c**, **4a**–**c**, **5a**–**c** (Figure S1) were synthesized according to the synthetic procedure reported in Scheme 1, following the method previously described [56]. The *O*-arylmethylhydroxylamine hydrochloride **6a**–**c** were prepared applying the synthetic route already described [70,71]. Briefly, the derivatives **6a**–**c** were obtained by reaction between the appropriate benzyl bromide (2–CF_3_, 3–CF_3_ and 4–CF_3_ substituted) and the *N*-hydroxyphalimide. Then, the deprotection of the phtalimido group was carried on by ammonia solution 7 N in MeOH and the compounds **6a**–**c** were precipitated as hydrochloride salts. The coupling reaction of the free amino group of derivatives **6a**–**c** with the appropriate natural acids **7**–**11** (**7**: cinnamic acid; **8**: caffeic acid; **9**: umbellic acid; **10**: valproic acid; **11**: lipoic acid commercially available) was carried out in anhydrous DMF and under inert argon atmosphere, in the presence of the carboxyl activating agent *N*-(3-dimethylaminopropyl)-*N*′-ethylcarbodiimide hydrochloride (EDCI), hydroxybenzotriazole (HOBt) and *N*-methylmorfoline to give the final products **1a**–**c**, **2a**–**c**, **3a**–**c**, **4a**–**c**, **5a**–**c** as white solids.

For compounds **1a–c**, **2a–c**, **3a–c**, only one of the two possible *E/Z* configurational isomers was obtained. The *E* configuration was attributed by ^1^H-NMR spectra. The characteristics proton Ar-CH=CH gave ppm values between 7.62–7.47 according to data reported in literature [56,70].

### 3.2. Metal Chelating Properties

**Figure 2:**
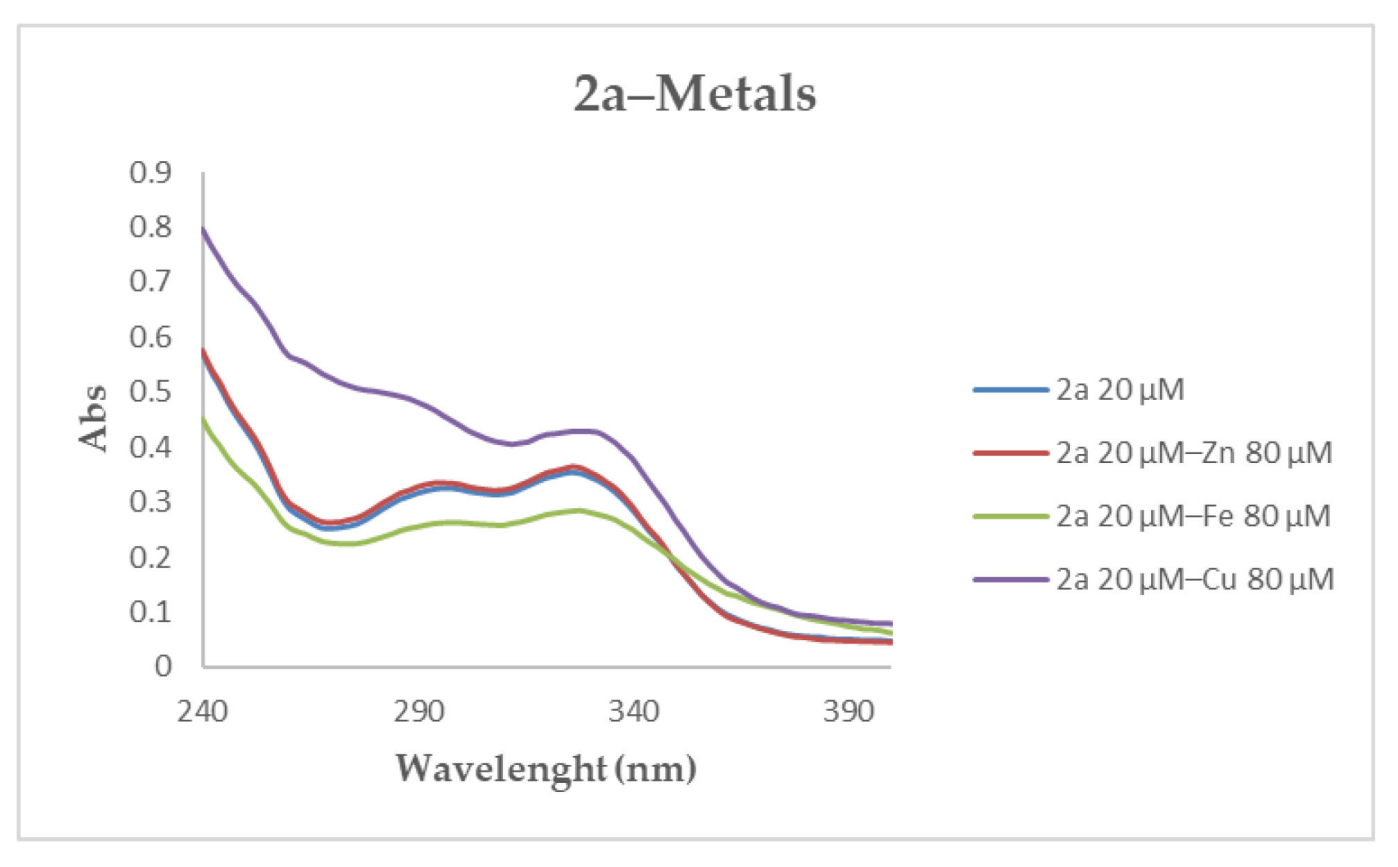
UV spectra of compound **2a** alone and in presence of Zn^2+^, Fe^2+^ and Cu^2+^ in MeOH.

The ability of the synthesized compounds **1a**–**c**, **2a**–**c**, **3a**–**c**, **4a**–**c**, **5a**–**c** to chelate biometals, such as Zn^2+^, Fe^2+^ and Cu^2+^, was studied by UV–Vis spectrometry. The first screening was executed by comparing the spectra of the o-CF_3_ substituted of each triad (**1a**, **2a**, **3a**, **4a**, **5a** at 20 μM) without metal, with those, of the same molecules, in the presence of Zn^2+^, Fe^2+^ and Cu^2+^ (80 µM) [59]. Compounds **1a**, **2a**, **3a** showed a significant shift in UV spectrum in the presence of Cu^2+^, while only compounds **2a** displayed a weak change when Fe^2+^ was added. No detectable shift for all investigated compounds was recorded in the presence of Zn^2+^. A representative spectra obtained during the first screening is reported for derivatives **2a**, Figure 2.

Due to the absence of a remarkable absorbance in the UV spectra of compounds **4a** and **5a** (Figure S2), their ability to chelate biometals was studied using the colorimetric methods that employs ferrozine and PV (pyrocatechol violet) reagents for Fe^2+^ and Cu^2+^, respectively. No interaction between **4a** and **5a** was recorded in presence of Fe^2+^, while a slight shift appeared with Cu^2+^ (Figure S3).

**Figure 3:**
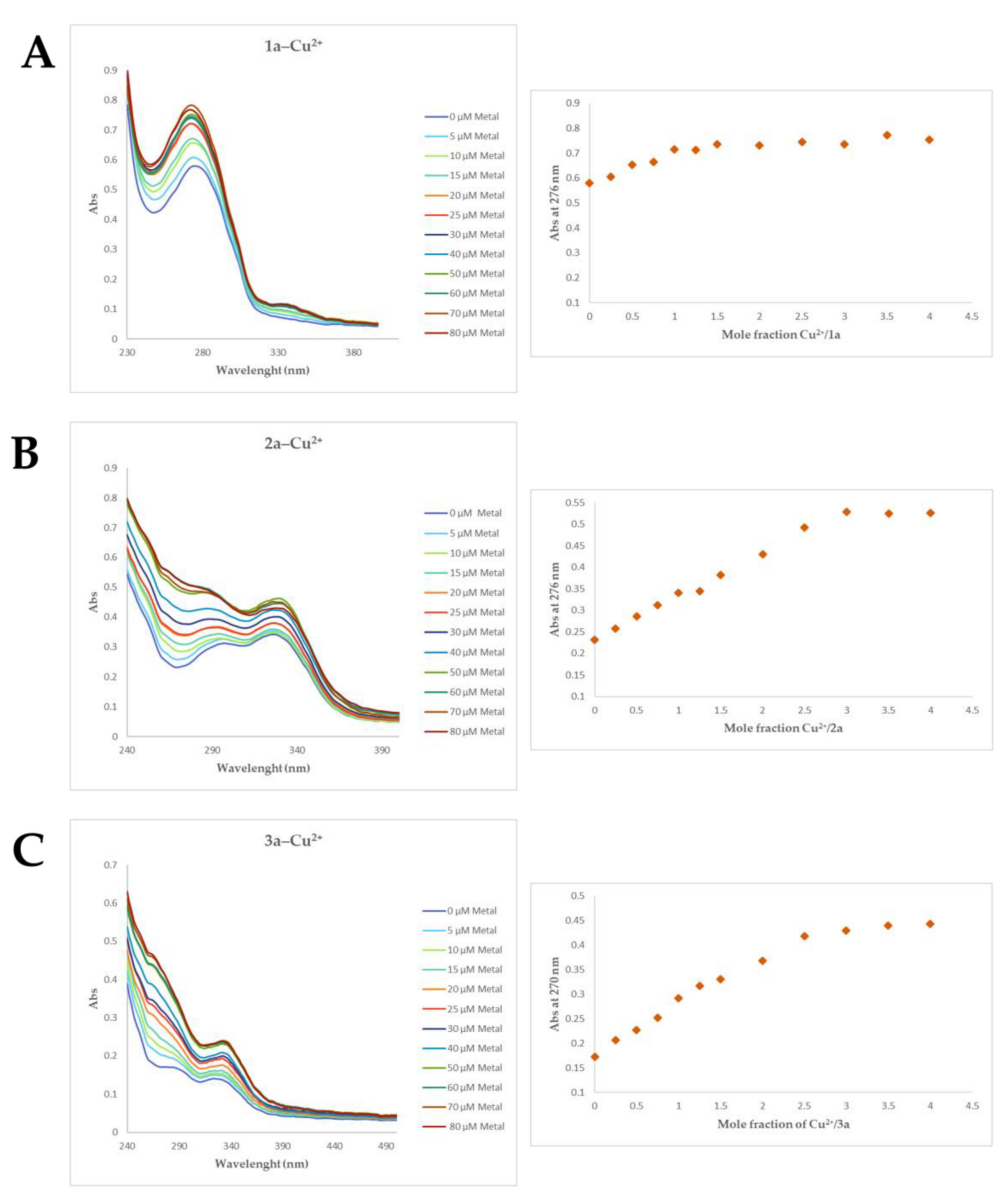
UV spectra of selected compounds and the stoichiometry of the complex [Ligand–Cu^2+^]. (**A**) **1a**–cu^2+^; (**B**) **2a**–cu^2+^ and (**C**) **3a**–cu^2+^.

Starting from these preliminary results, the derivatives **1a**, **2a**–**c**, and **3a** were selected to quantify their ability to chelate Cu^2+^ using molar ratio method. Therefore, the concentration of the ligands (**1a**, **2a**, **3a**) was the same (20 µM) in each experiment while the concentration of the Cu^2+^ was increased from 0 µM to 80 µM as shown in Figure 3A–C.

The spectra of compound **1a** in the presence of increased concentration of Cu^2+^ displayed a shift in absorbance from 0.577 to 0.755 at 276 nm. The molar fraction analysis suggested that, under the experimental conditions, the stoichiometry for the Cu^2+^–**1a** complex was 1.0, indicating a complex with 1:1 [Cu^2+^–**1a**] molar ratio, Figure 3A. Compounds **2a**–**c** and **3a**, in the presence of increasing concentration of Cu^2+^, showed a remarkable shift up in absorbance of UV spectra from 260 to 360 nm, Figure 3B,C and Figures S4 and S5. Regarding the compounds **2a**–**c** and **3a**, the stoichiometry for the [Cu^2+^–ligand] complex is around 2, suggesting a complex with 2:1 [Cu^2+^–ligand] molar ratio.

**Figure 4:**
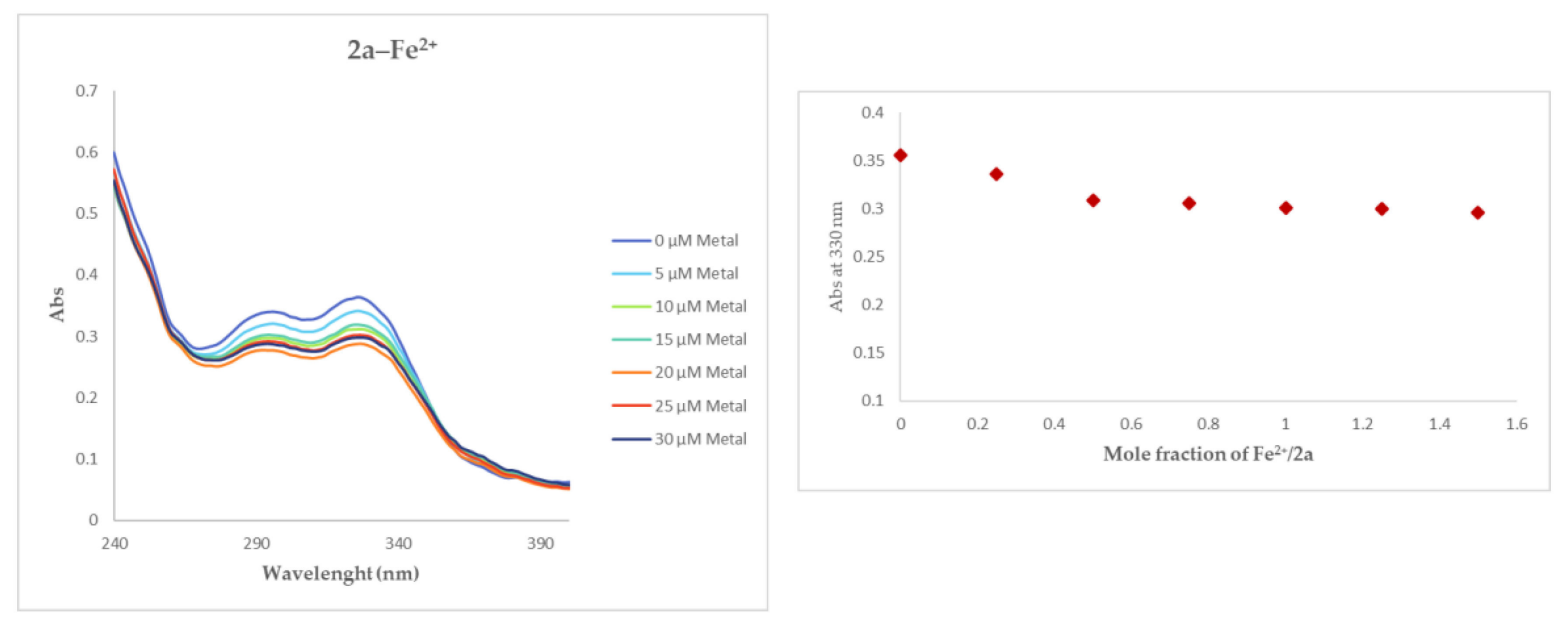
UV spectrum of compound **2a**–Fe^2+^and the stoichiometry of the complex.

During the first screening with Fe^2+^ (80 µM), compound **2a** showed a detectable shift down in absorbance at two different wavelengths: 294 and 330 nm, Figure 4. In order to determine the stoichiometry of the complex **2a**–Fe^2+^, a series of solutions where the concentration of **2a** was maintained the same while the amount of Fe^2+^ was increased from 0 to 30 µM (plateaus), were tested. As reported in Figure 4, plotting the absorbance changes at 330 nm the molar fraction found is 1, indicating a stoichiometry 1:1 of compound **2a**.

### 3.3. Antioxidant Properties of Synthesized Compounds

#### 3.3.1. DPPH Assay

The %AA of all newly synthesized compounds (**1a**–**c**, **2a**–**c**, **3a**–**c**, **4a**–**c**, **5a**–**c**) was determined using the DPPH assay. The first screening was performed by testing all derivatives at 100 µM, and compounds **2a**–**c**, with catechol, gave high %AA (**2a** 95%; **2b** 96%; **2c** 96%), compound **3a–c** gave a %AA around 60 (**3a** 59%; **3b** 60%; **2c** 58%) while no antioxidant activity was detected for compounds **1a**–**c**, **4a**–**c**, **5a**–**c** (%AA 0). Starting from these results, molecules **2a**–**c** were studied in order to quantify the EC_50_ and the values were then expressed as TEAC, Table 1. The low EC_50_ values between 6 and 8.9 µM indicate that compounds **2a**–**c** possess a greater free radical scavenging activity than the caffeic acid (EC_50_ 13.3 µM) [72].

#### 3.3.2. ABTS Assay

The synthesized compounds **2a**–**c**, **3a**–**c** were selected for a first screening at 100 µM. At this concentration, resorcinol derivatives showed good radical scavenger activity (**3a** 68%, **3b** 67%, **3c** 65%).

Then, considering the elevate antioxidant activity found for compounds **2a**–**c** (**2a** 99.1%, **2b** 98.8% and **2c** 97.3%) they were tested at different concentrations in order to determinate the EC_50_. The EC_50_ was expressed in TEAC values, as reported in Table 2. Compounds **2a** (EC_50_ 9.9 µM) and **2b** (EC_50_ 13.4 µM) showed an EC_50_ value comparable to caffeic acid (10.9 µM [72]) while derivative **2c** displayed an EC_50_ of 22.95 µM, slightly higher than parent natural acid.

#### 3.3.3. TBARS Production in the Rat Brain

**Figure 5:**
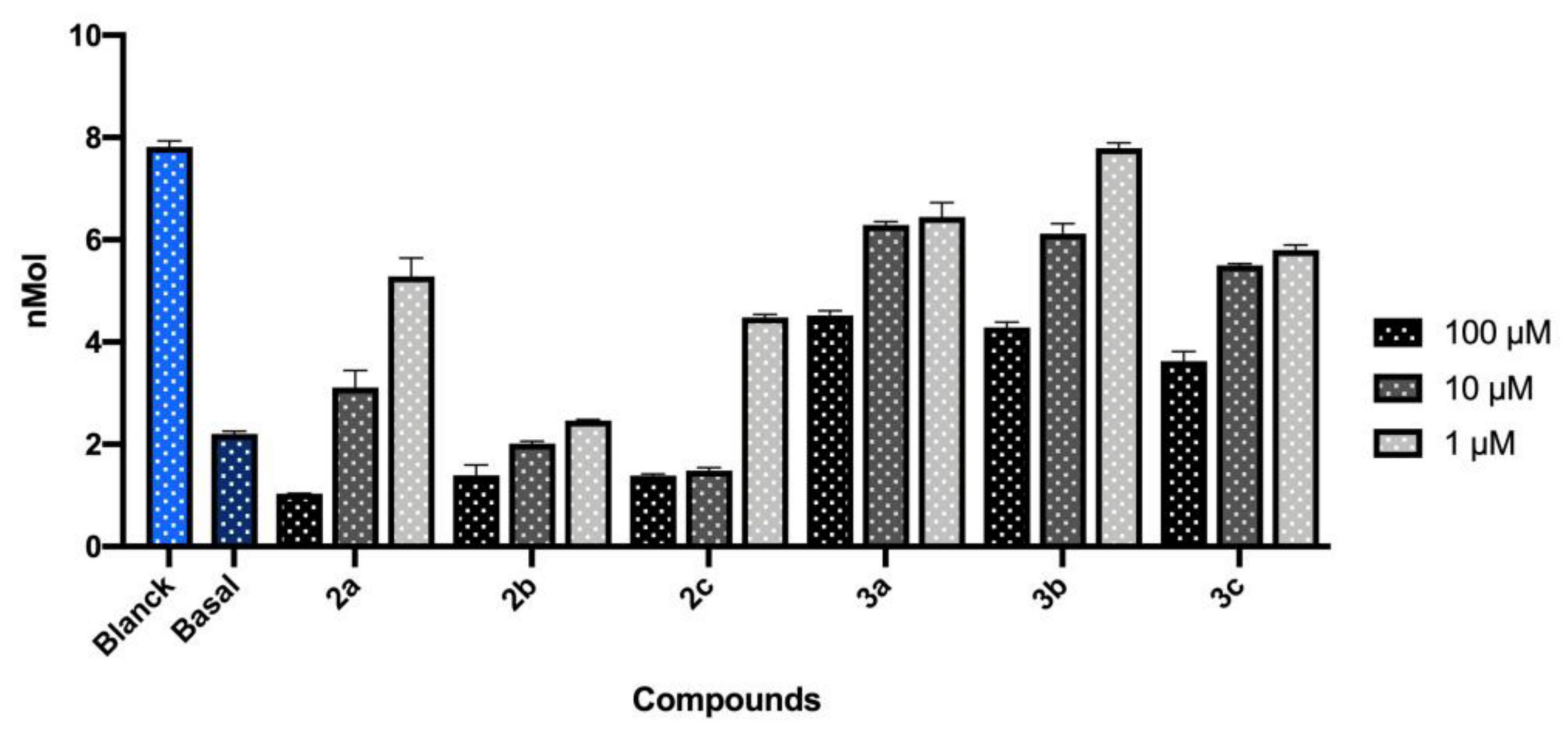
Effect of compounds **2a**–**c** and **3a**–**c** on the TBARS production in the rat brain. In light blue blank and in blue basal brain activity. Data represent the mean ± SEM of the triplicate.

The antioxidant properties of derivatives **2a**–**c** and **3a**–**c** were studied ex vivo on rat brain. The hydroxyl radical-dependent lipid peroxidation induced in rat brain homogenate by the oxidant system Fe^3+^/ascorbic acid, that initiates the Fenton reaction, was investigated in absence and in presence of selected compounds Figure 5. Compounds **2a**–**c** and **3a**–**c** were tested at three different concentrations (1 μM, 10 μM and 100 μM) and the result was expressed in nmoles of MDA in 10 mg of rat brain tissue. A slight antioxidant activity was recorded when **3a**–**c** were tested at 100 μM, while the antioxidant effect became weaker at 10 μM and it is almost lost at 1 μM. In contrast, compounds **2a**–**c** showed a good antioxidant effect at 100 μM. In particular, derivatives **2a**–**b** kept a relevant antioxidant activity at 10 μM and **2b** maintained its profile also at 1 μM.

### 3.4. Displacement of ANS

**Figure 6:**
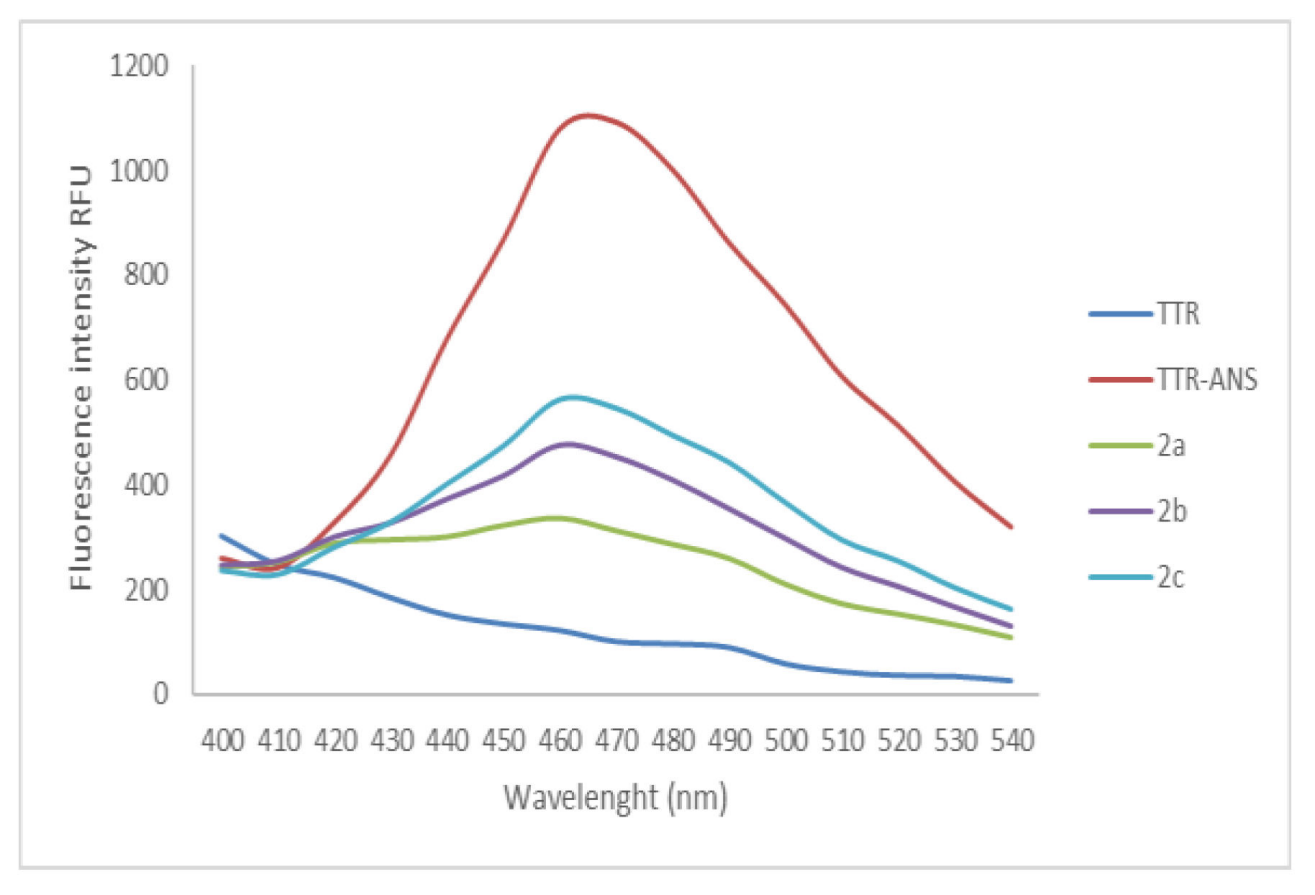
ANS displacement binding assay for compounds **2a**–**c** at tested at concentration of 100 µM.

The ability of selected compounds **2a**–**c** to bind the TTR-BP was evaluated by the ANS displacement binding test. The derivatives showed a good interaction with TTR at 100 µM, in particular compound **2a** displaces ANS of 71%, Figure 6, with an IC_50_ = 31.9 µM (±0.1 SEM).

## 4. ThT Test Fluorescence Spectroscopy Assay

According to the results obtained above, we selected compounds **2a**–**c**, the best antioxidant and chelating agents, to evenly assess their ability to reduce the fibril formation of Aβ_1-40_ peptide. The ThT assay was performed both in absence or in presence of Cu^2+^ and of TTR. We tested the effect of Cu^2+^ at stoichiometric concentration (10 µM) on the Aβ_1-40_ aggregation and we observed an increase of ThT fluorescence intensity over time, compared to Aβ_1-40_ in the absence of Cu^2+^ (Figure S6), which is consistent with the literature data [73].

**Figure 7:**
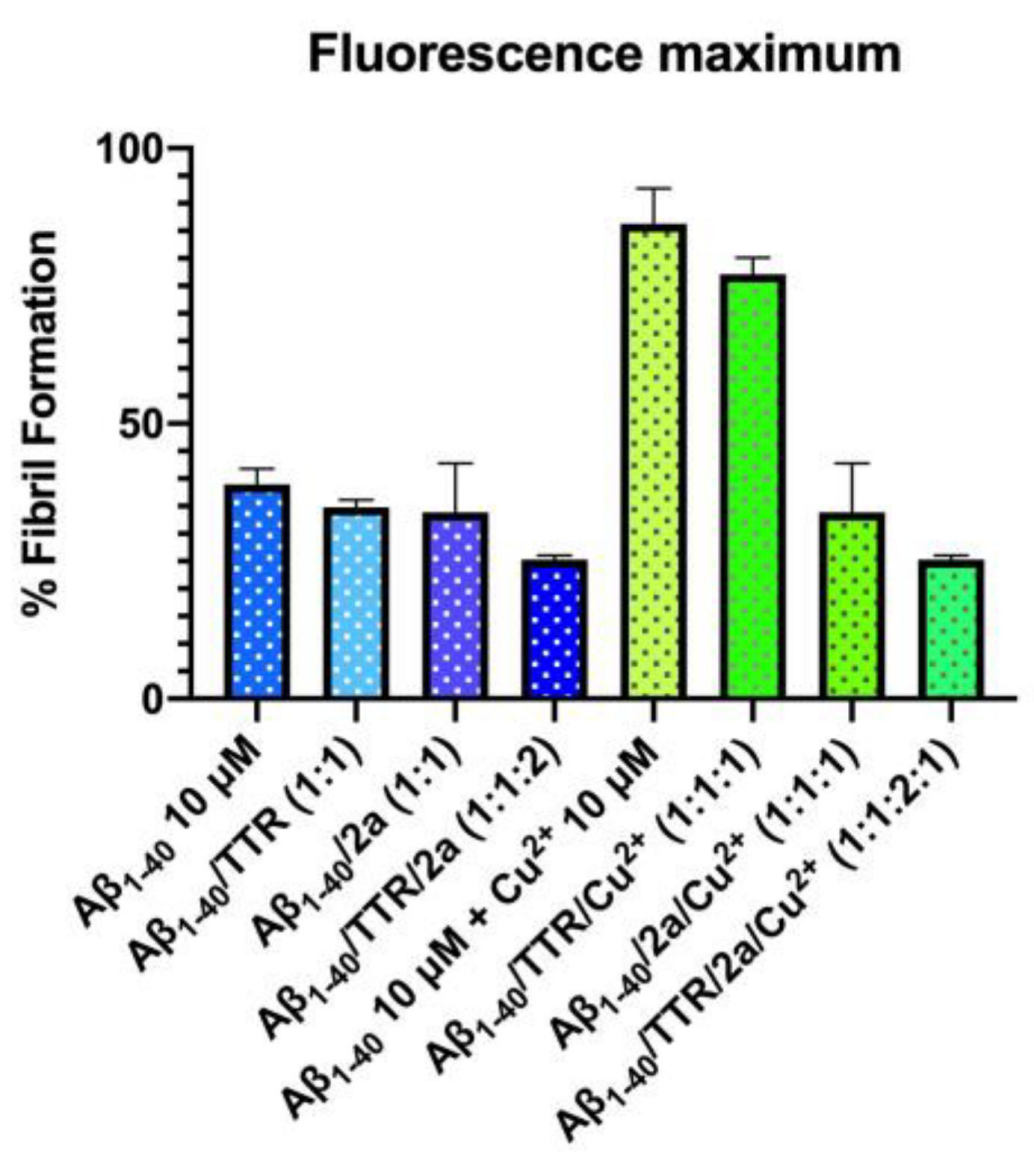
Percentage of Aβ_1-40_ fibril formation assessed by ThT fluorescence intensity in the absence and presence of 10 µM of Cu^2+^, with and without **2a** (1:1 ratio) and with or without TTR (10 µM) pre-incubated or not with **2a** (20 µM). The values are given as averaged relative fluorescence percentages (mean ± SEM) from triplicates (without TTR) or duplicates (with TTR) of at least two independent experiments.

In our conditions, Aβ_1-40_ showed a typical kinetics curve in which the primary and secondary nucleation steps are bypassed and the dominant mechanism responsible for the consumption of monomeric Aβ_1-40_ is the elongation of the preformed fibrils, as previously observed by Chiti et al. [21]. Indeed, the amyloid fibril formation was very rapid and without a detectable lag phase, as expected due to the presence of seed fibrils. Under these conditions and without Cu^2+^, compound **2a** was the best compound, having a discrete inhibitory activity at 1:1 ratio (Figure 7 and Figure S7). Its activity resulted to be increased when it was assessed in the presence of Cu^2+^ where compound **2a** showed a completely inhibition of the fibril formation catalysed by the Cu^2+^ ions, (Figure 7 and Figure S8).

We then evaluated the effect of TTR under the experimental conditions without Cu^2+^ (Aβ_1-40_/TTR, ratio 1:1, Figure 7) and we found a result comparable to the one previously reported by Chiti et al. [21]. In fact, TTR was found not to significantly affect the aggregation kinetics, confirming that Aβ_1-40_ fibril elongation is not influenced by the presence of tetrameric TTR (Figure S9). A slight positive effect was, however, observed when the tetrameric TTR was previously incubated with compound **2a** (1:2 ratio, TTR/**2a**, Figure 7 and Figure S9). The contribution of TTR without ligand was more visible in the presence of Cu^2+^ ions (1:1:1 ratio, Aβ_1-40/_TTR/Cu^2+^, Figure 7 and Figure S10). This effect was even more remarkable when the TTR was pre-incubated with compound **2a** (Figure 7 and Figure S10).

## 5. Discussion

In the context of a multifactorial neurodegenerative disorder such as AD, we report the synthesis and the characterization of new multifunctional compounds **1a**–**c**, **2a**–**c**, **3a**–**c**, **4a**–**c**, **5a**–**c** that can be considered hybrid structures where a natural acid was covalently conjugate with a promising bioactive moiety (2-(trifluoromethyl)benzyl-hydroxylamine hydrochloride) identified in our precedent work [56]. Considering the results obtained for the ferulic acid (FA) hybrids, that showed a good chelating ability, antioxidant capacity and a moderate activity as inhibitor of Aβ aggregation [56], here we chose cinnamic acid, caffeic acid and umbellic acid in order to investigate the effect of the absence or the presence of more than one hydroxyl group in the new hybrids compared to the previous ones [52,53]. In addition, valproic acid and lipoic acid were selected for their known properties to contrast the AD progression [57,58].

Evidence suggests that the dysregulation of biometals in the brain plays a relevant role in the pathogenesis of AD. In fact, the biometals, such as zinc, iron and copper, are essential for neuronal activity, but at the same time, the loss of metal ion homeostasis is associated with neurodegenerative disorder onset [74,75].

Zn^2+^ possesses high positive charge density due to its closed shell d^10^ diamagnetic electron configuration. This characteristic makes Zn^2+^ able to bind nucleic acids and proteins, and it is a co-factor of more than 300 enzymes [76]. In addition to zinc, iron and copper are transition metals belonging to the fourth period. In the body, iron exists in two main oxidation states II (d^6^ configuration) and III (d^5^). Iron forms coordination compounds, especially with ligands containing hydroxide, phenolate and carboxylate. Fe^2+^ prefers nitrogen ligands, such as amines and imidazole, due to lower charge density. Cu^2+^ has a d^9^ electron configuration often characterized by a tetragonal distortion of the coordination geometry due to the Jahn–Teller effect [77]. Cu^2+^ coordinates with molecules containing oxygen and nitrogen atoms such as amines, amides and heteroaromatics (pyridine, pyridazine and imidazole) [78].

**Figure 8:**
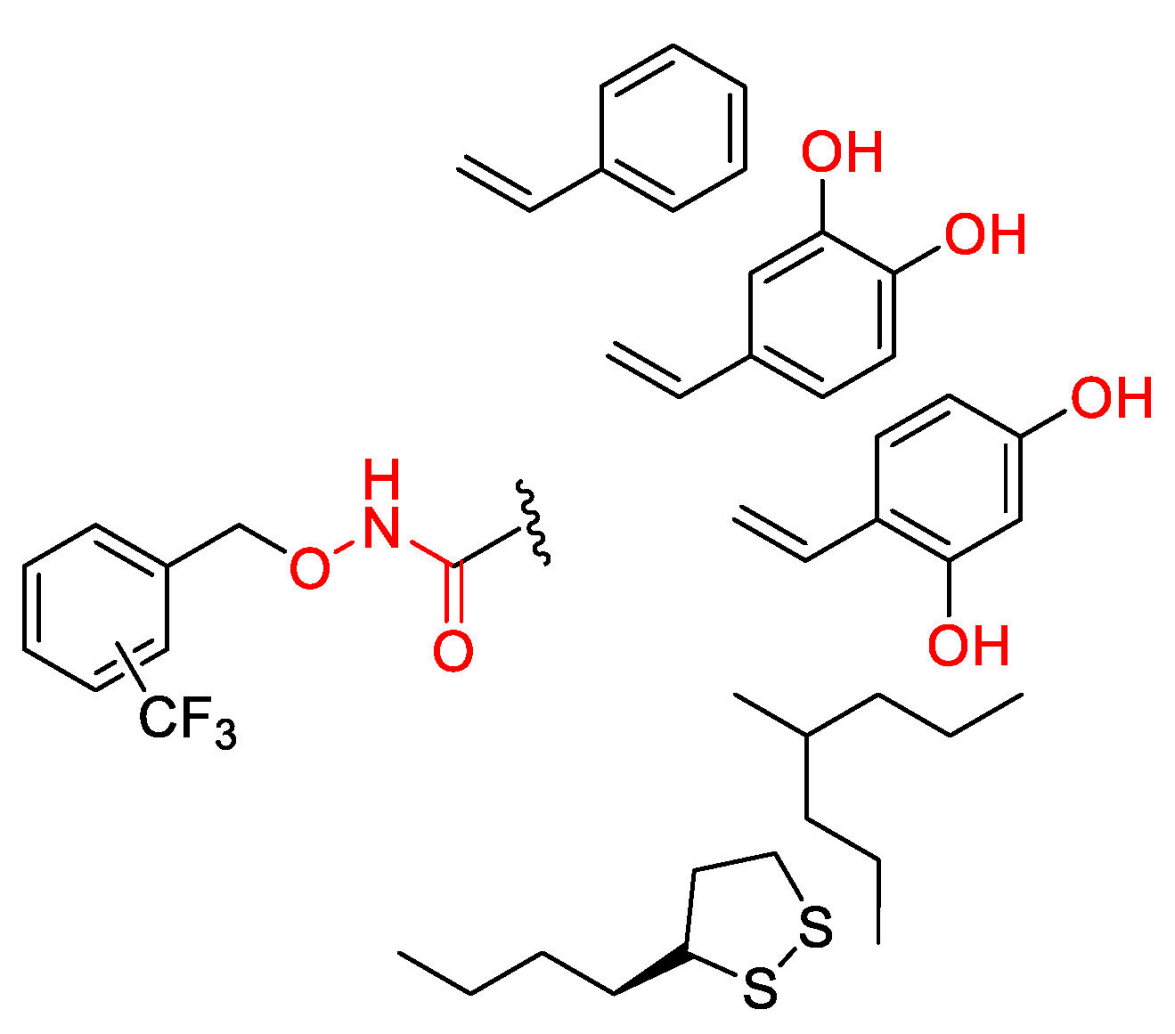
In red the chemical portions of studied compounds that have been proved to chelate metals.

All synthesized derivatives possess a common heteroatomic oxyamidic motive (O–NH–CO-) that potentially can chelate metals, Figure 8. In addition, compounds **2a**–**c**, **3a**–**c** are characterized by a second aromatic portion substituted with hydroxyl groups in catecholic or resorcinol position, commonly known to be able to chelate biometals [2,79], Figure 8.

The ability of the compounds **1a**–**c**, **2a**–**c**, **3a**–**c**, **4a**–**c**, **5a**–**c** to chelate biometals such as Cu^2+^, Fe^2+^ and Zn^2+^ were studied by UV–Vis spectrometer. Studies suggested that phenols substituted in otho- para- or meta- para- positions, potentially can chelate metals as well as molecules which contain the O–NH–CO- motive [78,80]. Unfortunately, despite the presence of different potential Zn^2+^ chelating groups, under the experimental condition applied, none of the hybrids were able to bind Zn^2+^. This highlights that the oxyamidic motive is not appropriate to chelate Zn^2+^, while, regarding the inefficacy of the catechol group, the result agrees with data published where the feeble or the absence of interaction with Zn^2+^ has been attributed to the high instability of catechol [81].

**Figure 9:**
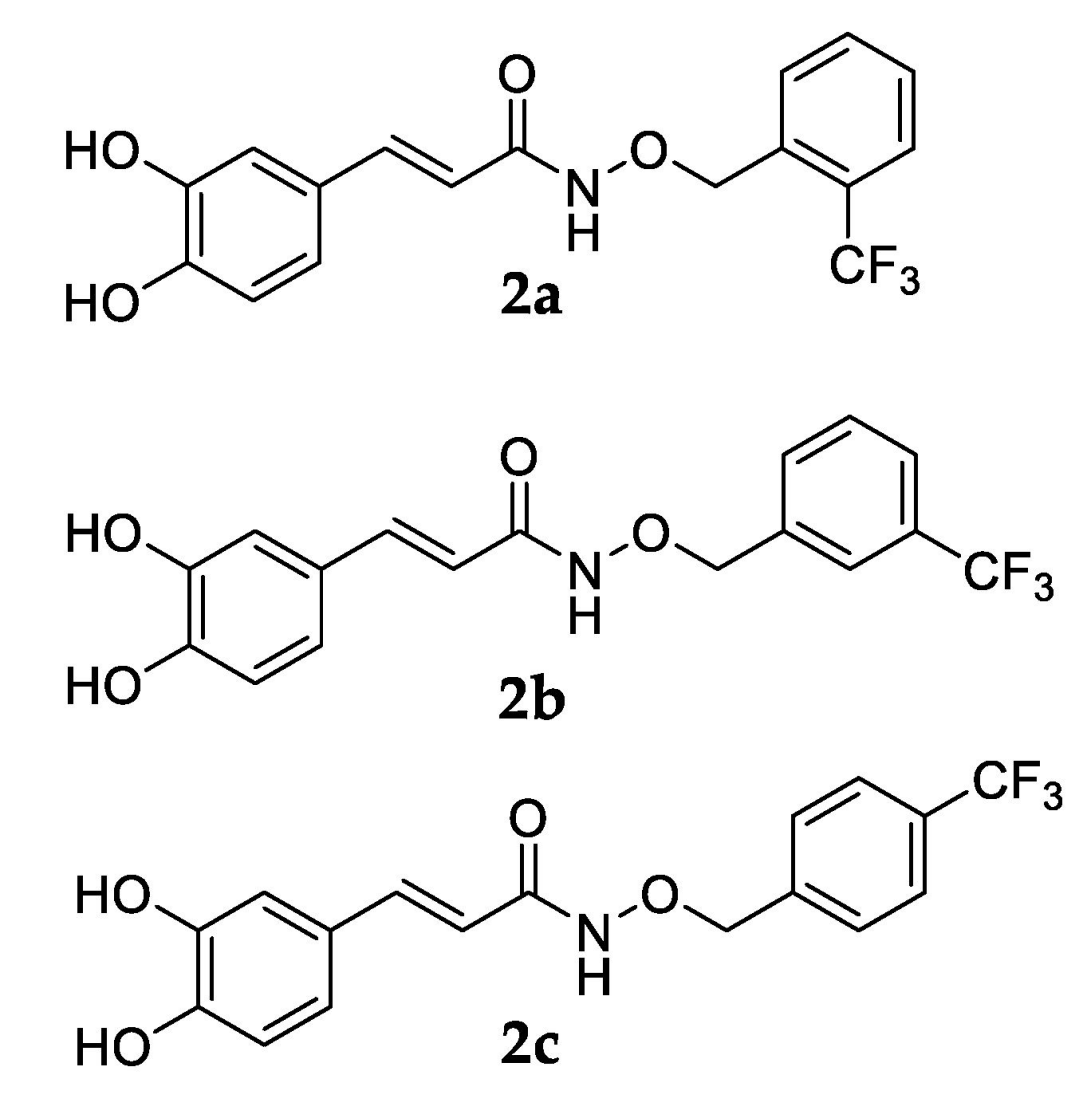
Chemical structures of compounds **2a**–**c**.

According to the data reporting in the literature that clearly attributes the metal chelating ability of polyphenols to the presence of ortho-dihydroxy polyphenols (catechol), derivative **2a** (Figure 9) chelated Fe^2+^ [79].

Due to the similarity among **2a**–**c** (Figure 9), which differ only for the –CF_3_ group position, we can deduce that the catecholic derivatives **2b**–**c** can bind Fe^2+^ in ratio 1:1.

As mentioned before, the chemical features for a good Cu^2+^ chelator are the polyhydroxylated moieties, carbonyl and amidic groups [82]. As expected, due to the presence of the linker, all compounds bind Cu^2+^ and their chelating profiles become better for **2a**–**c**, **3a**–**c** where the additional –OH groups, in catecholic or resorcinol position, increase the stoichiometry of the complex [Ligand–Cu^2+^] from 1:1 to 1:2 [83]. This new series of hybrid compounds maintains the metal chelation properties of the FA hybrids previously studied [56].

There is a strict connection between the dysregulation of biometals and the oxidative stress that they can induce, favoring the neuronal degeneration. Thus, the results achieved by the metal chelation analysis prompted us to screen **1a**–**c**, **2a**–**c**, **3a**–**c**, **4a**–**c**, **5a**–**c** with the DPPH assay [62]. Considering the good %AA obtained, for the catecholic derivatives **2a**–**c** we calculated the EC_50_ values (6.0–8.9 μM) confirming that they act as good antioxidant agents stronger than the parent FA hybrids and the caffeic acid [56,72]. Starting from this result, we selected compounds **2a**–**c** and **3a**–**c** (resorcinol derivatives) in order to investigate their radical scavenger activity also by in vitro ABTS assay and ex vivo rat brain using the TBARS assay [62,65]. In line with what is already published, the resorcinol derivatives showed lower antioxidant power compare to catecholic analogues [84,85]. The data obtained from both tests agreed with that of DPPH ones, confirming that, among the synthesized compounds, catecholic derivatives **2a**–**c** have the best antioxidant profile. This evidence suggests that when the chain motive O–NH–CO- is connected to a catecholic group, the resulting molecules act as strong radical scavenger agents. Moreover, the position of the -CF_3_ (in -ortho, -meta, -para) has a slight effect in the antioxidant power, especially for the ABTS assay. This is probably related to a different conjugation on the aromatic ring. Comparing this result, together with the good Cu^2+^ and Fe^2+^ chelating activity that they showed, catecholic derivatives **2a**–**c** were selected to investigate their effect on the kinetic of Aβ aggregation in presence and in absence of Cu^2+^ and TTR.

Recently, several studies have reported that there is a positive cross-interaction between TTR and Aβ because TTR participates in the Aβ clearance from the brain to the liver [22,38,86]. Even if the mechanism by which TTR acts as neuroprotective protein is still unknown, experimental evidence suggests that the stability of the TTR protein is essential for the positive cross interaction with Aβ [87,88]. One approach to contribute to the TTR stabilization is the use of small molecules able to bind the T_4_-BPs, avoiding the first step of the tetramer dissociation. Due the morphology of the T_4_-BPs, compounds characterized by two aromatic groups connected together and, decorated with acid moieties (-OH, -COOH) and/or halogen atoms, can bind the TTR channel occupying the halogen binding pockets (HPBs) [89]. Looking at the chemical structure of compounds **2a**–**c** that remember the classic motif of a TTR binder, it can be hypothesized that they enter into the T_4_-BPs [90,91,92]. The ANS displacement assay [69] confirmed that the catecholic derivatives **2a**–**c**, at concentration of 100 μM, are able to interact with T_4_-BPs (Figure 6) and in particular **2a** showed the best profile. Thus, it is plausible thinking that the aromatic rings take place into the hydrophobic depression orienting the –CF_3_ group towards the HBPs while the catecholic towards the hydrophilic amino acids.

Considering all the results achieved, compounds **2a**–**c** were selected to assess if beyond their ability to chelate biometals, to act as antioxidants and to bind TTR they were also capable to reduce the fibril formation of Aβ_1-40_ peptide. We decided to work with Aβ_1-40_ because in a previous study it had been observed that Cu^2+^ accentuated distinct misfolding of Aβ_1-40_ and Aβ_1–42_ peptides, with an acceleration of fibril formation of Aβ_1-40_ in the presence of Cu^2+^ which was still visible in the ThT test [73]. Thus, we designed a ThT fluorescence spectroscopy assay which simultaneously allowed to study the effect of the compounds on the fibrillization process of Aβ_1-40_ in the presence or absence of Cu^2+^ ions. Moreover, applying the same protocol we can also evaluate if the stabilization of TTR tetramer structure, through the interaction of a small molecule with the hydrophobic pockets, can improve the positive cross-interaction between TTR and Aβ_1-40_; always in the presence or absence of Cu^2+^ ions.

As previous mentioned, among the catecholic derivatives, compound **2a** showed the best inhibitory profile on Aβ_1-40_ fibrillization process in the absence of Cu^2+^. Moreover, a more pronounced effect was observed when the fibril formation was catalysed by the Cu^2+^ ions confirming the strong chelating property of **2a**. When the tetrameric TTR was previously incubated with compound **2a** (1:2 ratio, TTR/**2a**), a slight positive effect was observed, suggesting that the interaction between **2a** and TTR tetramer can improve its activity on the Aβ_1-40_ fibril elongation process (Figure 7 and Figure S9). This result is in agreement with data reported in literature [87,93,94].

Under high metal ion conditions, TTR, in absence of ligand, seems to increase its ability to affect the elongation process of Aβ_1-40_, thus confirming its higher affinity for Aβ_1-40_ peptide in the presence of Cu^2+^ [23] (Figure 7 and Figure S10). In addition, this effect is higher when TTR is pre-incubated with **2a** before being added to the solution containing Aβ_1-40_ and Cu^2+^ (Aβ_1-40_/TTR/**2a**/Cu^2+^, 1:1:2:1 ratio). The last result suggests that a combination of two different effects, such as metal chelation and TTR tetramer stabilization, has a great impact on the inhibition of Aβ_1-40_ fibril formation, especially in conditions with high metal ion concentration.

## 6. Conclusions

Due to the multifactorial character of AD, here we propose a series of hybrid compound natural products based as multifunctional agents. Among the newly synthesized compounds, catecholic derivatives **2a**–**c** showed a promising profile. They are good chelators of Cu^2+^ and Fe^2+^, two of the main biometals involved in neurodegeneration, as well as great antioxidants. Compound **2a** was found to be the best multifunctional molecule. In fact, in addition to being a good biometals chelator and a potent antioxidant agent, it is also capable of interacting with the TTR-binding pockets, strengthening the positive cross-interaction between TTR and Aβ_1-40_, especially in the presence of Cu^2+^.

## Figures and Tables

**Table 1:** Half maximal effective concentration EC_50_, Trolox equivalent antioxidant capacity (TEAC).

Compounds	EC_50_ µM ^a^ DPPH	TEAC µM ^a^
**2a**	8.9 ± 0.2	12.4 ± 0.4
**2b**	6.1 ± 0.1	10.9 ± 0.3
**2c**	6.0 ± 0.1	10.4 ± 0.4

^a^ Data are presented as the mean ± SEM of three independent experiments.

**Table 2:** ABTS assay: EC_50_ of compounds **2a–c** and its expression in TEAC.

Compounds	EC_50_ µM ^a^ ABTS	TEAC µM ^a^
**2a**	9.9 ± 0.2	11.3 ± 0.1
**2b**	13.4 ± 0.1	11.7 ± 0.01
**2c**	22.9 ± 0.5	11.8 ± 0.02

^a^ Data are presented as the mean ± SEM of three independent experiments.

## Data Availability

Not applicable.

## Supplementary Materials

The following supporting information can be downloaded at: https://www.mdpi.com/article/10.3390/pharmaceutics15102369/s1, Spectra S1–S45: ^1^H-NMR, ^13^C-NMR and mass spectra of compounds **1a**–**c**, **2a**–**c**, **3a**–**c**, **4a**–**c**, **5a**–**c**; Figure S1: Chemical structure of all synthesized compounds. Figure S2: UV Spectra of MeOH, compound **4a** and **5a**; Figure S3: Colorimetric metal chelation study of compounds **4a** and **5a**. **A** Derivatives **4a** and **5a** in presence of PV and Cu^2+^; **B** Derivatives **4a** and **5a** in presence of Ferrozine and Fe^2+^; Figure S4: UV Spectra of compound **2c** with increased concentration of Cu^2+^ and molar fraction of the complex; Figure S5: UV Spectra of compound **2c** with increased concentration of Cu^2+^ and molar fraction of the complex; Figure S6: Aggregation kinetics curves of Aβ_1-40_ (10 µM) in the absence (blue) or presence (grey) of 10 µM of Cu^2+^; Figure S7: Aggregation kinetics curves of Aβ_1-40_ (10 µM) in the absence (blue) or presence (orange, grey and yellow, respectively) of compounds **2a–c** (10 µM); Figure S8: Aggregation kinetics curves of Aβ_1-40_ (10 µM) with 10 µM of Cu^2+^ in the absence (blue) or presence (orange) of compound **2a** (10 µM); Figure S9: Aggregation kinetics curves of Aβ_1-40_ (10 µM) in the presence (orange) or absence (blue) of 10 µM of TTR with (grey) or without (orange) the pre-incubation with compound **2a** (20 µM); Figure S10: Aggregation kinetics curves of Aβ_1-40_ (10 µM) and 10 µM of Cu^2+^ in the presence (orange) or absence (blue) of 10 µM of TTR with (orange) or without (grey) the pre-incubation with compound **2a** (20 µM).

## Abbreviations

AD	Alzheimer’s disease
ANS	8-anilino-1-naphthalenesulfonic acid
Aβ	amyloid β
CSF	cerebrospinal fluid
DPPH	2,2-Diphenyl-1-Picrylhydrazyl
FA	ferulic acid
MDA	malondialdehyde
OS	oxidative stress
ROS	reactive oxygen species
SEM	standard error of the mean
T_4_	thyroxine
T_4_-BPs	thyroxine binding pockets
TBARS	Thiobarbituric Acid Reagent Substance
TEAC	Trolox equivalent antioxidant capacity
ThT	Thioflavin T
TLC	Thin Layer Chromatography
TTR	Transthyretin
wt-TTR	wild type TTR
