# Supplementary material for: New Hybrid Compounds Incorporating Natural Products as Multifunctional Agents against Alzheimer’s Disease
Source: Pharmaceutics. 2023 Sep 22;15(10):2369. doi: 10.3390/pharmaceutics15102369 (PMC10610016; doi:10.3390/pharmaceutics15102369)
Supplement: Supplementary file 1 [file pharmaceutics-15-02369-s001.zip › pharmaceutics-2557681-supplementary.pdf]

Spectrum S1: ^{1}H-NMR spectrum of compound **1a**

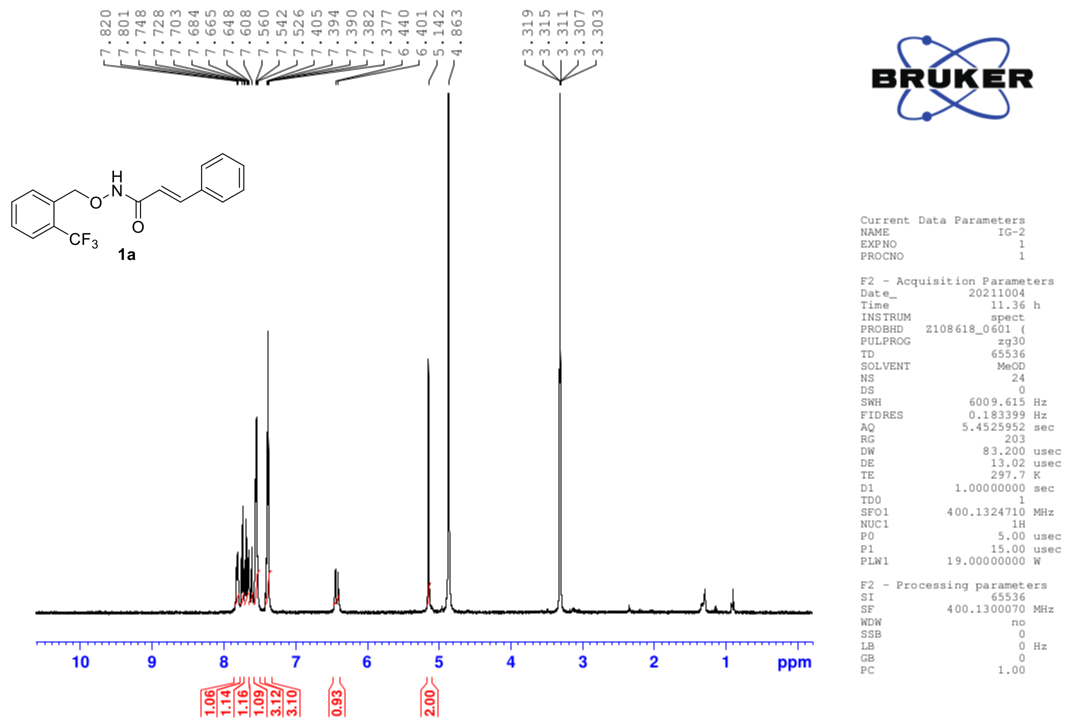

Spectrum S2: ^{13}C-NMR spectrum of compound **1a**

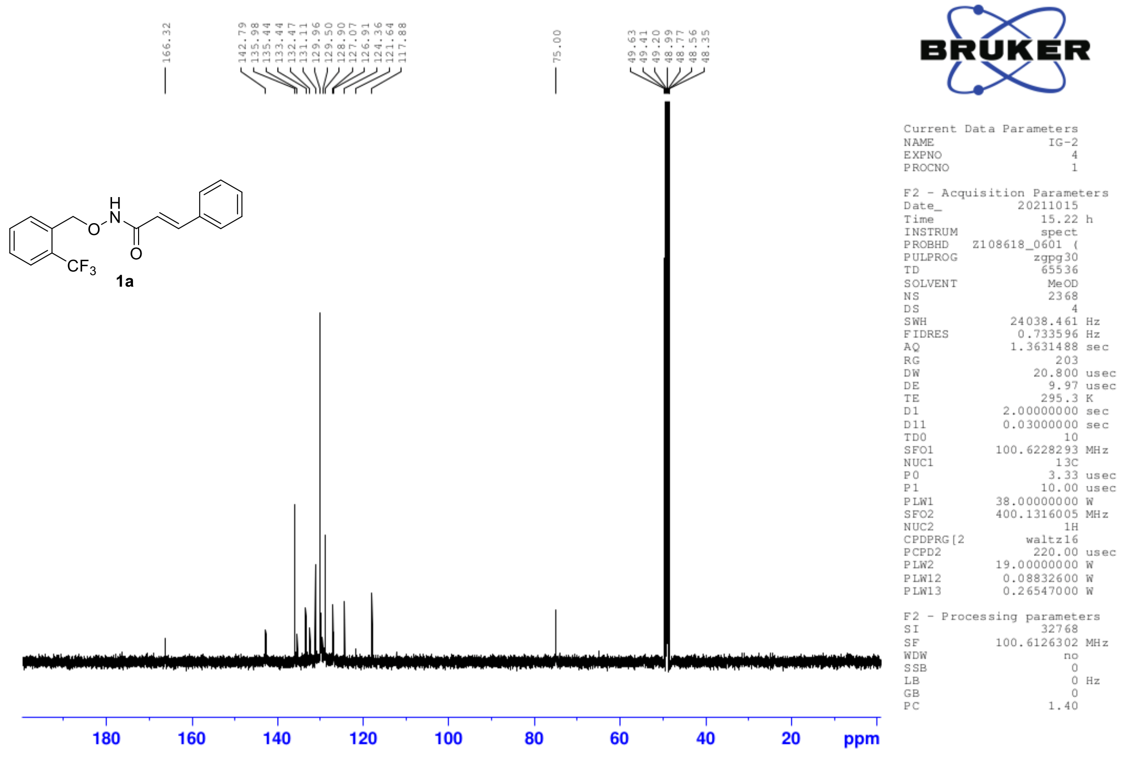

Spectrum S3: Mass spectrum of compound **1a**

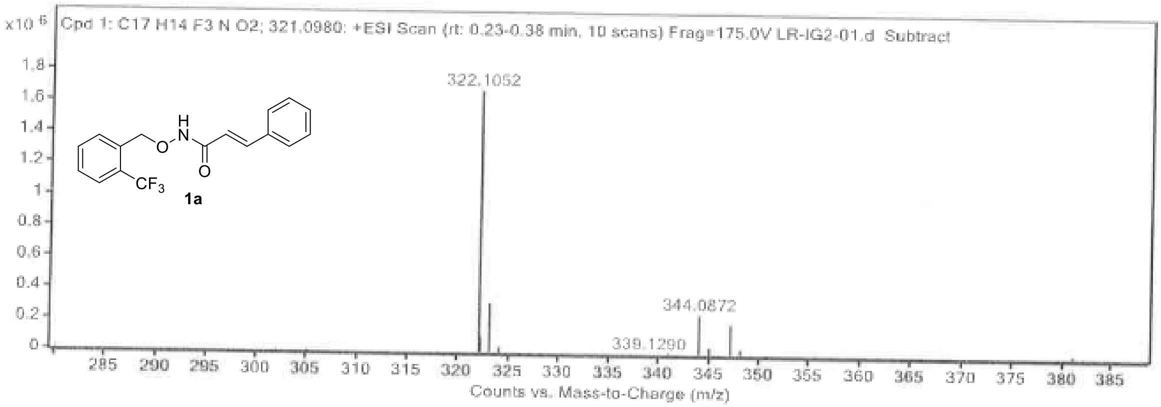

Spectrum S4: ^{1}H-NMR spectrum of compound **1b**

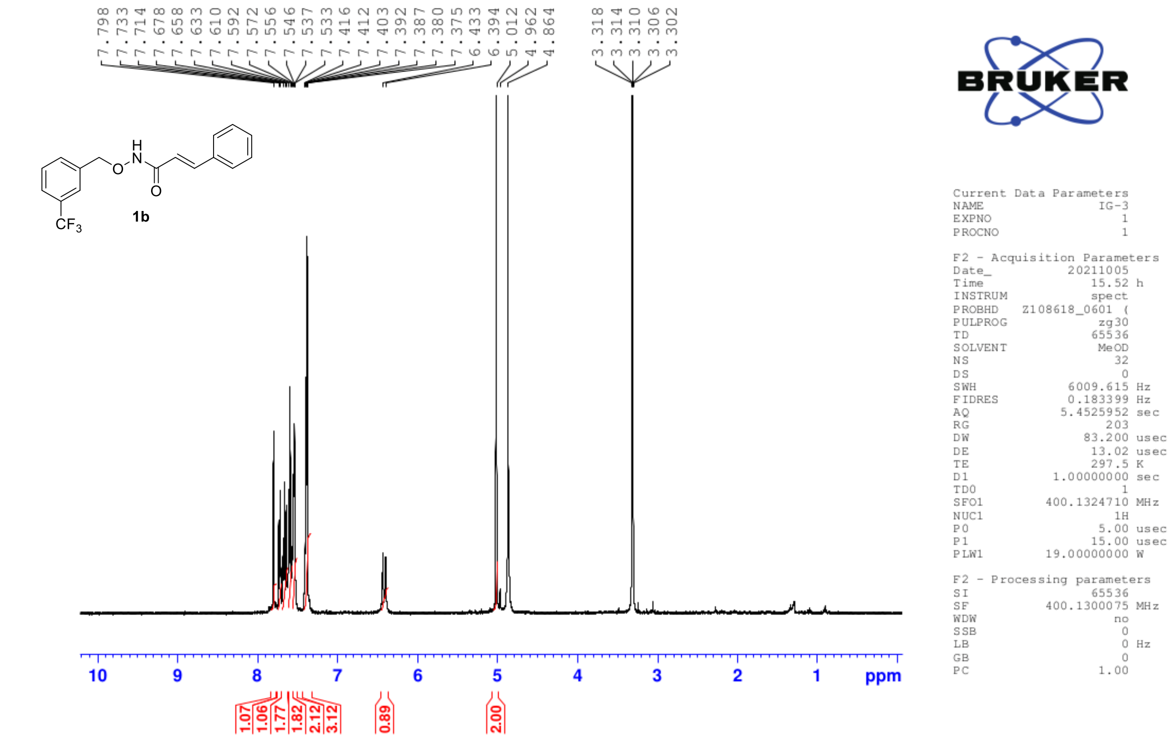

Spectrum S5: ^{13}C-NMR spectrum of compound **1b**

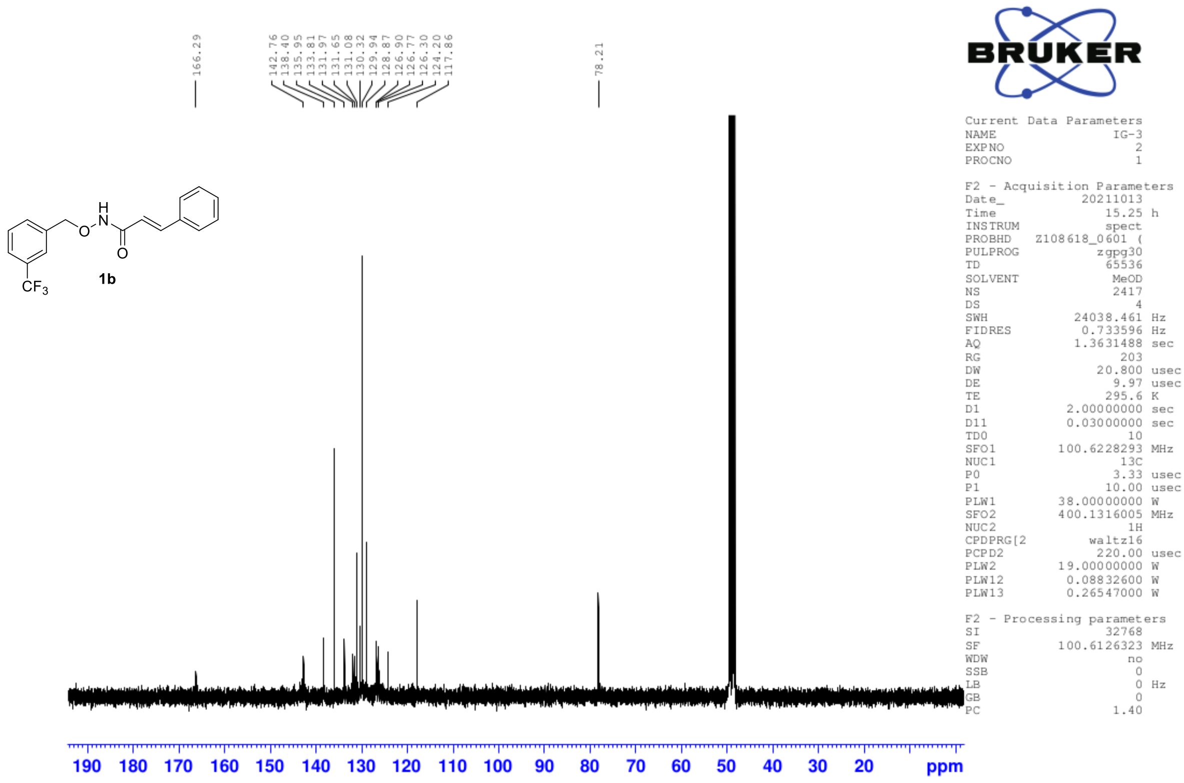

Spectrum S6: Mass spectrum of compound **1b**

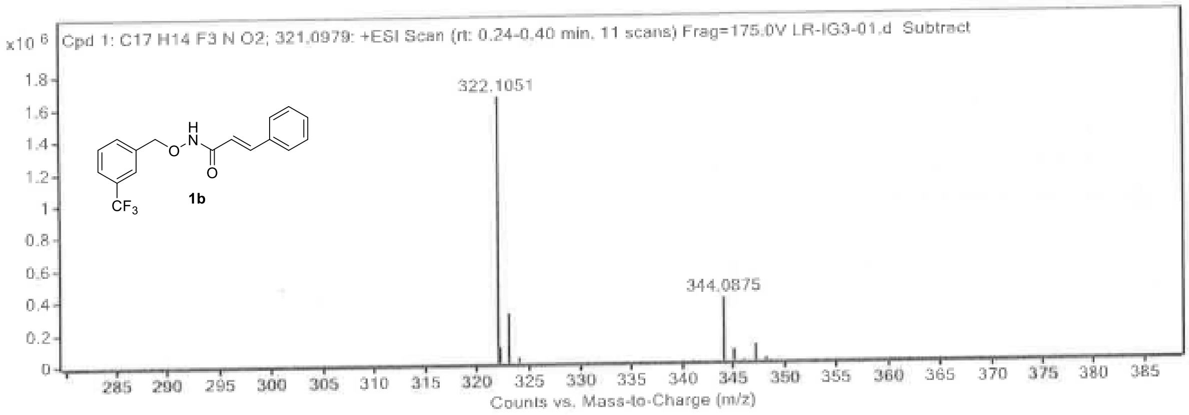

Spectrum S7: ^{1}H-NMR spectrum of compound **1c**

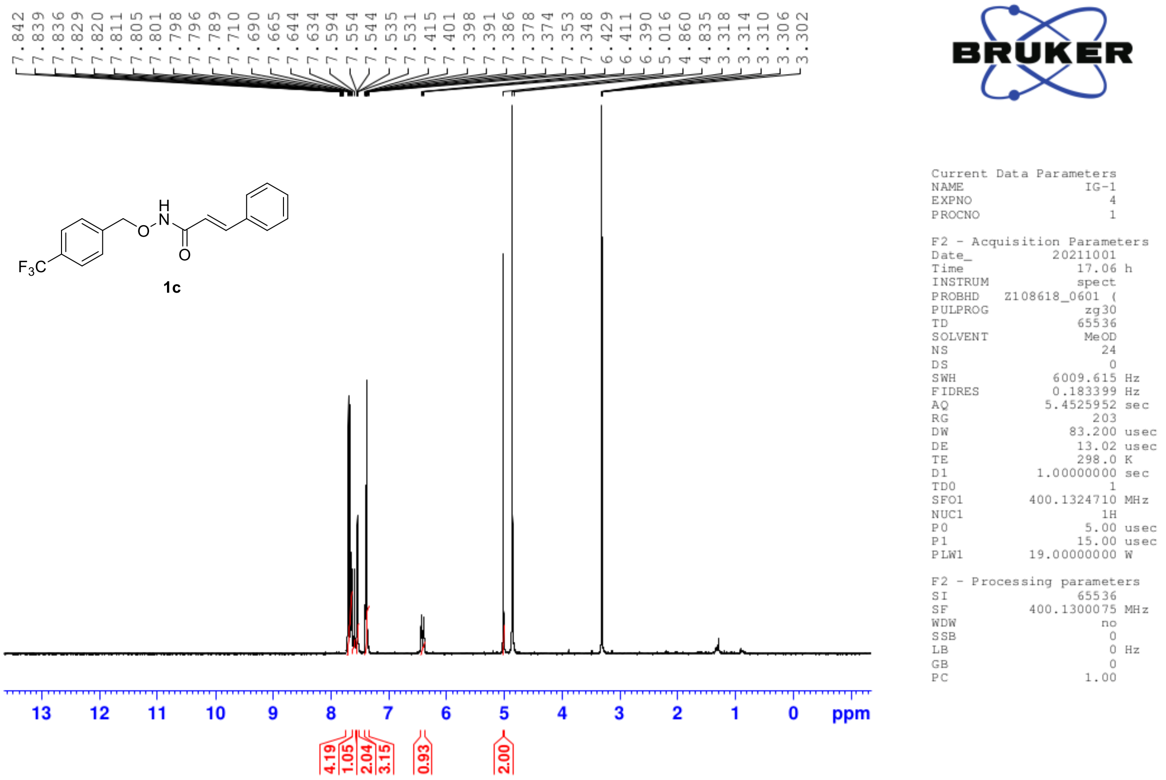

Spectrum S8: ^{13}C-NMR spectrum of compound **1c**

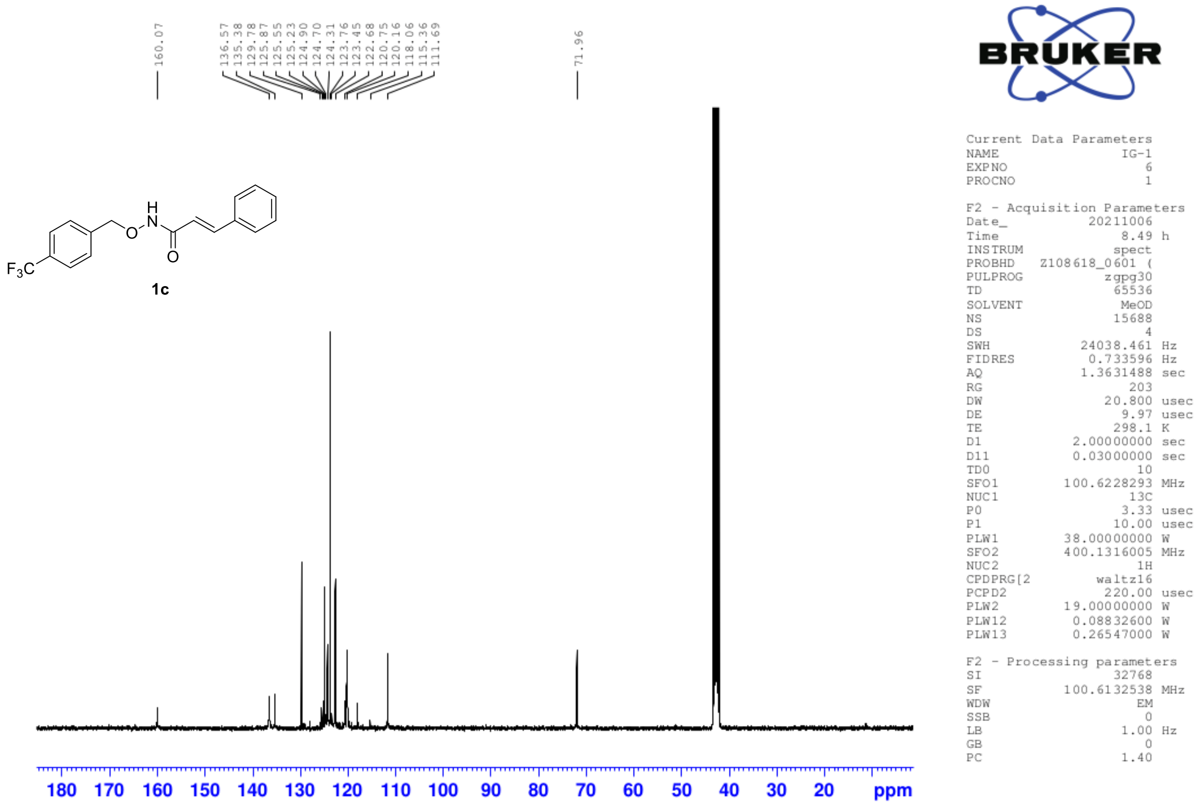

Spectrum S9: Mass spectrum of compound **1c**

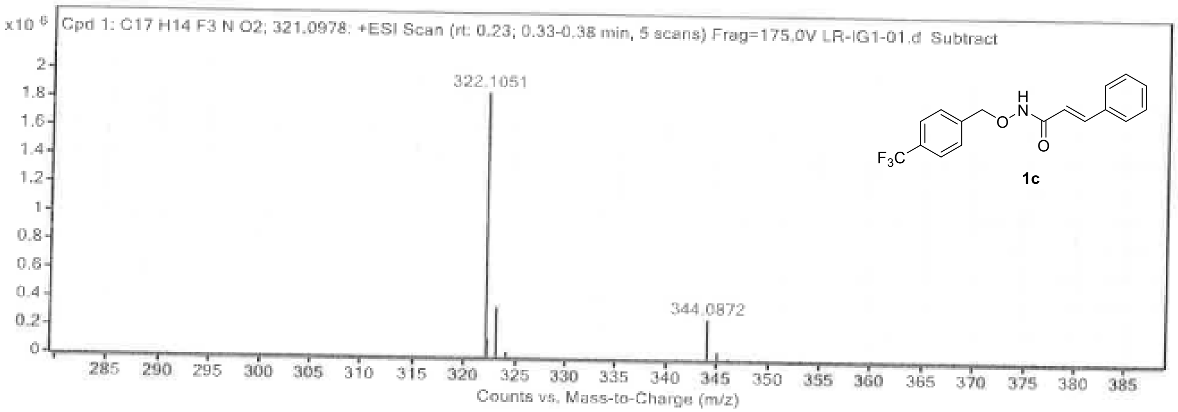

Spectrum S10: ^{1}H-NMR spectrum of compound **2a**

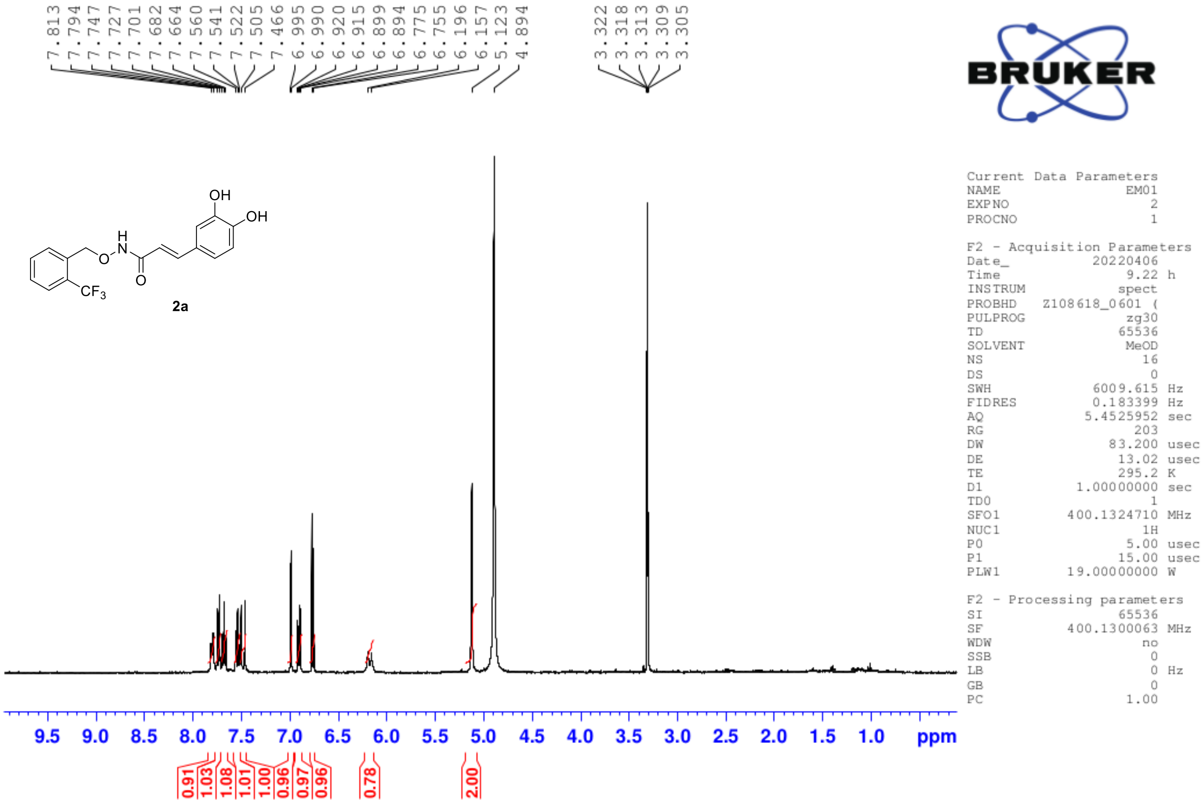

Spectrum S11: ^{13}C-NMR spectrum of compound **2a**

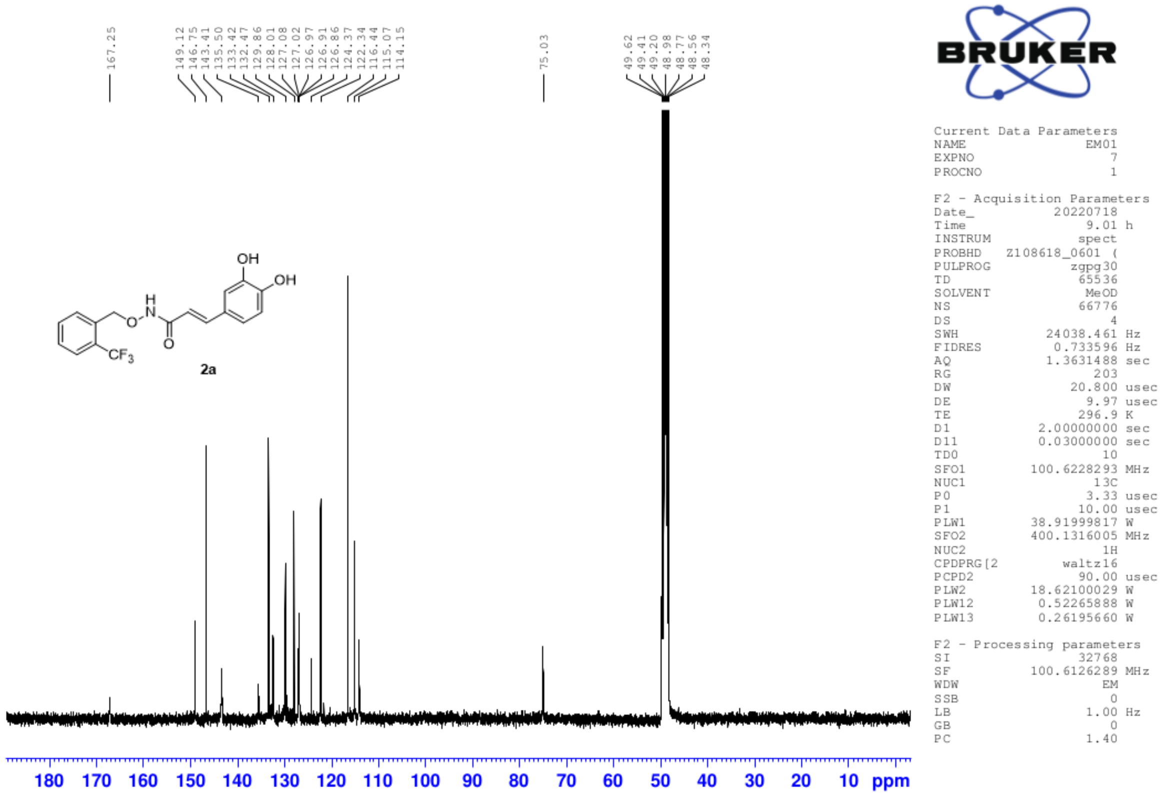

Spectrum S12: Mass spectrum of compound **2a**

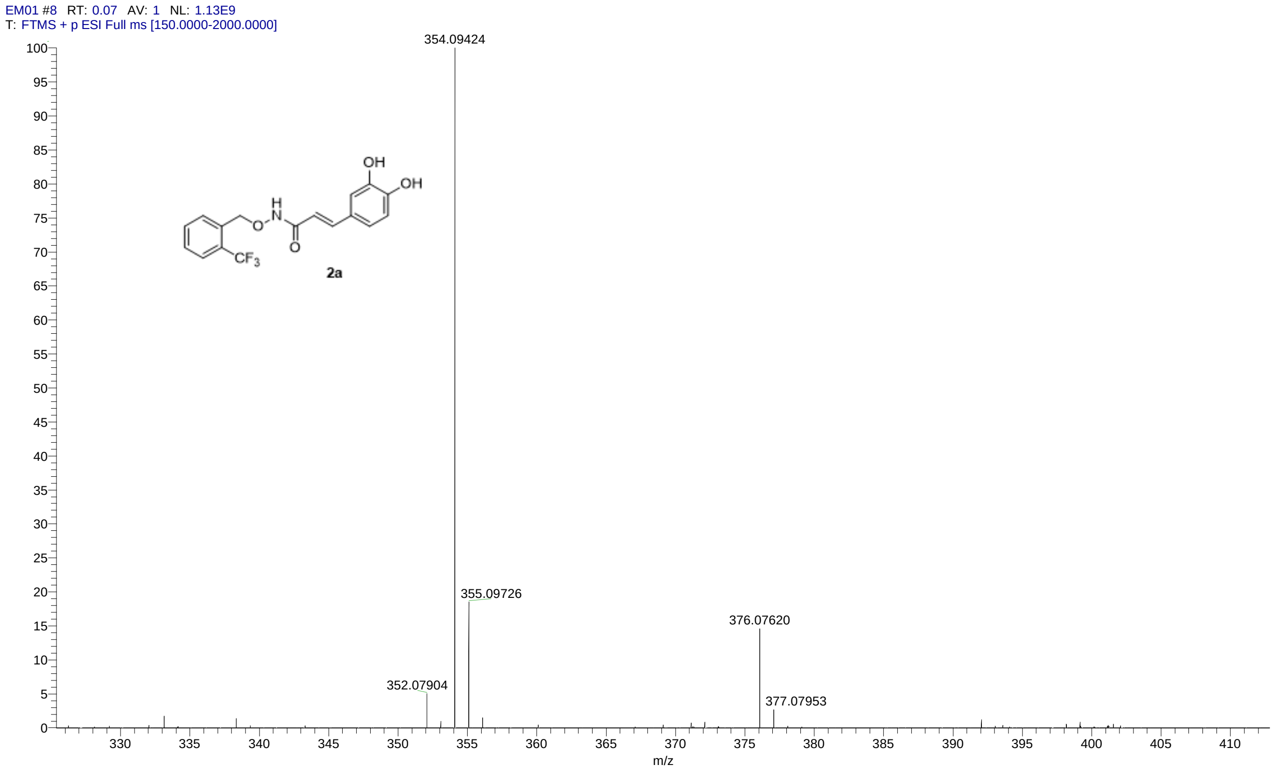

Spectrum S13: ^{1}H-NMR spectrum of compound **2b**

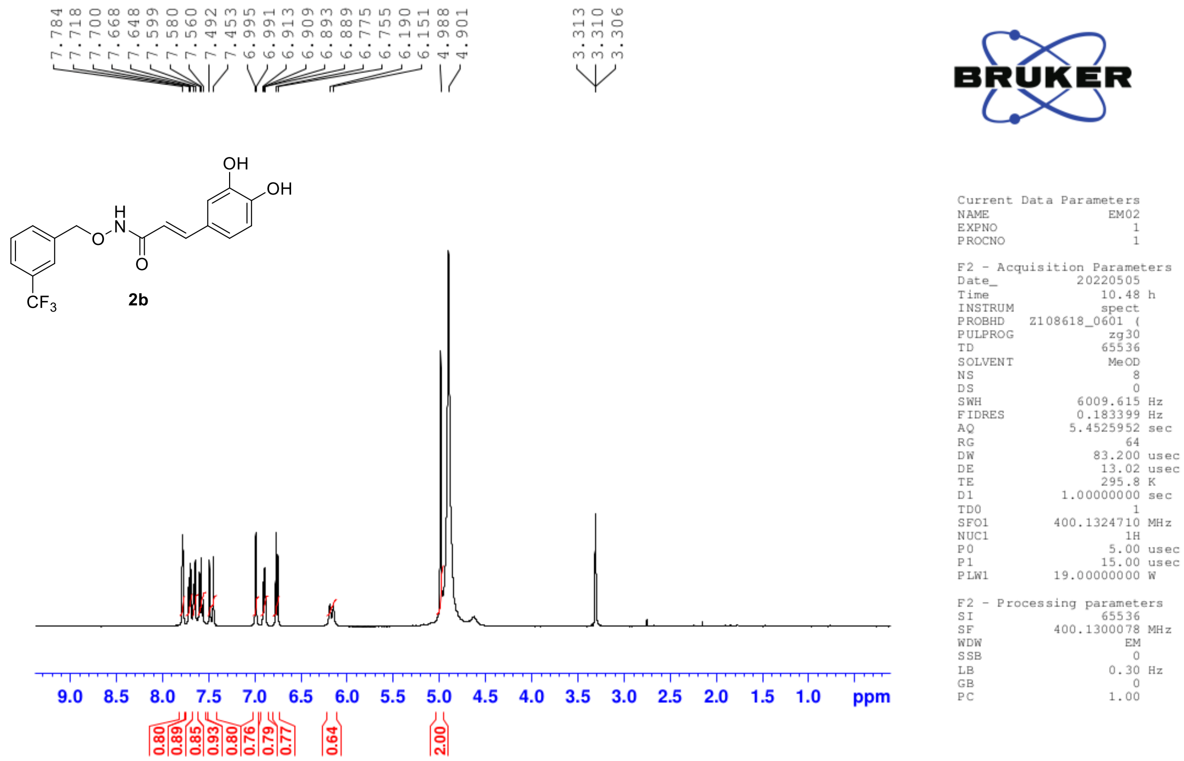

Spectrum S14: ^{13}C-NMR spectrum of compound **2b**

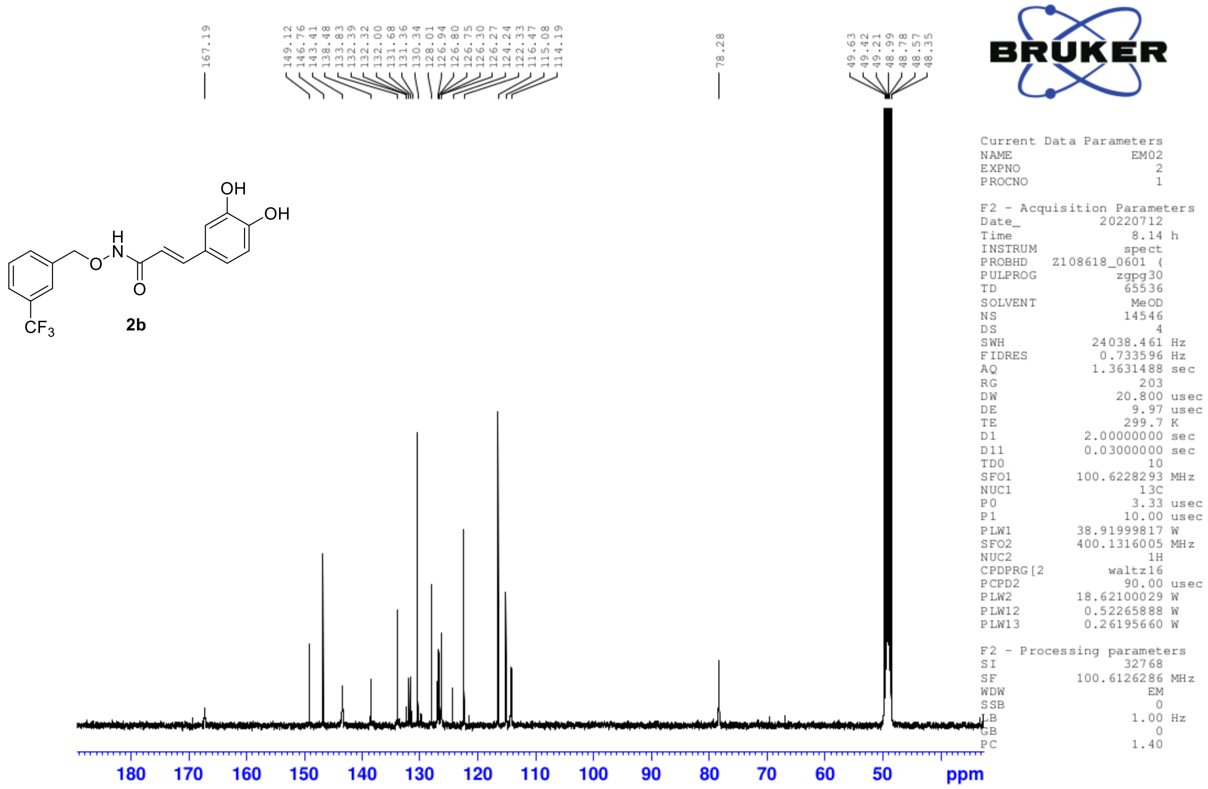

Spectrum S15: Mass spectrum of compound **2b**

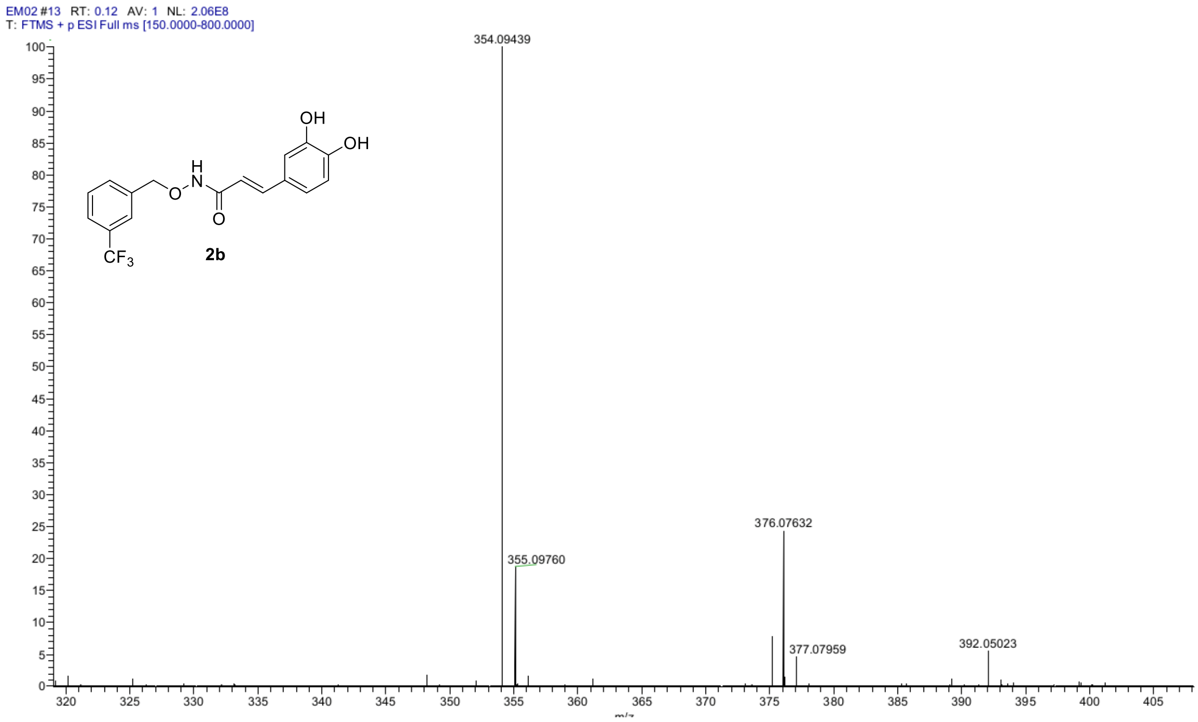

Spectrum S16: ^{1}H-NMR spectrum of compound **2c**

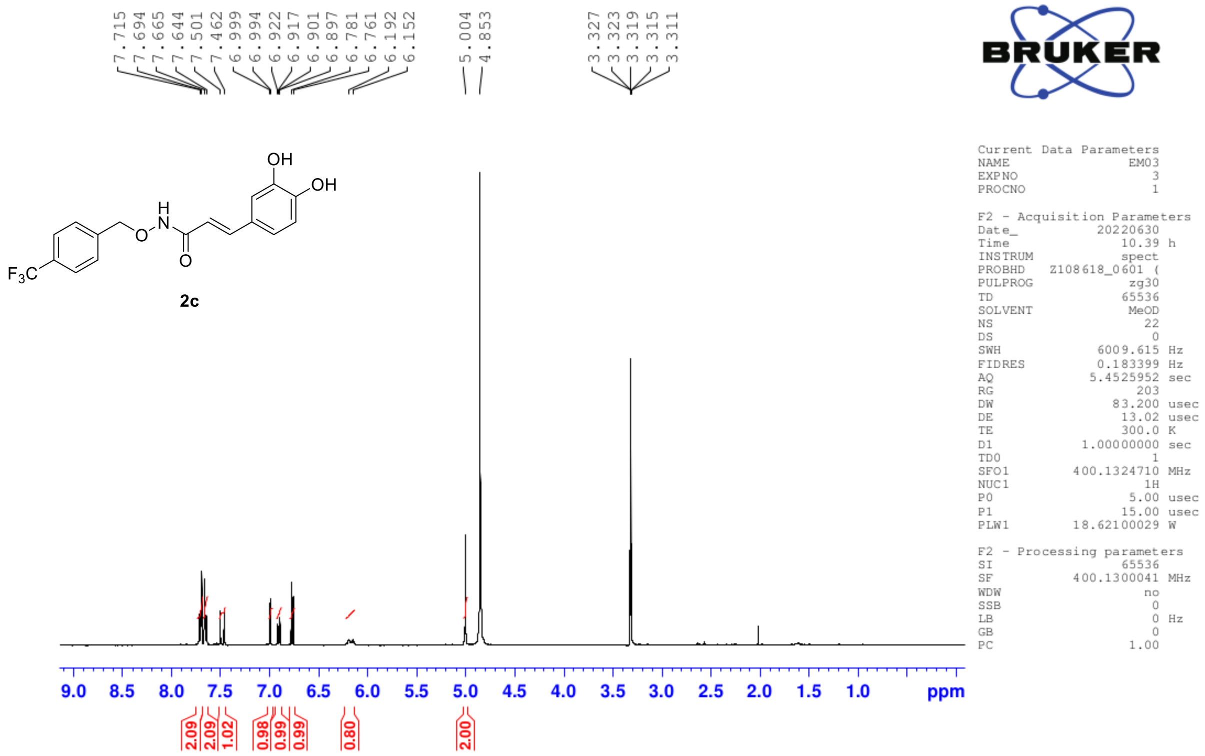

Spectrum S17: ^{13}C-NMR spectrum of compound **2c**

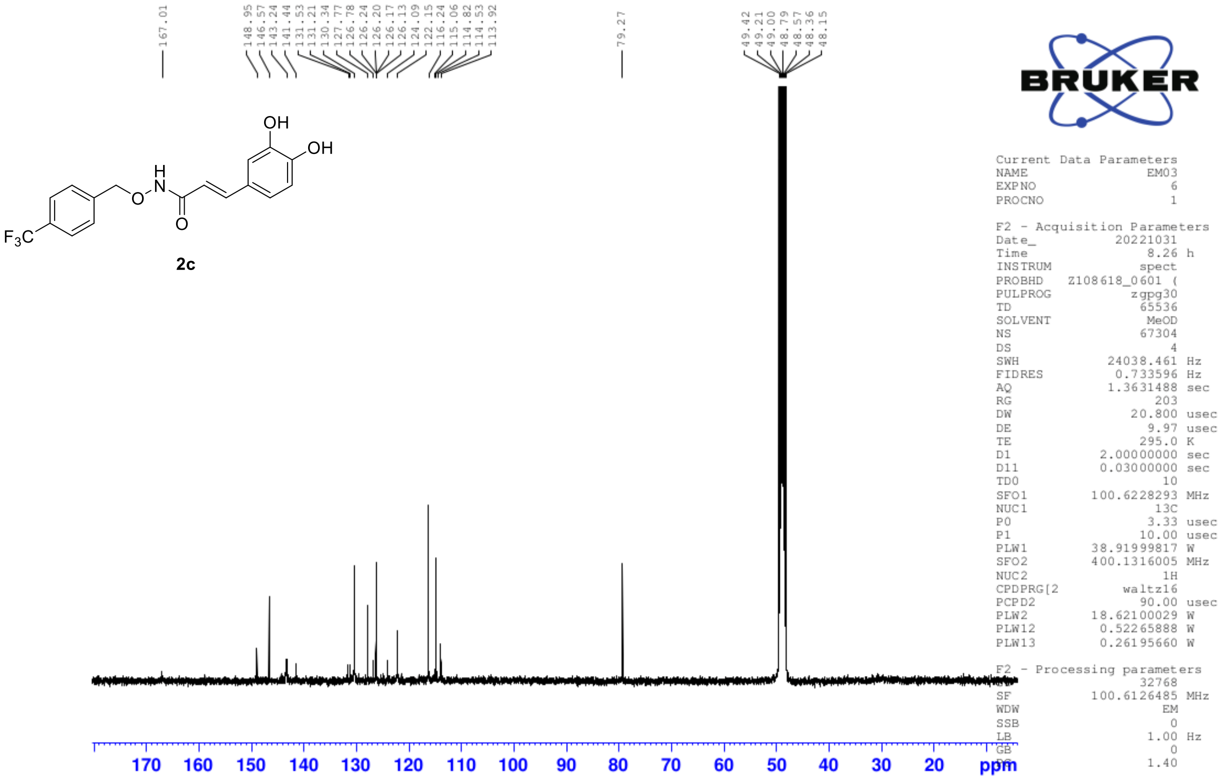

Spectrum S18: Mass spectrum of compound **2c**

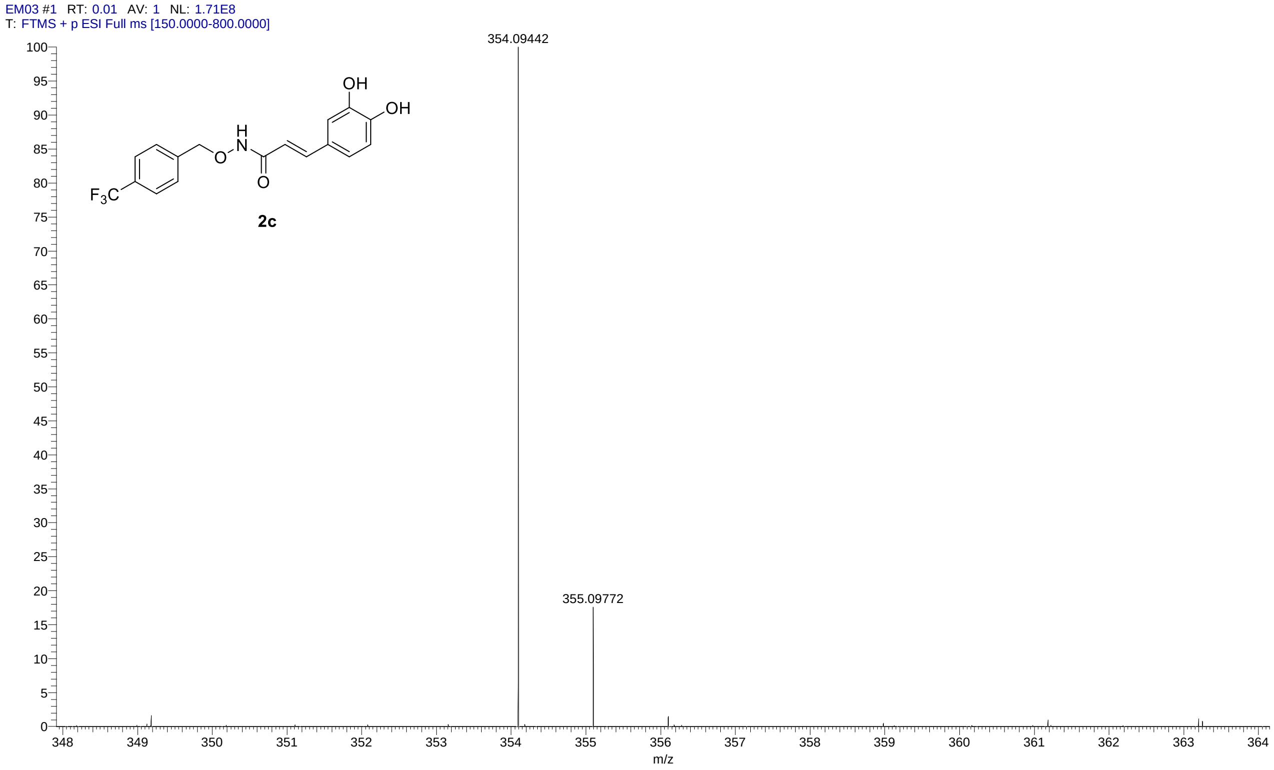

Spectrum S19: ^{1}H-NMR spectrum of compound **3a**

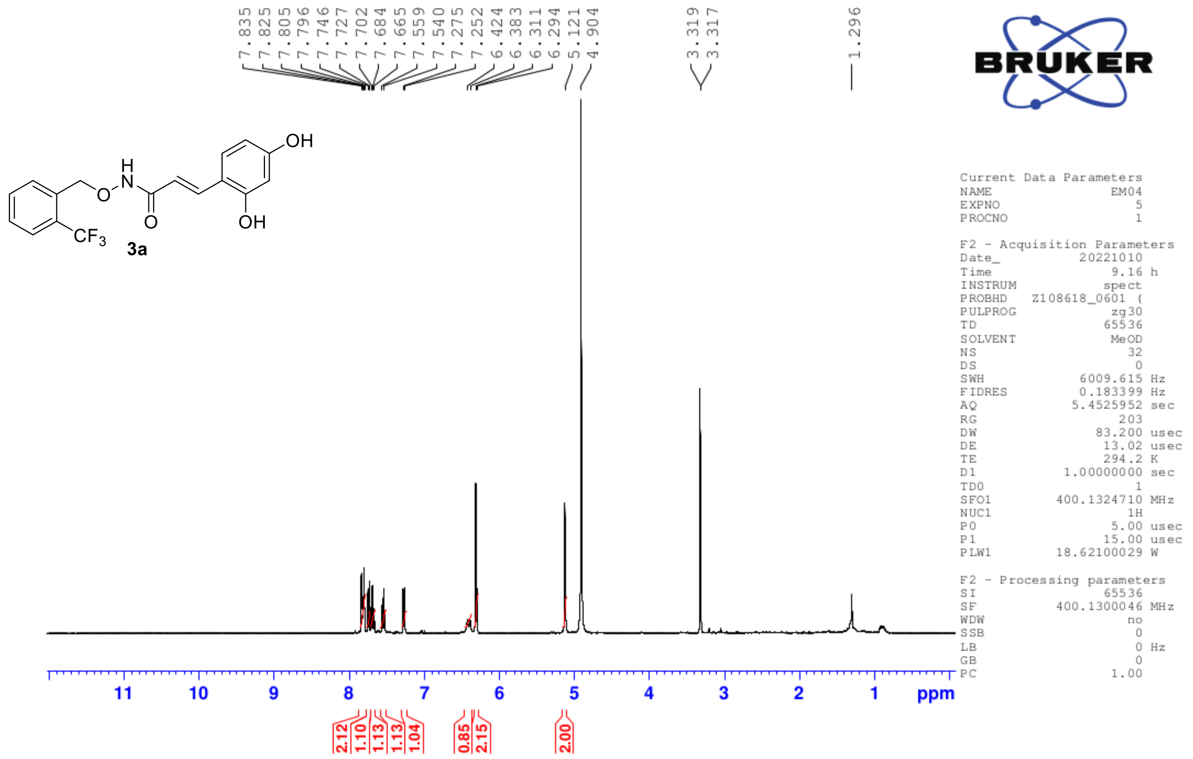

Spectrum S20: ^{13}C-NMR spectrum of compound **3a**

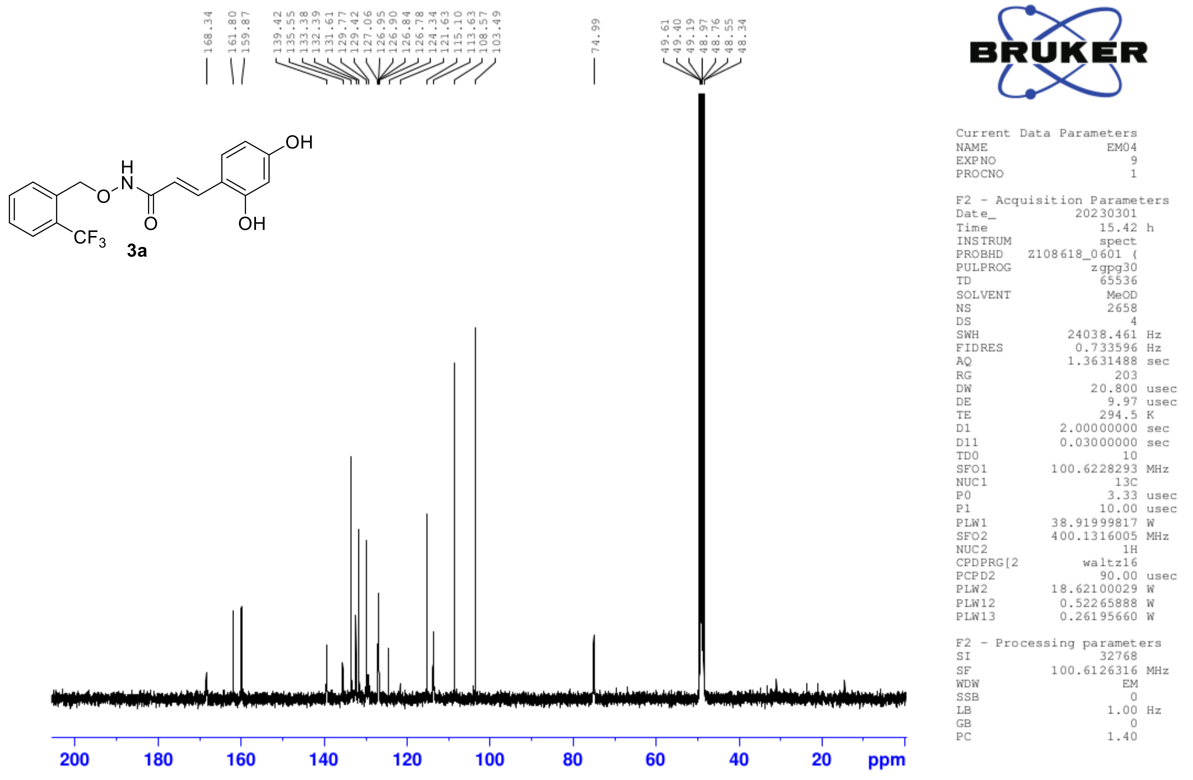

Spectrum S21: Mass spectrum of compound **3a**

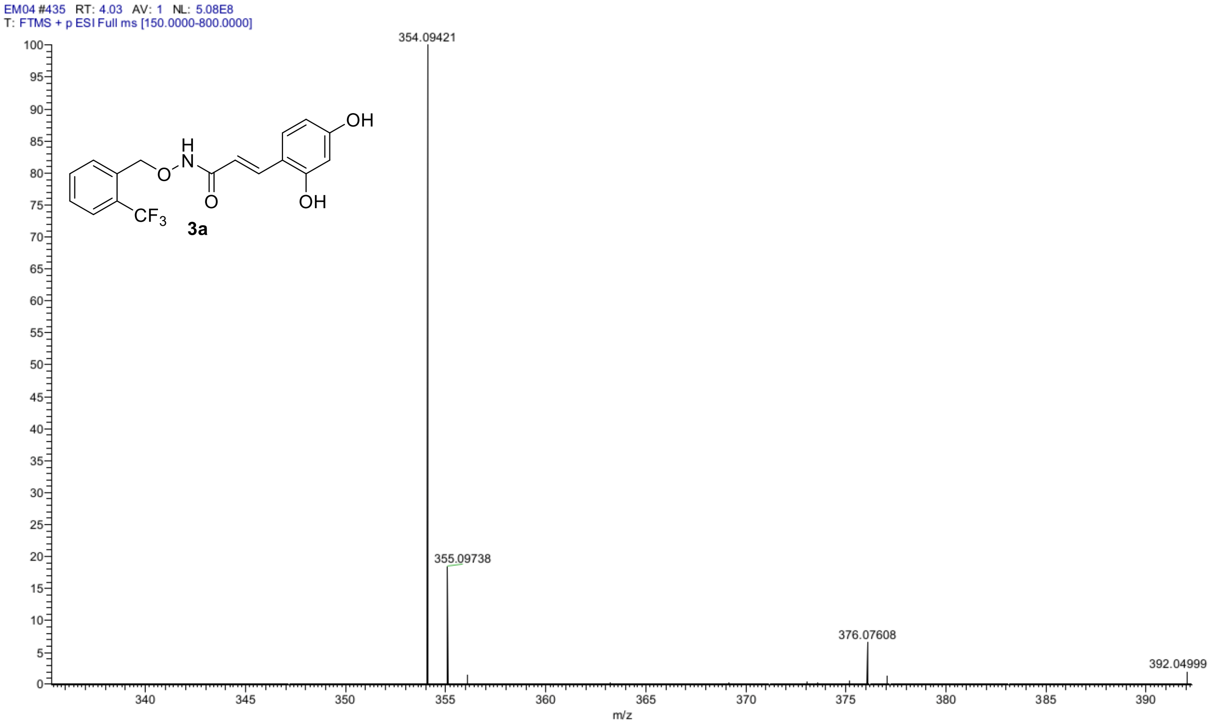

Spectrum S22: ^{1}H-NMR spectrum of compound **3b**

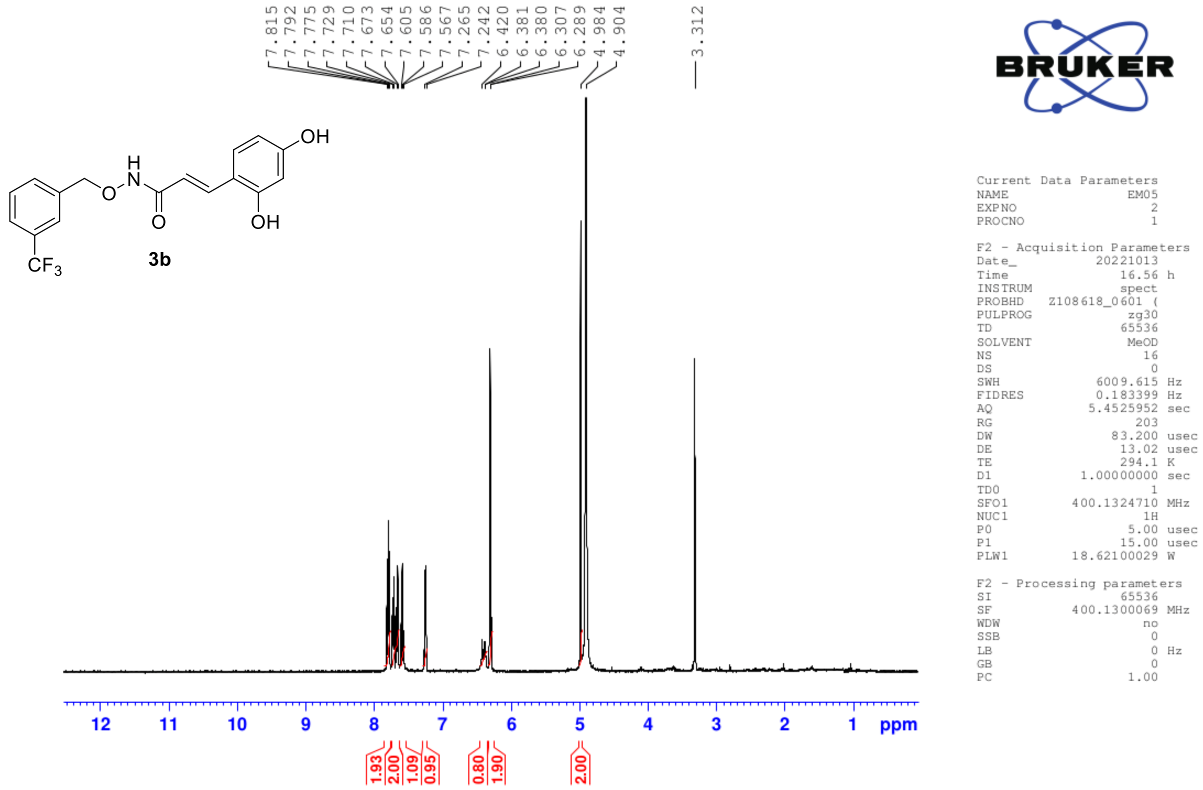

Spectrum S23: ^{13}C-NMR spectrum of compound **3b**

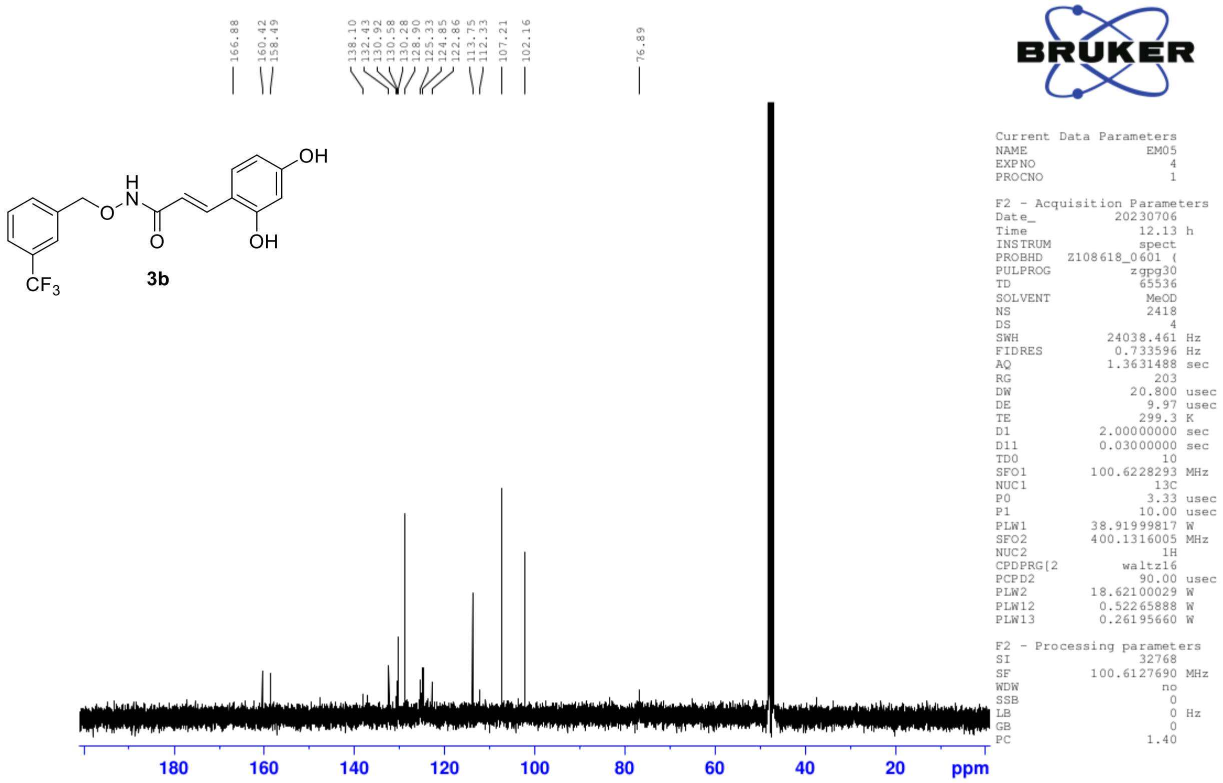

Spectrum S24: Mass spectrum of compound **3b**

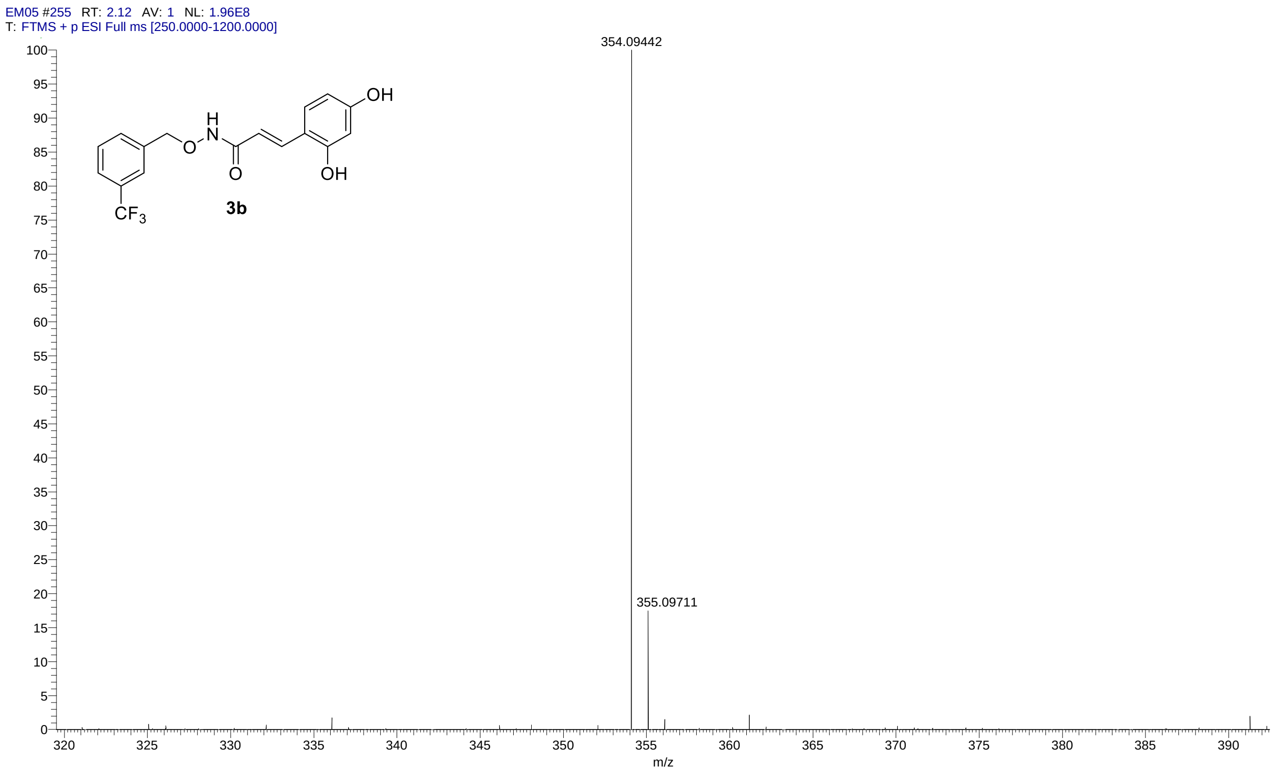

Spectrum S25: ^{1}H-NMR spectrum of compound **3c**

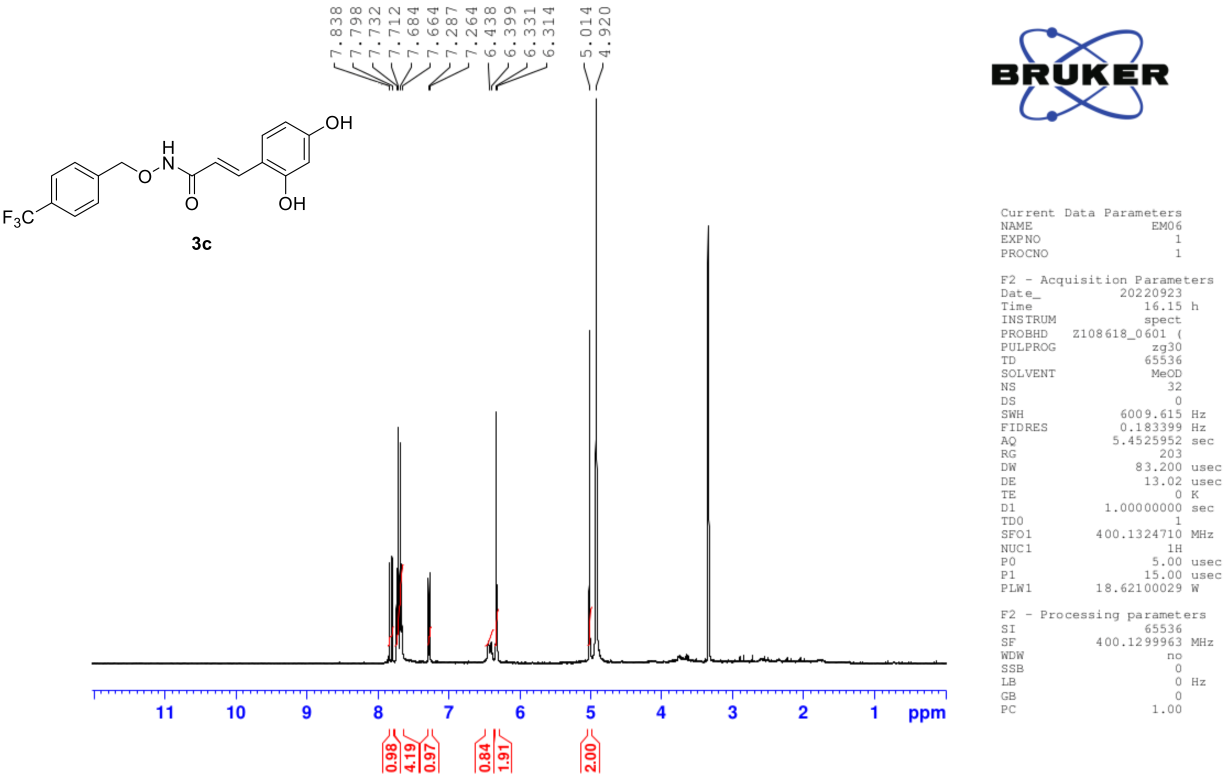

Spectrum S26: ^{13}C-NMR spectrum of compound **3c**

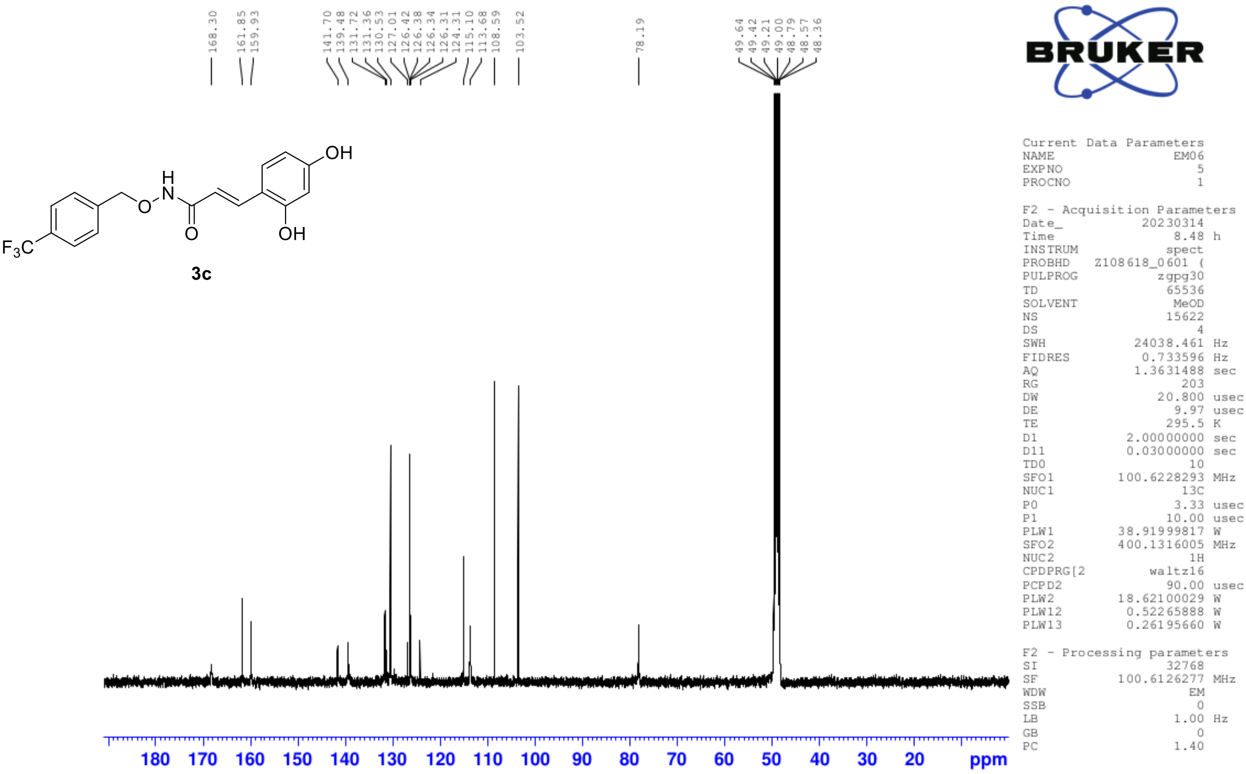

Spectrum S27: Mass spectrum of compound **3c**

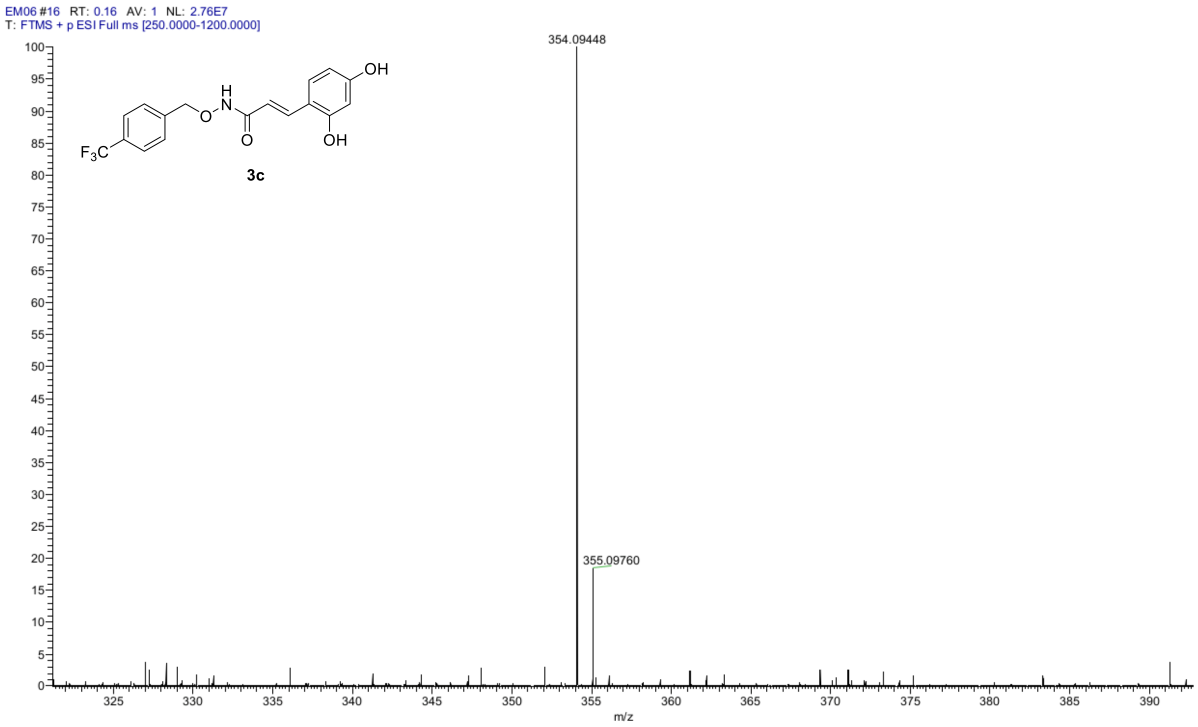

Spectrum S28: ^{1}H-NMR spectrum of compound **4a**

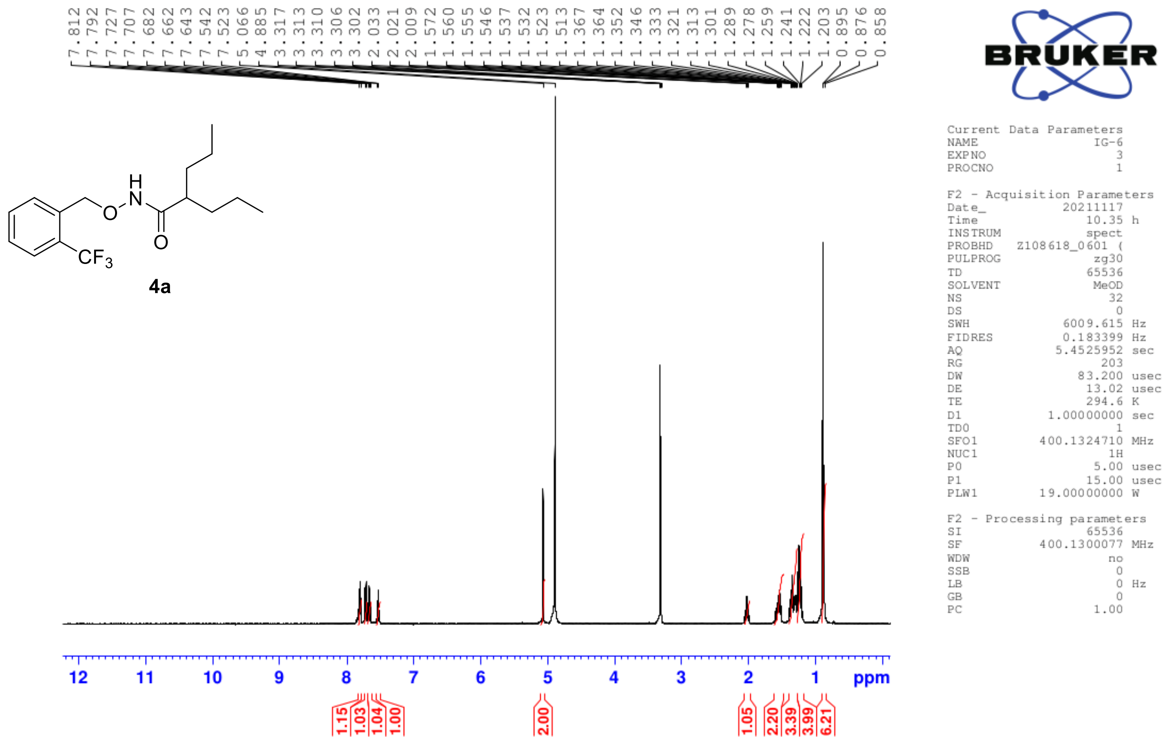

Spectrum S29: ^{13}C-NMR spectrum of compound **4a**

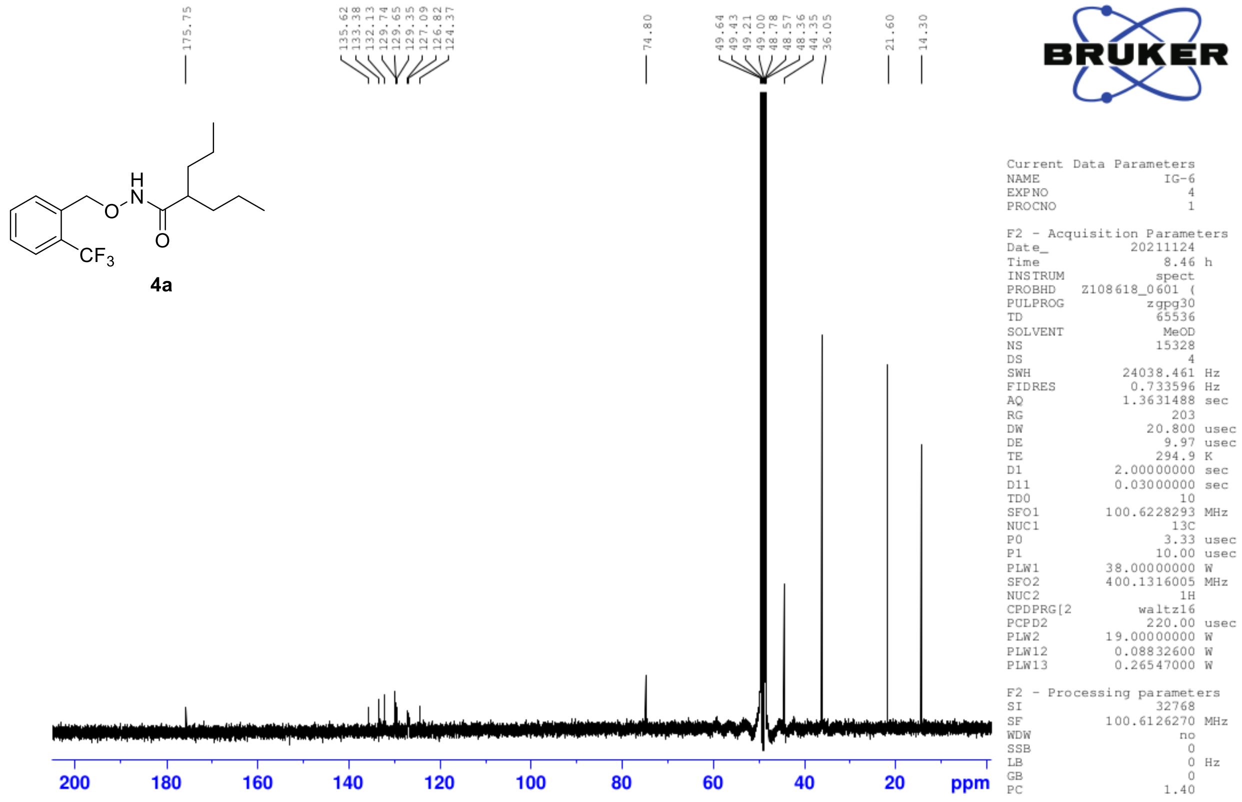

Spectrum S30: Mass spectrum of compound **4a**

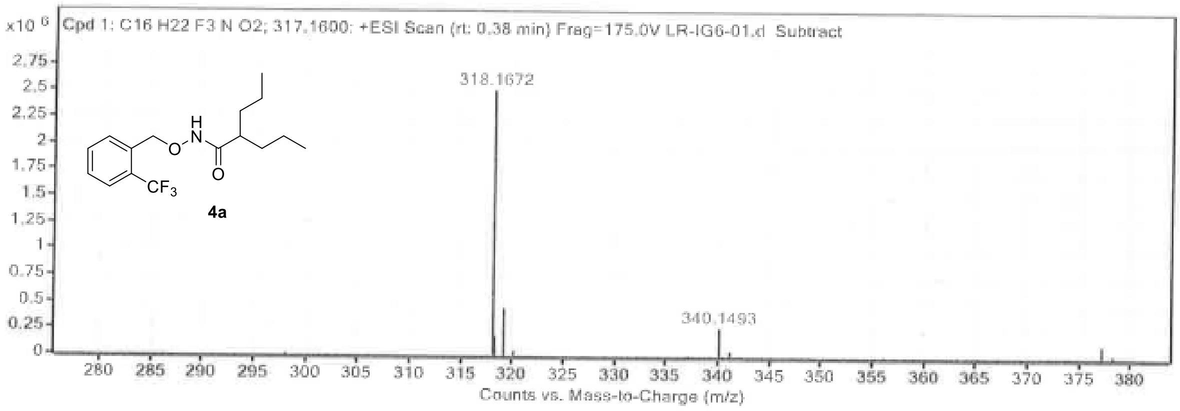

Spectrum S31: ^{1}H-NMR spectrum of compound **4b**

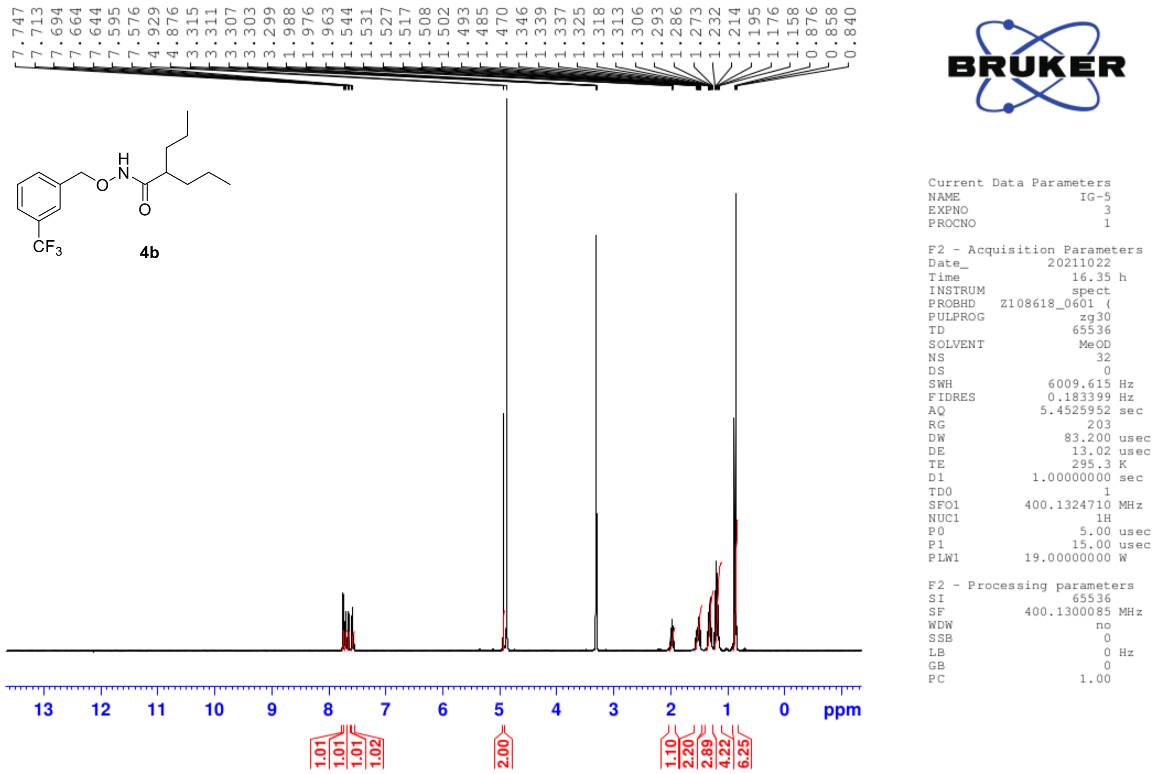

Spectrum S32: ^{13}C-NMR spectrum of compound **4b**

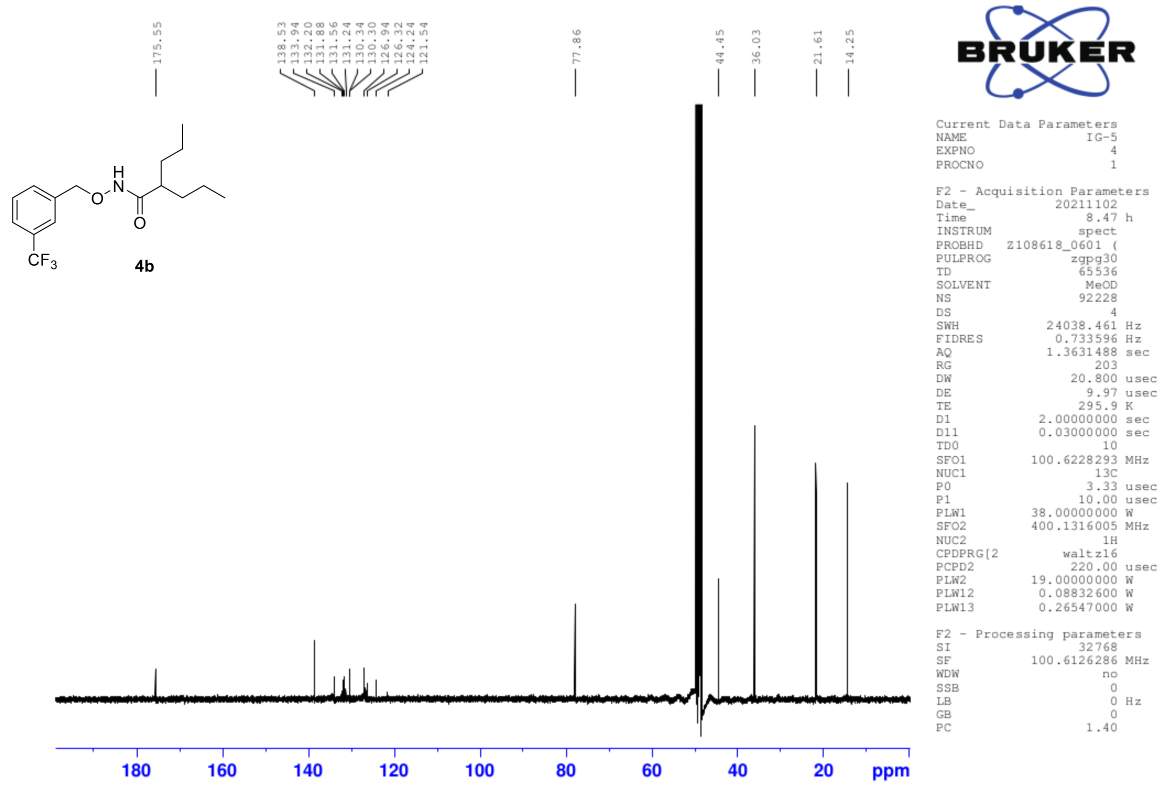

Spectrum S33: Mass spectrum of compound **4b**

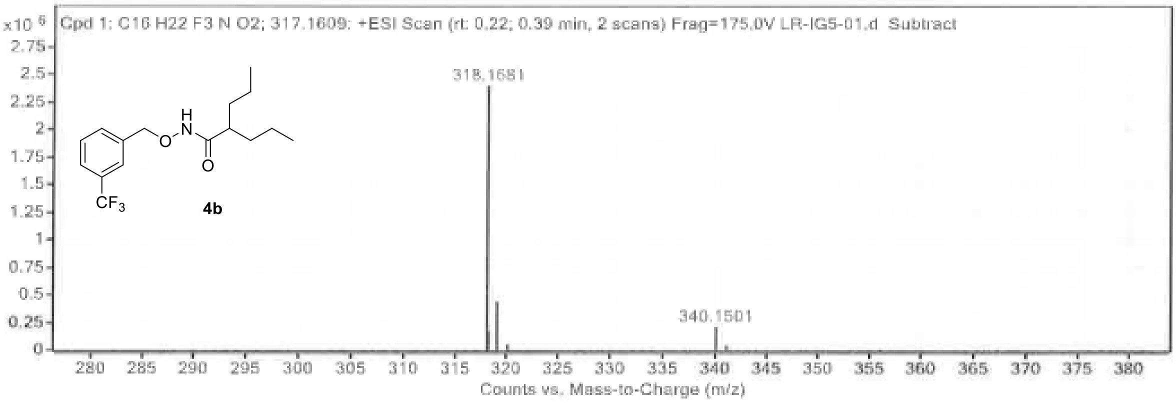

Spectrum S34: ^{1}H-NMR spectrum of compound **4c**

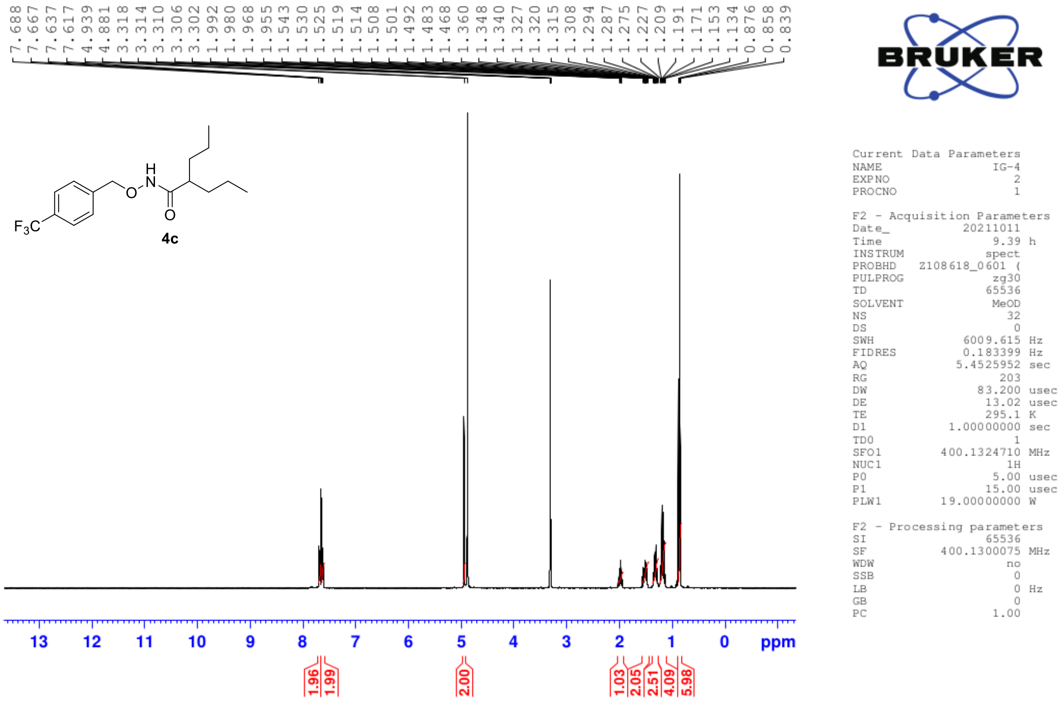

Spectrum S35: ^{13}C-NMR spectrum of compound **4c**

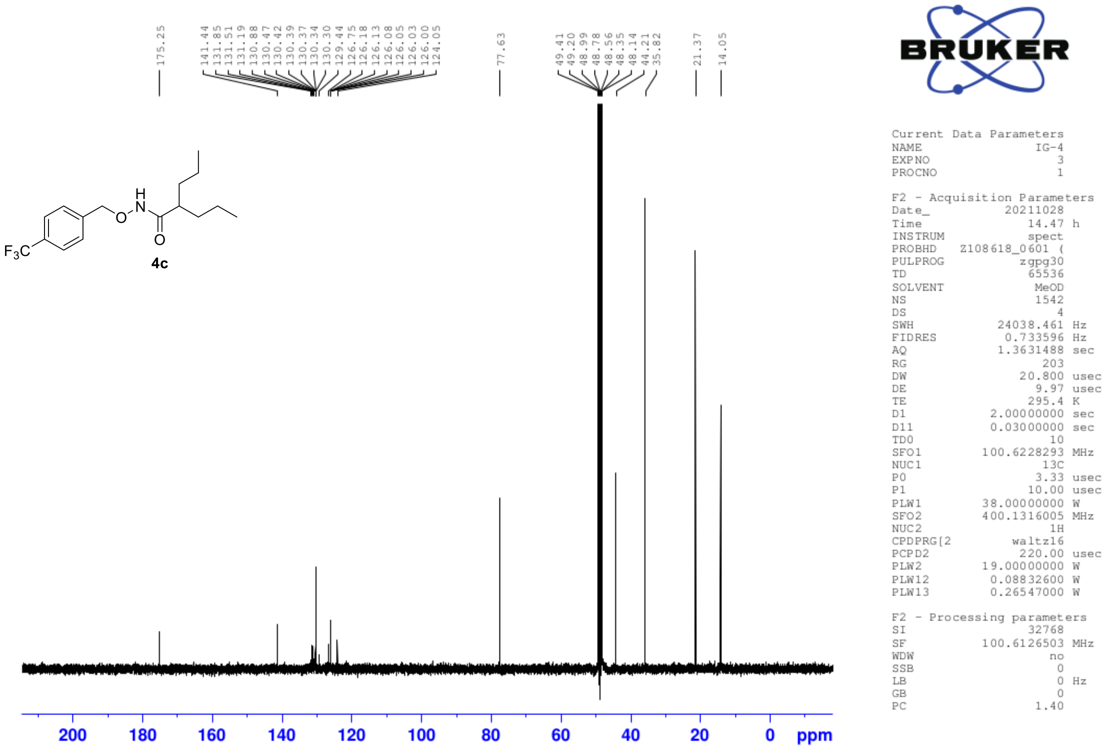

Spectrum S36: Mass spectrum of compound **4c**

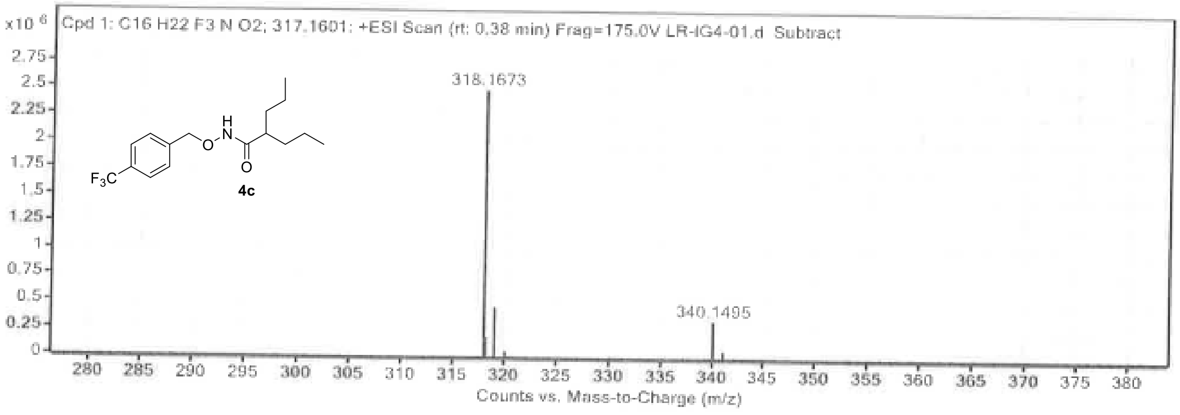

Spectrum S37: ^{1}H-NMR spectrum of compound **5a**

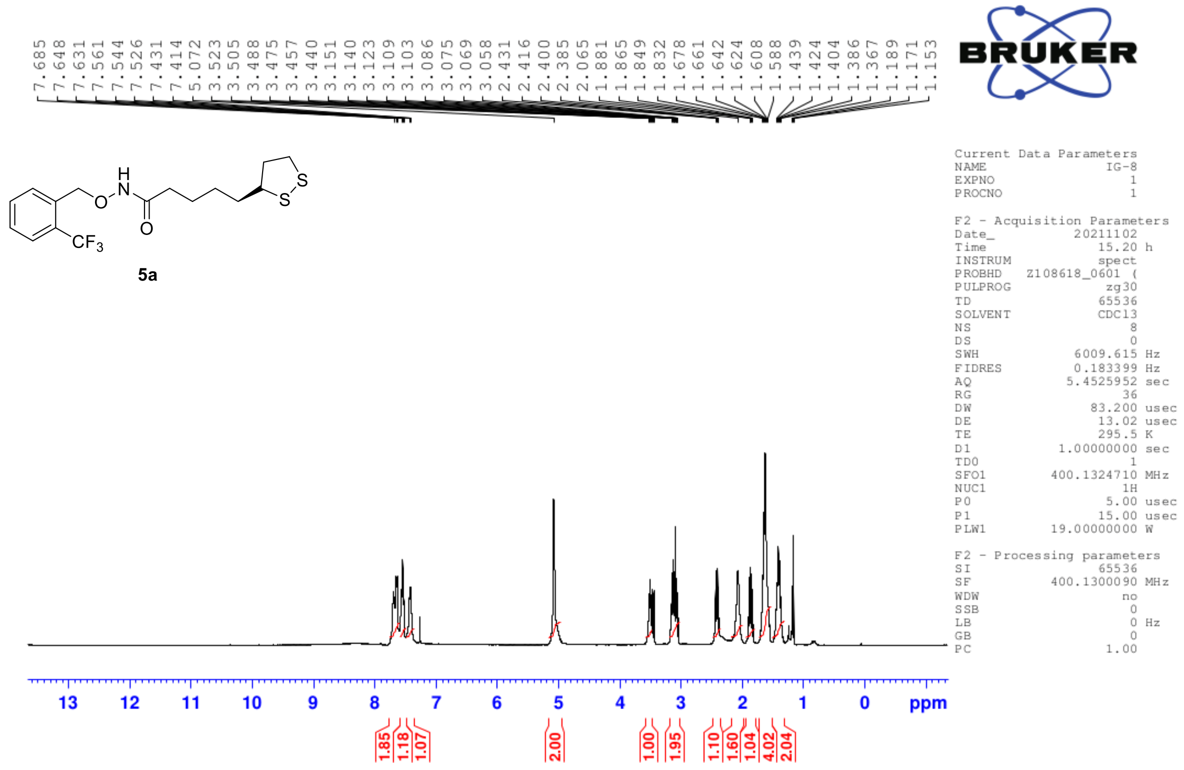

Spectrum S38: ^{13}C-NMR spectrum of compound **5a**

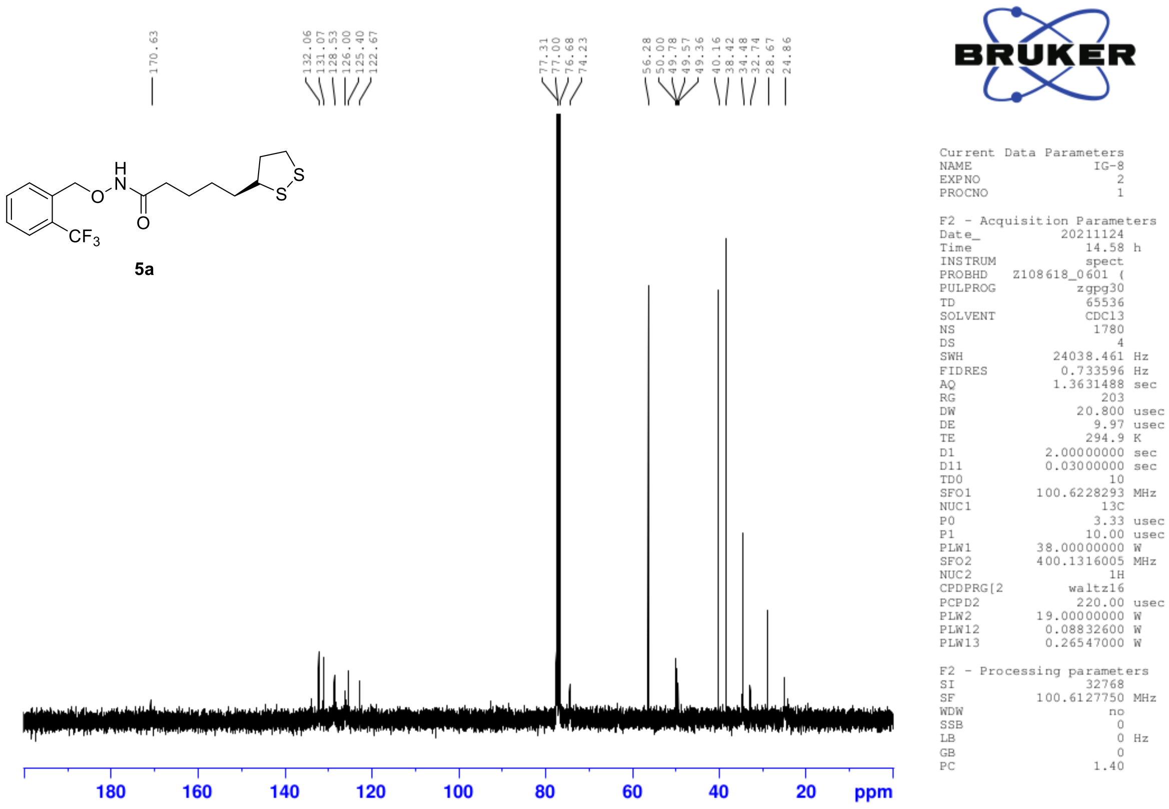

Spectrum S39: Mass spectrum of compound **5a**

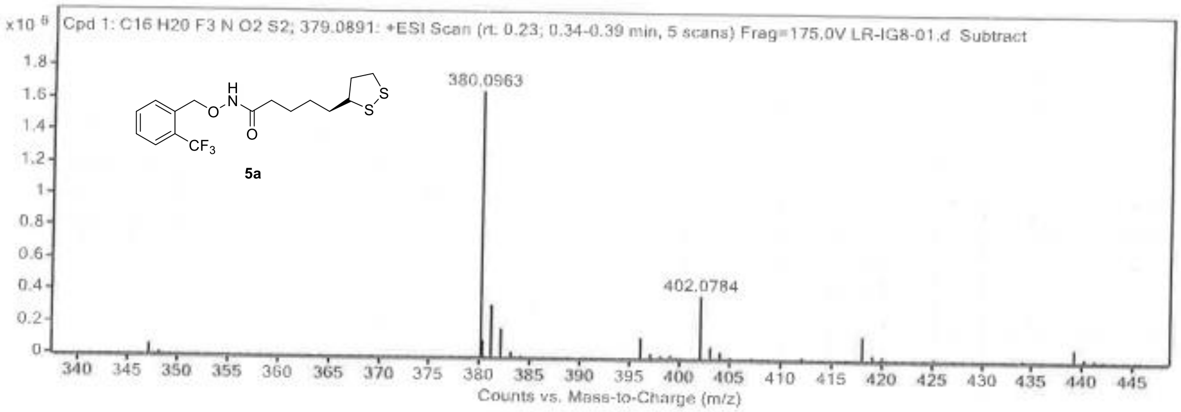

Spectrum S40: ^{1}H-NMR spectrum of compound **5b**

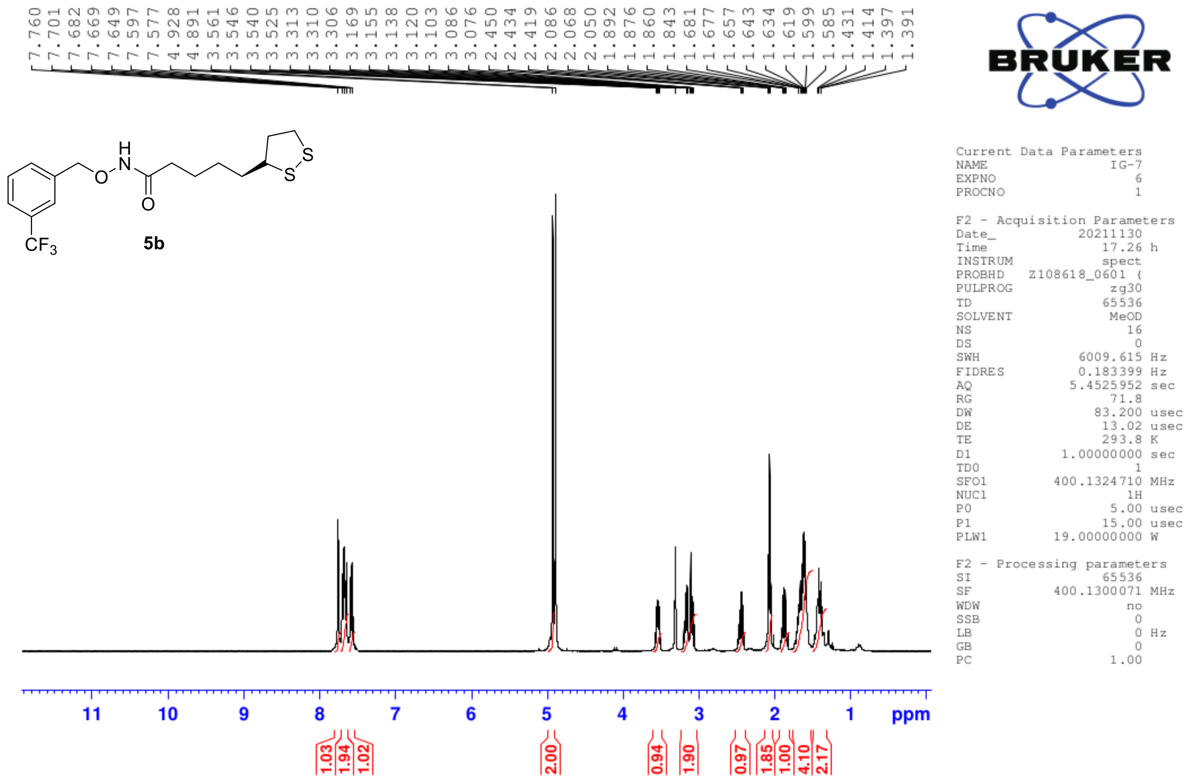

Spectrum S41: ^{13}C-NMR spectrum of compound **5b**

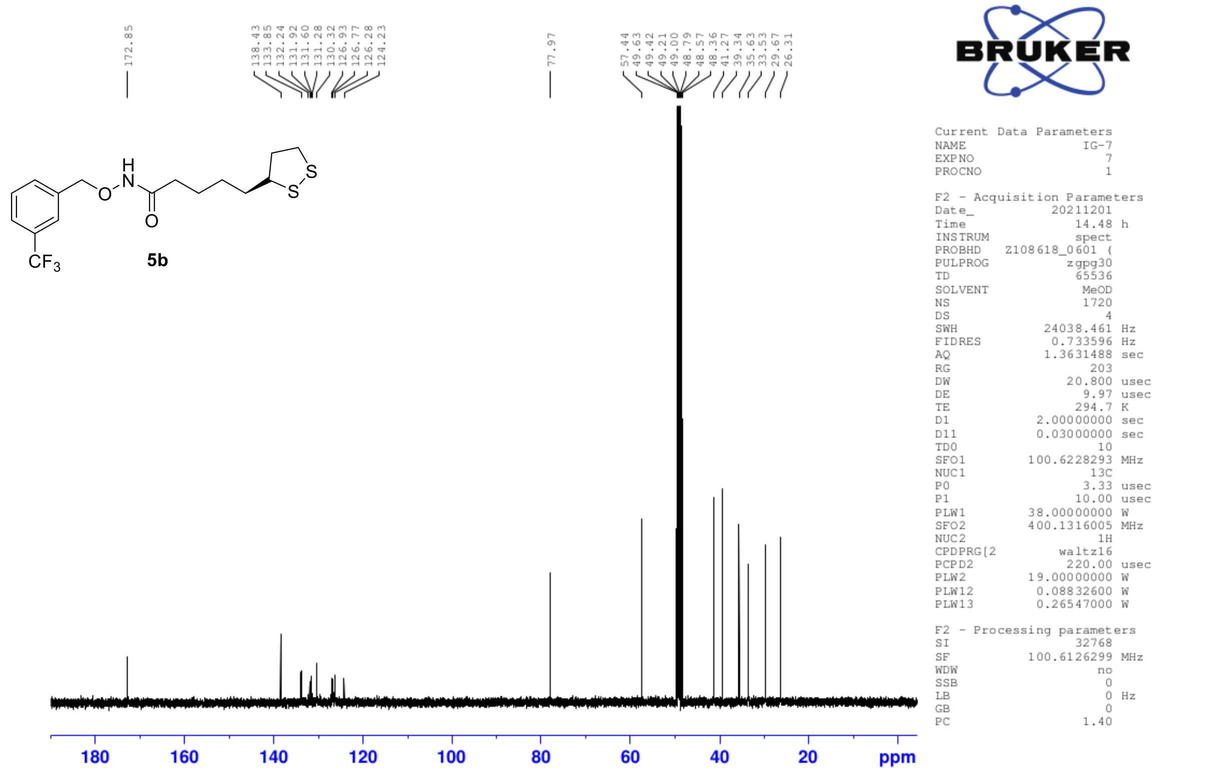

Spectrum S42: Mass spectrum of compound **5b**

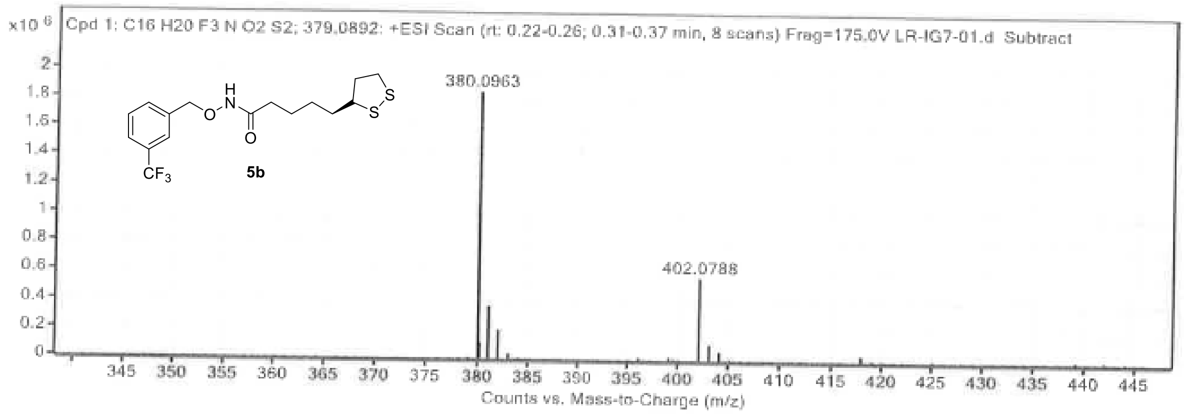

Spectrum S43: ^{1}H-NMR spectrum of compound **5c**

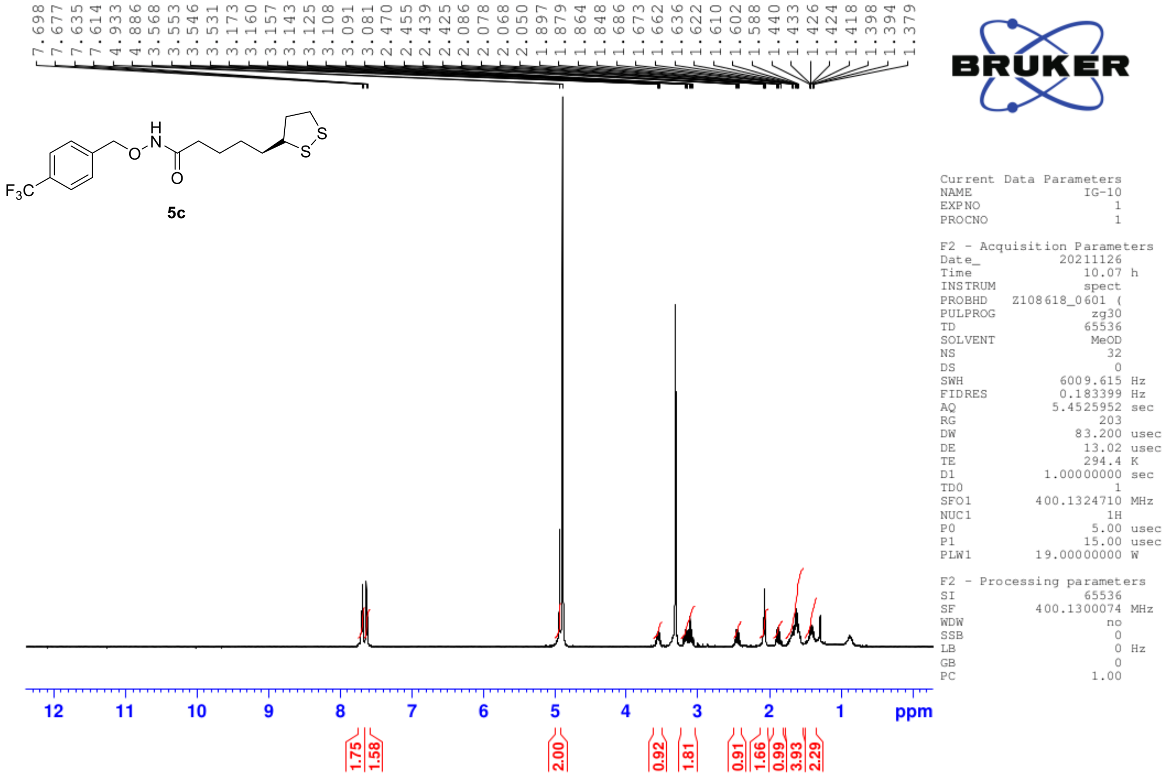

Spectrum S44: ^{13}C-NMR spectrum of compound **5c**

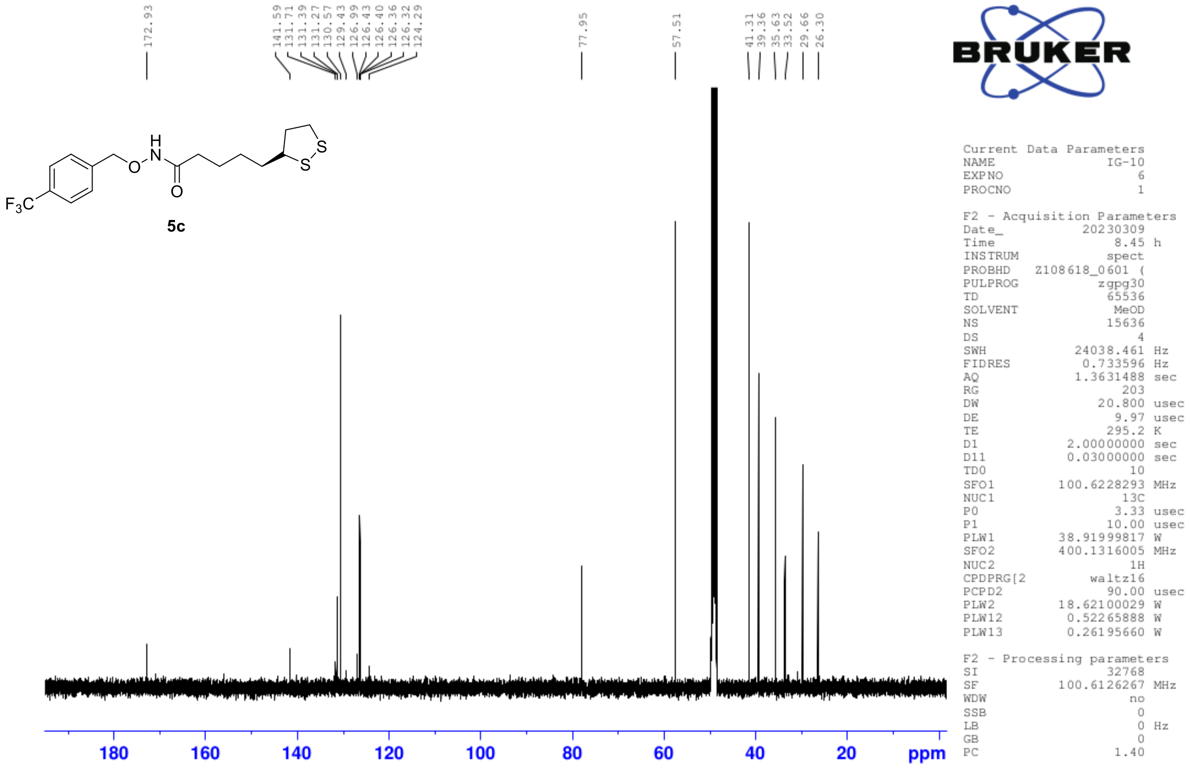

Spectrum S45: Mass spectrum of compound **5c**

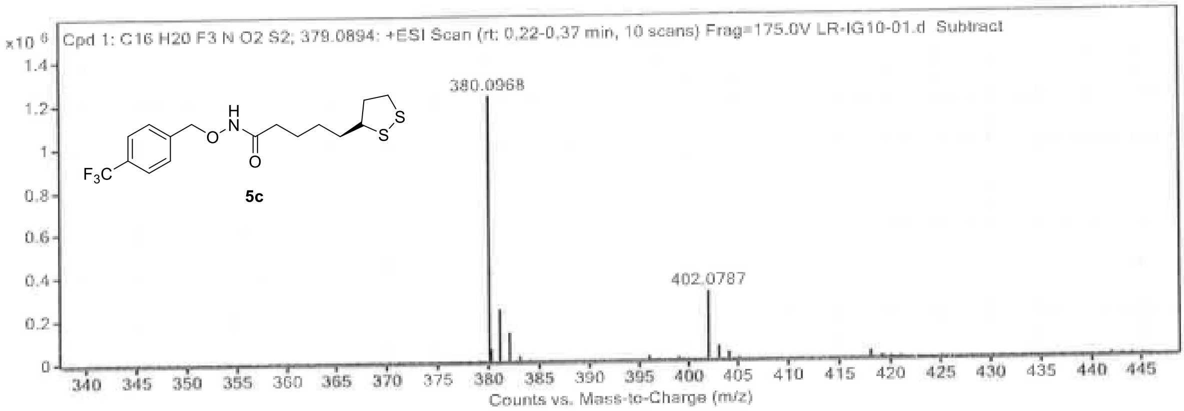

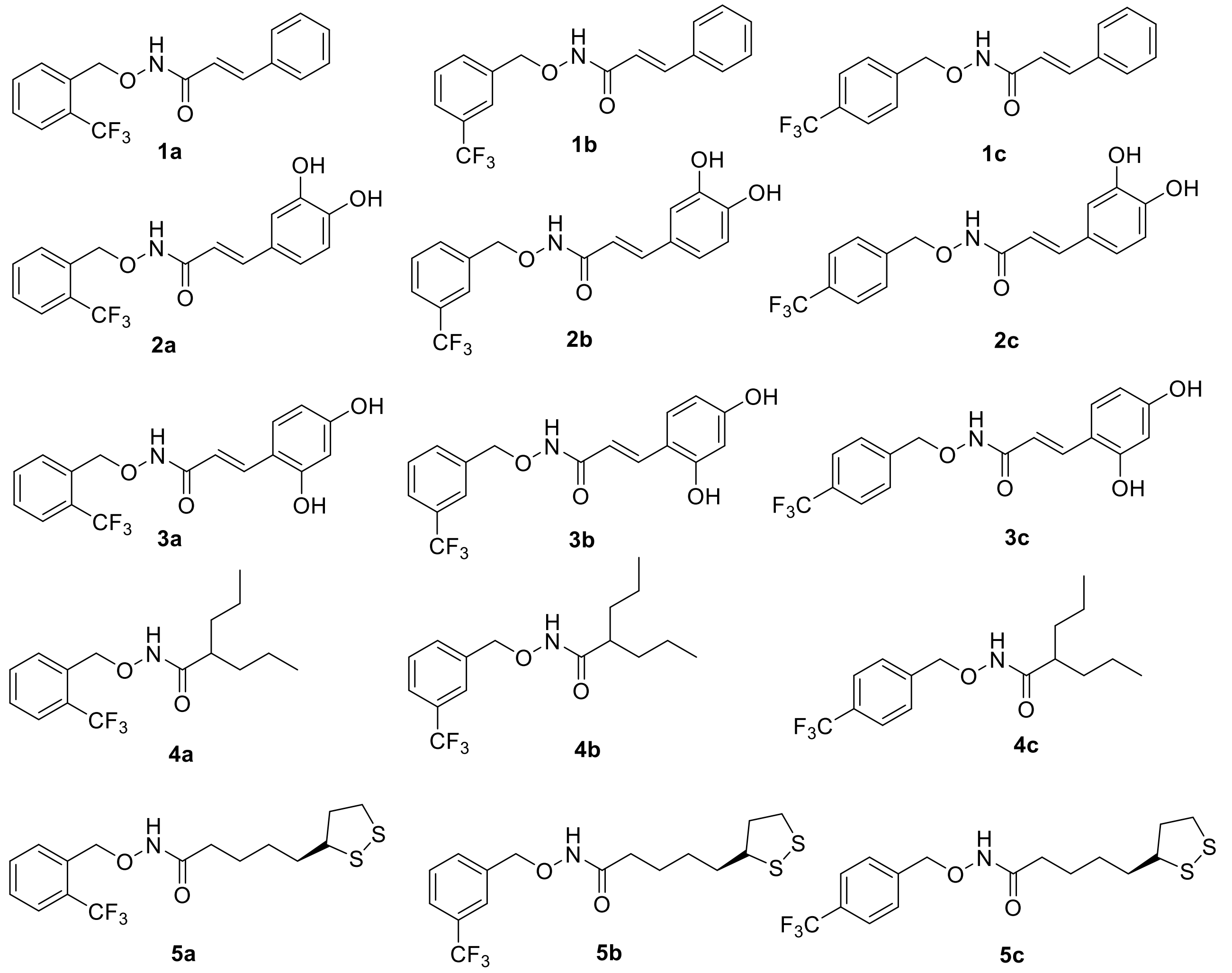

Figure S1 Chemical structure of all synthesized compounds.

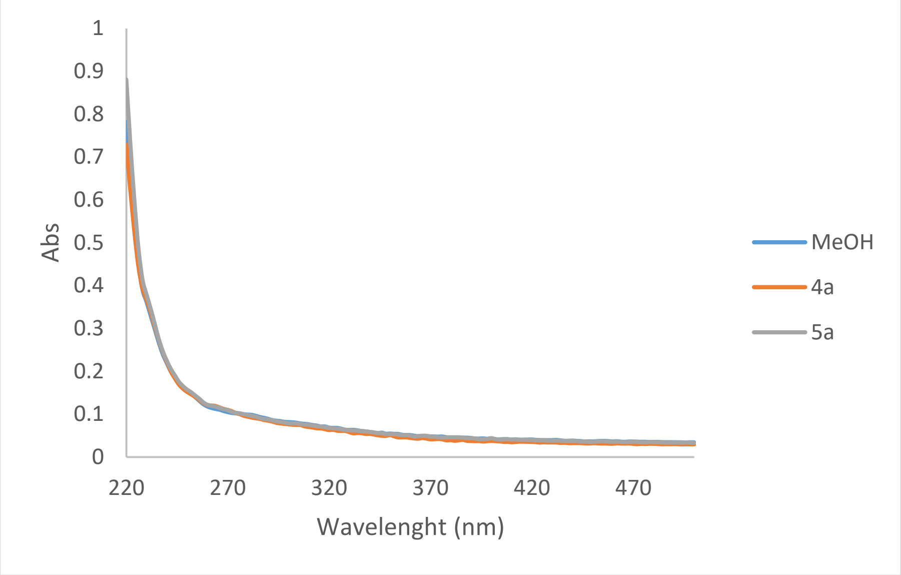

Figure S2 UV Spectra of MeOH, compound **4a** and **5a**.

A

B

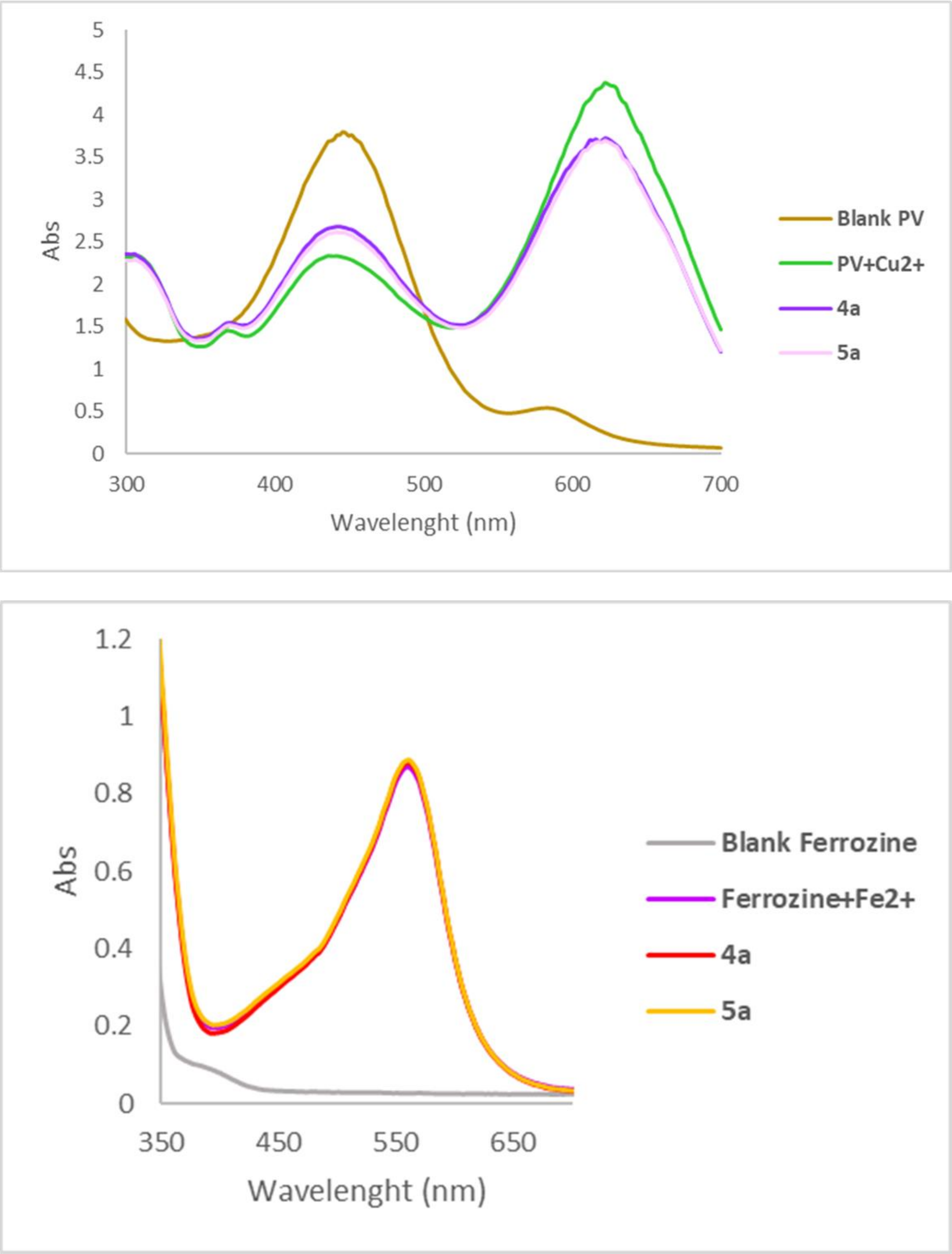

Figure S3 Colorimetric metal chelation study of compounds **4a** and **5a. A** Derivatives **4a** e **5a** in presence of PV and Cu^{2+}; **B** Derivatives **4a** e **5a** in presence of Ferrozine and Fe^{2+}.

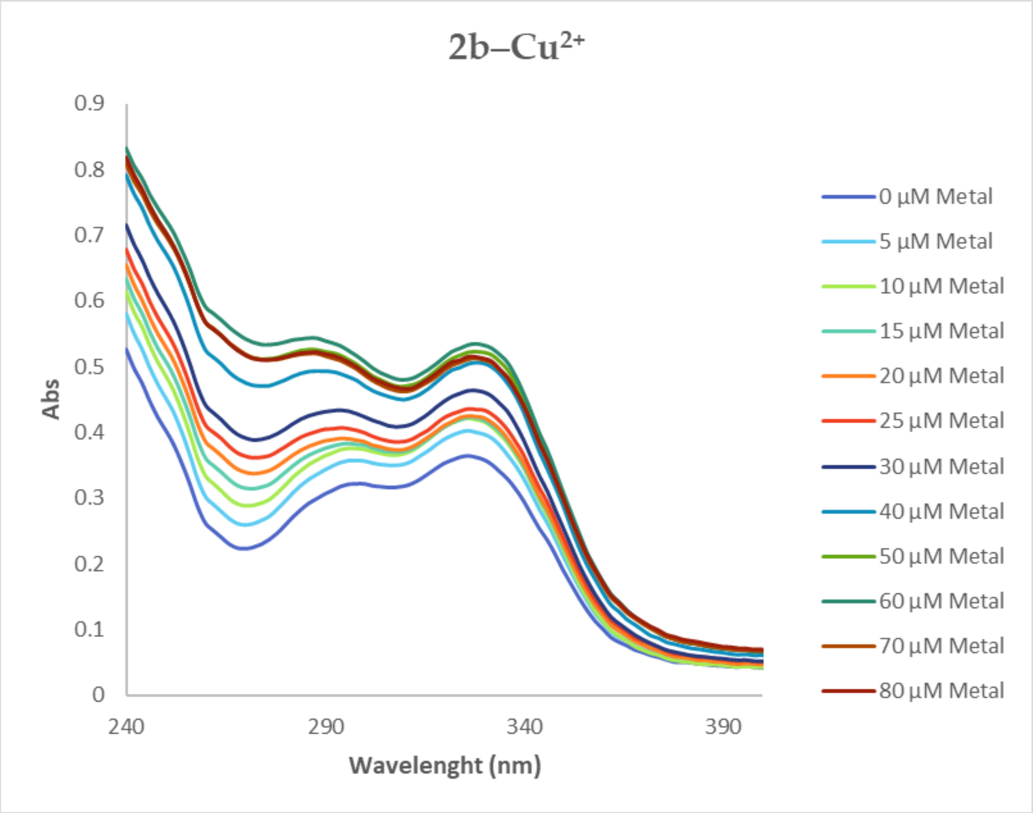

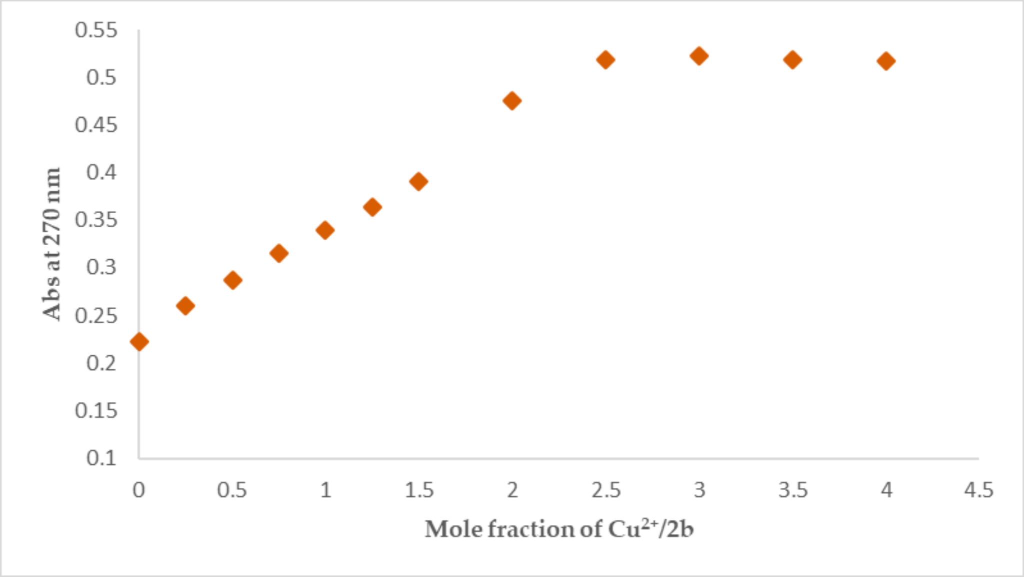

Figure S4 UV Spectra of compound **2c** with increased concentration of Cu^{2+} and molar fraction of the complex.

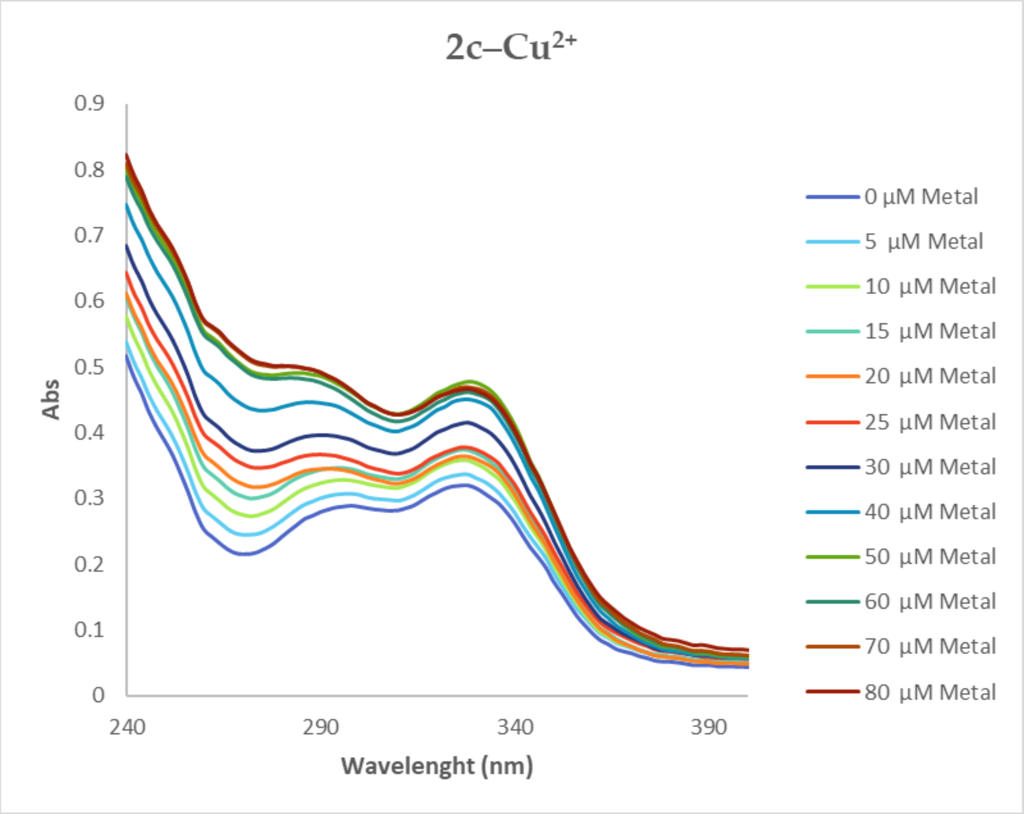

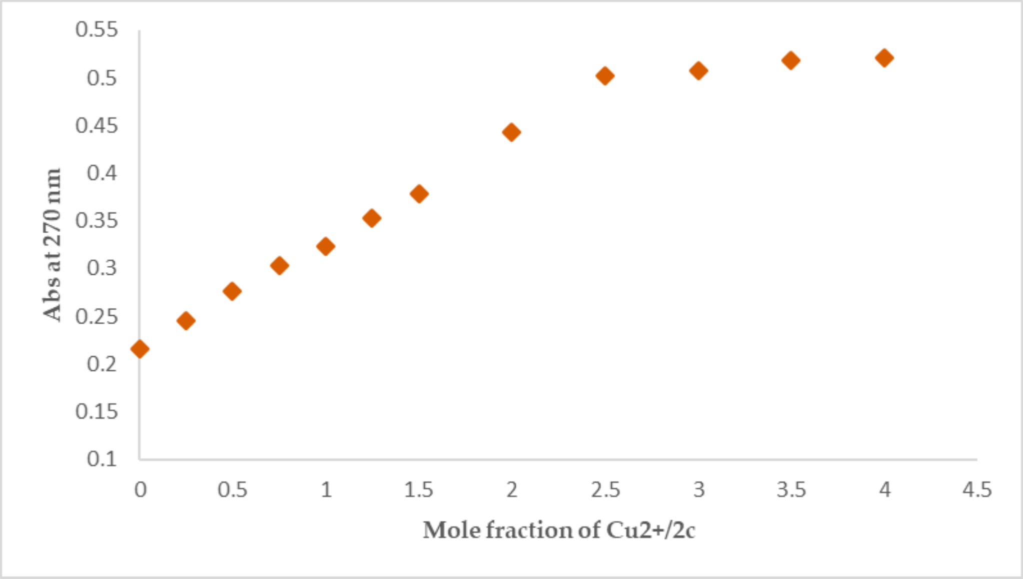

Figure S5 UV Spectra of compound **2c** with increased concentration of Cu^{2+} and molar fraction of the complex.

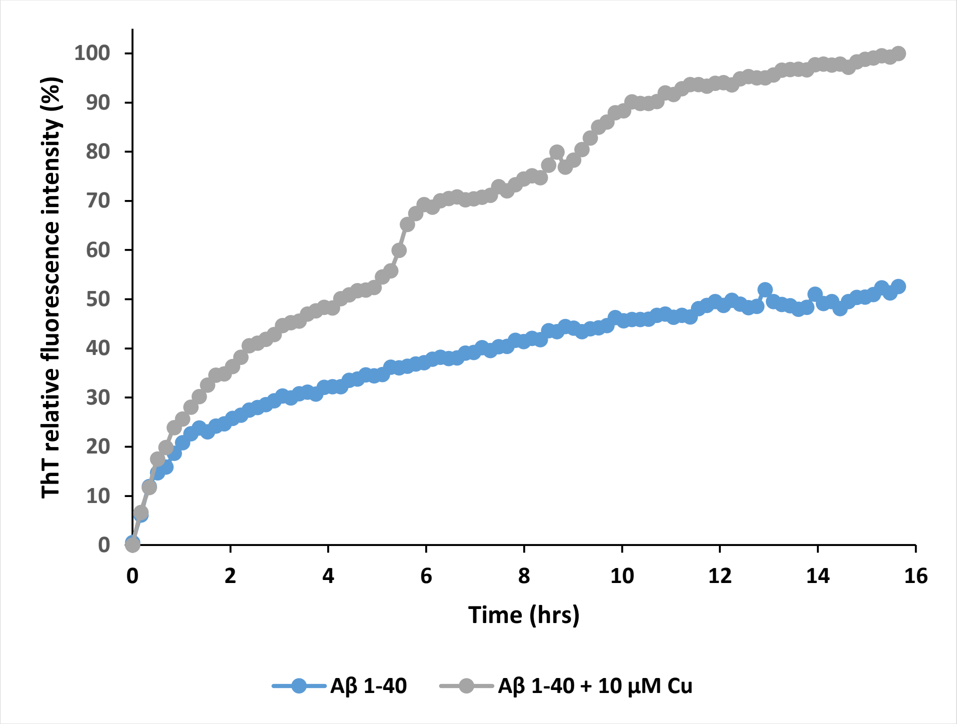

Figure S6 Representative aggregation kinetics curves of $A\beta_{1-40}$ (10 μM) in the absence (blue) or presence (grey) of 10 μM of Cu^{2+}.

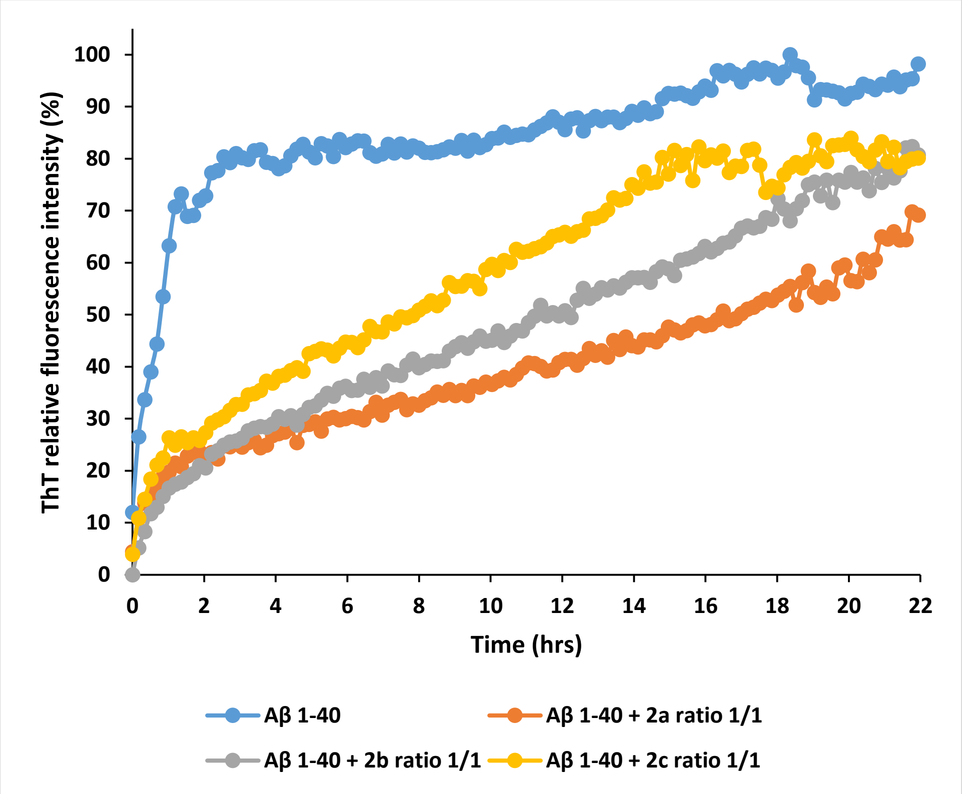

Figure S7 Representative aggregation kinetics curves of $A\beta_{1-40}$ (10 μM) in the absence (blue) or presence (orange, grey and yellow, respectively) of compounds **2a-c** (10 μM).

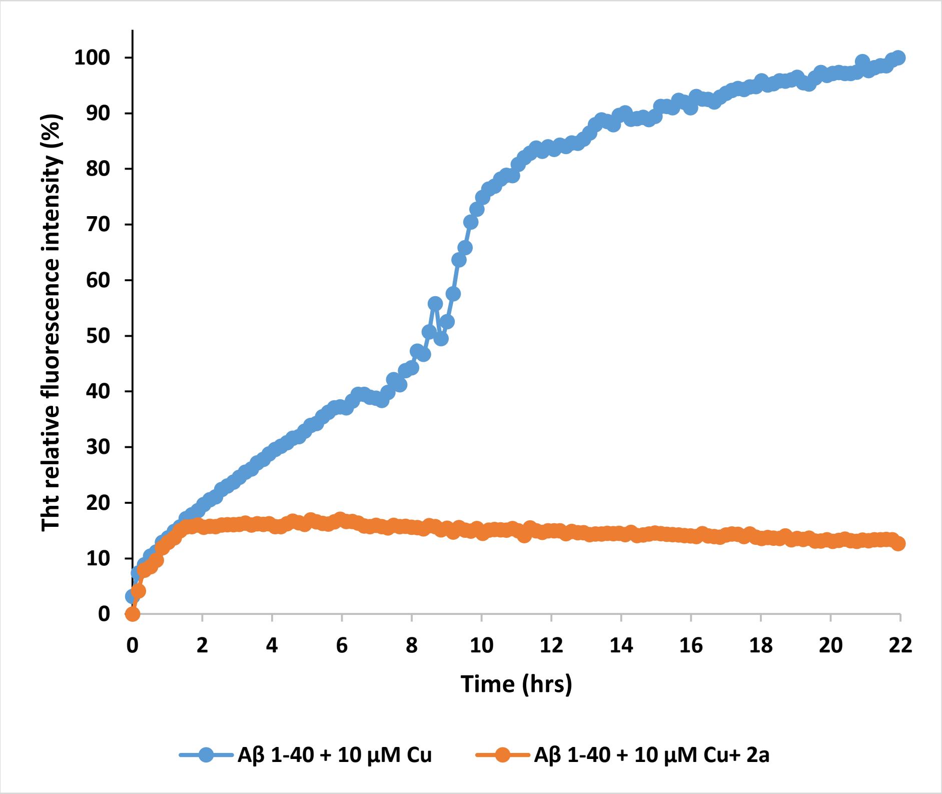

Figure S8 Representative aggregation kinetics curves of $A\beta_{1\text{-}40}$ (10 µM) with 10 µM of Cu^{2+} in the absence (blue) or presence (orange) of compound **2a** (10 µM).

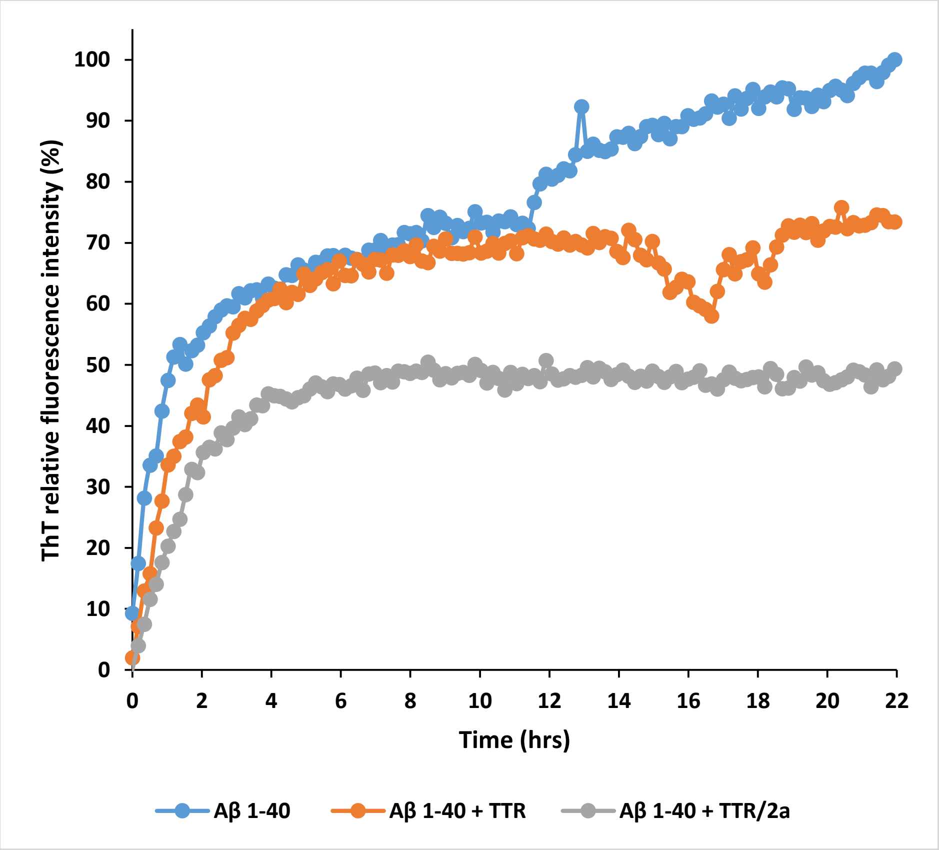

Figure S9 Representative aggregation kinetics curves of $A\beta_{1\text{-}40}$ (10 µM) in the presence (orange) or absence (blue) of 10 µM of TTR with (grey) or without (orange) the pre-incubation with compound **2a** (20 µM).

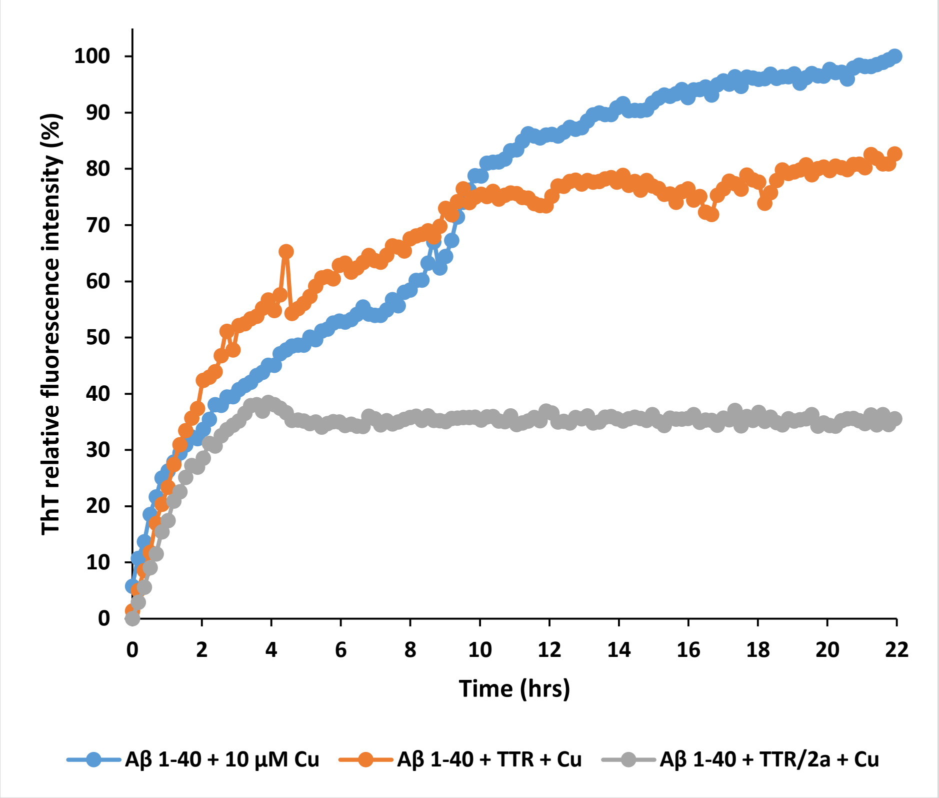

Figure S10 Representative aggregation kinetics curves of Aβ$_{1\text{-}40}$ (10 µM) and 10 µM of Cu^{2+} in the presence (orange) or absence (blue) of 10 µM of TTR with (orange) or without (grey) the pre-incubation with compound **2a** (20 µM).
